# The 26^th^ Scientific Conference of the Society on NeuroImmune Pharmacology: College of Pharmacy, University of Tennessee Health Science Center, Memphis, TN, June 1-3, 2022

**DOI:** 10.1515/nipt-2022-0004

**Published:** 2022-03-25

**Authors:** Santosh Kumar, Chandravanu Dash, Gurudutt Pendyala, Sowmya V. Yelamanchili, Sanjay B. Maggirwar, Jean M. Bidlack, Sulie L. Chang

**Affiliations:** Department of Pharmaceutical Sciences, College of Pharmacy, University of Tennessee Health Science Center, 881 Madison Ave., Memphis, TN, 38163, USA, Phone: +1 (901) 448-7157; Department of Microbiology and Immunology and Center for AIDS Health Disparities Research, Meharry Medical College, Nashville, TN, USA; Department of Anesthesiology, College of Medicine, University of Nebraska Medical Center, Omaha, NE, USA; Department of Microbiology, Immunology, and Tropical Medicine, The George Washington School of Medicine and Health Sciences, Washington, DC, USA; Department of Pharmacology and Physiology, School of Medicine and Dentistry, University of Rochester Medical Center, Rochester, NY, USA; Department of Biological Sciences, Institute of NeuroImmune Pharmacology, Seton Hall University, South Orange, NJ, 07079, USA, Phone: +1 (973) 761-9456

**Keywords:** HIV, neuroHIV, SARS-CoV-2, COVID-19, neuroimmune pharmacology, drugs of abuse, therapeutics, vaccines, early-career investigators, diversity and inclusion

## Abstract

The 26^th^ Scientific Conference of the Society on NeuroImmune Pharmacology (SNIP) at the University of Tennessee Health Science Center in Memphis, Tennessee, June 1-3, 2022, is SNIP’s first full-fledged meeting in person since the onset of the coronavirus disease-19 pandemic. The three-day meeting encompasses a variety of activities that include a pre-conference session, many scientific sessions (eight symposia and two plenary lectures), two special talks, a poster session, oral talks, a mentoring session for early career investigators, a diversity and inclusion SNIP committee session, a business meeting, and an award session. A conference summary, detailed program agenda, accepted poster abstracts, and presentation abstracts are included in this brief report published in advance of the meeting.

## Summary

The Society on NeuroImmune Pharmacology (SNIP) is hosting its 26^th^ Scientific Conference at the University of Tennessee Health Science Center (UTHSC) in Memphis, Tennessee, on June 1-3, 2022. This conference in Memphis is the society’s first in-person annual meeting since the pandemic began, and it covers a wide array of scientific activities including several symposia, sessions for early career investigators (ECI), and other activities. After postponement of the 26^th^ annual meeting, originally planned for 2020 in New Delhi, India, the society organized a SNIP virtual workshop on coronavirus disease-19 (COVID-19) in 2021, in part, to maintain the consistency of SNIP activity. The New Delhi meeting will be held on March 15-18, 2023, as the 27^th^ SNIP annual meeting.

The 26^th^ Scientific Conference begins June 1 with a pre-conference workshop, organized by QIAGEN, covering a technical talk by a QIAGEN scientist followed by a scientific talk by Dr. Dipak Sarkar on implementation of QIAGEN technologies. The meeting is then inaugurated with a warm welcome and greetings from Dr. Steve Goodman, Vice-Chancellor, Research, UTHSC. The inauguration is followed by a poster session for ECI and regular members. The activities on day 1 conclude with a Diversity and Inclusion SNIP Committee (DISC) special talk by Dr. Okeoma Chioma, Professor and Vice-Chair of Research, New York Medical College, followed by several podium presentations by DISC-ECI. The DISC session was organized by DISC chair, Dr. Sowmya V. Yelamanchili, Associate Professor, University of Nebraska Medical Center.

On June 2, the meeting’s second day is formally inaugurated by Dr. Kennard Brown, Executive Vice Chancellor and Chief Operations Officer, UTHSC, followed by Dr. Sulie L. Chang, SNIP President and Professor of Biological Sciences and Director of Institute of NeuroImmune Pharmacology, Seton Hall University. The second day consists of four symposia (described below) and two special talks. The special talks are presented in the memory of Dr. Bill Narayan, a legend in the field, and Dr. Adarsh Kumar, who had been an ambassador of supporting SNIP young investigators. The Bill Narayan Memorial Lecture is delivered by Dr. Yuri Persidsky, a former president of SNIP. He lectures on the role of alcohol and e-cigarettes in blood-brain barrier (BBB) injury. The Adarsh Kumar Lecture is delivered by another longtime member of SNIP, Dr. Shilpa Buch. Her lecture is on the role of exosomes on neuroHIV and opioids comorbidity.

The President Symposium, “SARS-CoV-2: Comorbidities and therapeutics,” includes a plenary lecture by Dr. Colleen Jonsson, Professor and Van Vleet Chair of Excellence in Virology and Director, Regional Biocontainment Laboratory (RBL), followed by four talks that cover a wealth of COVID-19 science and its role in neurological complications. The symposium is co-chaired by Dr. Kendall Bryant, Director, Alcohol and AIDS Research, National Institute on Alcohol Abuse and Alcoholism (NIAAA), and Dr. Jeymohan Joseph, Chief, HIV Neuropathogenesis, Genetics, and Therapeutics Branch, National Institute of Mental Health (NIMH). The plenary talk discusses the lessons learned from the past with SARS-CoV-2 infections followed by the current fate of the pandemic and its future projections. The symposium talks mainly cover the role of SARS-CoV-2 in neurological functions including cerebrovascular pathology, neurological dysfunction, and neuropsychiatric complications, along with a talk on its effect in immunocompromised pediatric populations.

The second symposium of day 2, “Use of iPSC-derived CNS cells and brain organoids to model HIV infection, substance use, and ARV treatment in the CNS,” was organized and co-chaired by Dr. Kelly Jordan-Sciutto, Professor and Associate Dean of Organizational Effectiveness, University of Pennsylvania, and Dr. Wenzhe Ho, Professor, Temple University. The symposium covers four talks that include a senior investigator, two junior investigators, and a graduate student. The symposium focuses on the role of iPSC-derived CNS cells and brain organoids in modeling neuroHIV. The symposium also covers the modeling of neuroHIV in the context of antiretroviral therapy (ART) as well as substance abuse.

The third symposium, “T time for neuroimmune pharmacology research,” was organized and co-chaired by Dr. Marta Catalfamo, Associate Professor, Georgetown University School of Medicine, and Dr. Sanjay B. Maggirwar, Professor and Chair of the Department of Microbiology, Immunology, and Tropical Medicine, The George Washington University. This unique symposium covers the breadth of science funded by the NIH T-series for trainees. The symposium is delivered by graduate students and post-doctorate fellows who talk on their respective research projects, funded by T-series NIH grants. Overall, the symposium discusses the success story of the T-series NIH grants, and it promotes other universities/institutes and investigators to avail themselves of such grants to support their/our trainees.

The final symposium of day 2 was organized by Dr. Howard E. Gendelman, Editor-in-Chief of our newly launched SNIP journal, *NeuroImmune Pharmacology and Therapeutics* (*NIPT*), Professor and Chair, University of Nebraska Medical Center Department of Pharmacology and Experimental Neuroscience. The symposium is co-chaired by Dr. Jean M. Bidlack, Professor, University of Rochester Medical Center. The symposium is titled, “Meet the *NeuroImmune Pharmacology and Therapeutics* (*NIPT*) Editors.” In addition to Dr. Gendelman, the symposium includes four other *NIPT* editors as speakers. All five talks in this symposium revolve around HIV reservoirs in the CNS and their treatment strategies using a variety of technologies. Upon the conclusion of their respective talks, Dr. Gendelman moderates a roundtable discussion for the new journal, *NIPT*; he presents the vision of the new journal and conducts a Q/A session.

Day 3 sessions on June 3 consist of four symposia (described below), an ECI speed mentoring session, a business meeting, and an award ceremony. The ECI speed-mentoring session was organized by the Early Career Investigators Travel Awards (ECITA) chair Dr. Gurudutt Pendyala, Professor, University of Nebraska Medical Center, and co-chaired by Dr. Yisel Cantres-Rosario, Associate Professor, University of Puerto Rico Medical Sciences Campus. This is a unique session to help mentor our ECI, both pre- and post-doctorate fellows by many mentors from different backgrounds (academia, NIH, industry, etc.). The session helps our ECI to learn about themselves and prepare for future endeavors. The business meeting is run by SNIP Secretary, Dr. Jean Bidlack. The meeting invites all SNIP members to propose new ideas, revisit the SNIP Bylaws and Handbook, and make necessary changes as appropriate with approval through membership voting mechanism. The final session is the hallmark of the SNIP annual meeting where we transition from the current councils to the newly elected councils. The session also presents notes of appreciation and announces awards for ECI pre-and post-doctoral fellows in oral presentation and poster categories.

The first session of day 3 begins with the local symposium, organized by the local hosts. The UTHSC and Meharry Medical College (MMC) leadership team organized this session, chaired by Dr. Chandravanu (CV) Dash, Professor, MMC and Dr. Anna Bukia, Professor, UTHSC. The local symposium begins with a plenary talk by Dr. Alex Dopico, Professor and Chair of the Department of Pharmacology, Addiction Science, and Toxicology, UTHSC, titled, “Ionic mechanisms of alcohol-induced brain hypoperfusion.” He is pioneer in the field of alcohol and brain functions and a recipient of the NIH MERIT award. Dr. Dopico discusses the ionic mechanisms that are responsible for alcohol-induced hypoperfusion. Followed by the plenary lecture, the symposium covers four other talks that involve drugs of abuse and their role on brain functions. The talks are delivered by two senior faculties, one junior faculty, and a post-doctoral scholar.

The second symposium is dedicated to ECI, co-chaired by Drs. Pendyala and Cantres-Rosario. The symposium includes five talks each for pre-doctorate and post-doctorate fellows. They present their respective talks in 5 minutes each followed by a collective Q/A session at the end of their talks. The session is the highlight of our ECI training with sharing of their progress towards graduation/fellowship. The best talk from each category will be chosen for “best talk awards,” presented during the award ceremony at the conclusion of the meeting. The ECI talks are another highlight of our meeting.

The third symposium of day 3 focuses on “HIV and neurological diseases.” It is co-chaired by Dr. Syed Ali, Senior Research Scientist, University of Arkansas, President & CEO, NeuroLab International, and Dr. RV Srinivas, Chief, Extramural Project Review Branch, NIAAA. The symposium consists of four speakers, three senior investigators and a graduate student. The symposium highlights various aspects of neuroHIV and its comorbidities in terms of other diseases/conditions and drugs of abuse. Of the four talks, two talks discuss the role of disease comorbidities (stroke and Alzheimer’s disease) and the other two talks discuss drugs of abuse comorbidities (cocaine and morphine) in the context of neuroHIV. Comorbid conditions with HIV are very common, and they severely affect neuroHIV in terms of both pathology and treatment outcomes.

The final symposium was organized by co-chairs Drs. Jerel Fields and Susmita Sil, assistant professors at their respective institutions, The University of California San Diego and University of Nebraska Medical Center. This symposium is also unique in the way that speakers were selected from the abstract submitted by regular SNIP members. Of 23 abstracts, we chose nine speakers for this symposium for a short 5-minute talk each. Thus, the symposium consists of nine speakers from various categories and research fields. Speakers include professors, associate professors, assistant professors, and instructors, along with a research associate. The symposium covers a variety of research topics, ranging from different treatment strategies for neuroHIV, the role of methamphetamine and opioids on neuroHIV and behavior, and other neuronal effects of neuroHIV.

In conclusion, the 26^th^ SNIP annual meeting is our first full-fledged, in-person meeting since the onset of the pandemic. The meeting encompasses a variety of activities that include a pre-conference session, many scientific sessions (eight symposia and two plenary lectures), two special talks, a poster session, oral talks, a mentoring session for ECI, a DISC session, a business meeting, and an award session. The detailed program agenda and abstracts of oral talks and posters are provided below.

**Acknowledgments:** The authors thank the UTHSC leadership team (the office of the executive vice-chancellor and chief operating officer) for providing the space, technologies, and security to run the meeting. The authors also thank Mr. Lee Weever for technology support and Ms. Patrice Shaw for assistance with organizing meeting-related activities. Finally, we thank *NIPT* Managing Editor Mr. Doug Meigs and *NIPT* Associate Managing Editor Ms. Amy Sather (with the University of Nebraska Medical Center’s Department of Pharmacology and Experimental Neuroscience) for helping with the meeting and assembling this conference report.

**Research funding:** QIAGEN and Thermo Fisher Scientific; the Institute of NeuroImmune Pharmacology, Seton Hall University; and the *NIPT* journal have partially sponsored this meeting. The brief report, in part, was supported by the National Institute on Drug Abuse (DA047178 to S Kumar and DA046258 to SL Chang).

**Declaration of interest:** The authors have no relevant affiliations or financial involvement with any organization or entity with a financial interest in or financial conflict with the subject matter or materials discussed in the manuscript. This includes employment, consultancies, honoraria, stock ownership or options, expert testimony, grants or patents received or pending, or royalties.

## Program

The 26^th^ Scientific Conference of SNIP is hosted by the College of Pharmacy (COP), University of Tennessee Health Science Center (UTHSC), Memphis, TN, USA.

All seminars, June 1-3, are in COP 101. The June 1 council meeting is in COP 105. Conference registration, June 1 posters with reception, and daily box lunches are in COP Lobby.

**Table:** 

**Tuesday, May 31, 2022**	
6:00 pm-9:00 pm	Dinner for the Councils (2019-2022), The Peabody Memphis
**Wednesday June 1, 2022 (seminars in COP 101)**	
10:00 am-12:00 pm	**Council meeting (COP 105)**
11:45 am-12:00 pm	**Box lunch (COP Lobby)**
12:00 pm-1:00 pm	**QIAGEN workshop** **Moderator:** Pankaj Seth, PhD, Professor and Scientist VII, National Brain Research Centre, Gurgaon, Haryana, India **Introduction:** Nicole McKiernan, Associate Manager, Account Development Management, QIAGEN Digital Insights, QIAGEN **Speaker:** Araceli Cuellar, PhD Senior Field Application Scientist, QIAGEN Digital Insights, QIAGEN **Title:** Analyze, Compare & Contextualize data with QIAGEN Ingenuity Pathway Analysis **Introduction:** Sulie L. Chang, PhD, Professor of Biological Sciences and Director, Institute of NeuroImmune Pharmacology, President, SNIP, Seton Hall University, South Orange, NJ, USA **Speaker:** Dipak Sarkar, PhD, DPhil, Board of Governors Distinguished Professor, Rutgers, The State University of New Jersey, New Brunswick, NJ 08901-8525 **Title:** Use of Ingenuity Pathway Analysis in systemic review of alcohol epigenetic effects on neuroendocrine axis
1:00 pm-5:00pm	**Registration (COP Lobby)**
1:45 pm-2:00 pm	**Inauguration/welcome: Steven R. Goodman, PhD,** Vice Chancellor for Research, University of Tennessee Health Science Center, Memphis, TN, USA (Introduction by **Santosh Kumar, PhD**, Professor, Pharmaceutical Sciences, Assistant Dean, College of Pharmacy, UTHSC, and President-elect and Interim meeting Chair, SNIP)
2:00 pm-3:30 pm	**Poster session (COP Lobby): Gurudutt Pendyala, PhD**, Robert Lieberman Professor, Department of Anesthesiology, College of Medicine, University of Nebraska Medical Center, Omaha, NE, USA **Reception:** Finger snacks & drinks **(COP Lobby)**
3:30 pm- 5:00 pm	**Diversity and Inclusion SNIP Council (DISC) session:** **Introduction: Sowmya V. Yelamanchili, PhD**, Associate Professor, Department of Anesthesiology, College of Medicine, University of Nebraska Medical Center, Omaha, NE, USA **Guest Speaker: Okeoma Chioma, PhD**, Professor and Vice-Chair of Research, Department of Pathology, Microbiology and Immunology, New York Medical College, New York, NY, USA **Title**: SIV and Δ9-THC induced alterations in host miRNAome: Insights from extracellular vesicles
	**DISC ECI speakers:** **1. Mai Mostafa, PhD,** Post-Doctoral Research Associate, University of Nebraska Medical Center, Omaha, NE, USA **Title:** Monocyte biomarkers define sargramostim treatment outcomes for Parkinson’s disease **2. Yaa Fosuah Abu**, Graduate Student, University of Miami, Miami, FL, USA **Title:** Brief hydromorphone exposure during pregnancy sufficient to induce maternal and neonatal microbial dysbiosis **3. Adrian Flores,** Graduate Student, University of Nebraska Medical Center, Omaha, NE, USA **Title:** An integrated systems approach to decode the impact of adolescent nicotine exposure in pre- and postnatal oxycodone exposed offspring **4. Caela Long,** Graduate Student, University of Pennsylvania, PA, USA **Title:** HIV pre-exposure prophylaxis (PrEP) inhibits oligodendrocyte differentiation **5. Abiola Oladapo,** Graduate Student, University of Nebraska Medical Center, Omaha, NE, USA **Title:** Role of astrocyte-specific NLRP6 inflammasome in methamphetamine-mediated neuroinflammation **6. Lester Rosario-Rodriguez, PhD,** Post-Doctoral Fellow, University of Puerto Rico Medical Sciences Campus, PR, USA
	**Title:** CB2R agonist JWH-133 decreases CATB secretion and neurotoxicity from HIV-infected macrophages by attenuating NF-κB activation, oxidative stress, and lysosomal exocytosis **7. Yemmy Soler,** Graduate Student, Florida International University, Miami, FL, USA **Title:** Serpin-derived peptide for the treatment against HIV in the CNS **8. Michael Mora Stangis,** Graduate Student, University of Miami, Miami, FL, USA **Title:** Methamphetamine and the S1 subunits of SARS-CoV-2 variant spike proteins dysregulate human brain endothelial cells
**Thursday June 2, 2022 (seminars in COP 101)**	
8:00 am-8:15 am	**Inauguration/welcome: Kennard Brown, JD, MPA, PhD, FACHE,** Executive Vice Chancellor and Chief Operations Officer, The University of Tennessee Health Science Center (Introduction by **Santosh Kumar, PhD**, Professor, Pharmaceutical Sciences, Assistant Dean, College of Pharmacy, University of Tennessee Health Science Center, President-elect, Interim meeting chair, SNIP) **SNIP President: Sulie L. Chang, PhD**, Professor of Biological Sciences and Director, Institute of NeuroImmune Pharmacology, President, SNIP, Seton Hall University, South Orange, NJ, USA
8:15 am-8:50 am	**President Symposium: SARS-CoV-2: Comorbidities and therapeutics** **Plenary Lecture** **Introduction: Santosh Kumar, PhD,** Professor and Assistant Dean, Department of Pharmaceutical Sciences, College of Pharmacy and Department of Pharmacology, Addiction Science, and Toxicology, College of Medicine, The University of Tennessee Health Science Center, Memphis, TN, USA **Speaker: Colleen B. Jonsson, PhD**, Professor and Van Vleet Chair of Excellence in Virology, Director, Regional Biocontainment Laboratory (RBL), Director, Institute for the Study of Host-Pathogen Systems, The University of Tennessee Health Sciences Center, Memphis, TN, USA **Title**: Past, present, and future of SARS-CoV-2 therapeutics
8:50 am-9:55am	**President Symposium: SARS-CoV-2: Comorbidities and therapeutics** **Co-Chairs:** **Kendall Bryant, PhD, Director,** Alcohol and AIDS Research, National Institute on Alcoholism and Alcohol Addiction (NIAAA), National Institute of Health, Bethesda, MD, USA **Jeymohan Joseph, PhD,** Chief, HIV Neuropathogenesis, Genetics, and Therapeutics Branch, National Institute of Mental Health (NIMH), National Institute of Health, Bethesda, MD, USA **Speakers:** **1. Michal Toborek, MD, PhD,** Professor and Vice-Chair for Research, Department of Biochemistry and Molecular Biology, Miller School of Medicine, University of Miami, Miami, FL, USA **Title:** Cerebrovascular pathology of SARS-CoV-2 infection **2. Miguela Caniza, MD, PhD**, Member, Department of Infectious Disease, St. Jude Graduate School of Biomedical Science, Director, Global Infectious Disease Program, St. Jude Children’s Research Hospital, Professor, The University of Tennessee Health Science Center, Memphis, TN, USA **Title:** COVID-19 in pediatric immunocompromised hosts **3. Siddappa Byrareddy, PhD**, Professor and Vice-Chair of Research, Department of Pharmacology and Experimental Therapeutics, College of Medicine, University of Nebraska Medical Center, Omaha, NE, USA **Title:** SARS-CoV-2 infection leads to neurological dysfunction **4. Scott Letendre, MD,** Professor, Departments of Medicine and Psychiatry, University of San Diego California, San Diego, CA, USA **Title: S**ARS-CoV-2: Neuropsychiatric complications
9:55 am-10:20 am	**Coffee/tea break/networking**
10:20 am-11:25 am	**Symposium 1: Use of iPSC-derived CNS cells and brain organoids to model HIV infection, substance use, and ARV treatment in CNS** **Co-Chairs:** **Kelly Jordan-Sciutto, PhD,** Professor, Associate Dean of Organizational Effectiveness, Department of Oral Medicine, School of Dental Medicine, University of Pennsylvania, Philadelphia, PA, USA **Wenzhe Ho, MD, MPH,** Professor, Department of Pathology and Laboratory Medicine, Lewis Katz School of Medicine, Temple University, Philadelphia, PA, USA
	**Speakers:** **1. Cagla Akay-Espinoza, MD,** Research Assistant Professor, Department of Oral Medicine, School of Dental Medicine, University of Pennsylvania, Philadelphia, PA, USA
	**Title:** iPSC-based platforms reveal new opportunities for modeling HIV infection in the CNS **2. Kimberly Christian, PhD,** Research Assistant Professor, Department of Neuroscience, Perelman School of Medicine, University of Pennsylvania, Philadelphia, PA, USA
	**Title:** Evaluating the impact of antiretroviral drugs in a forebrain organoid model of cortical development **3. Ronald P. Hart, PhD**, Professor, Department of Cell Biology & Neuroscience, Rutgers University, Piscataway, NJ, USA **Title:** AUD-associated, noncoding SNP variants of KCNJ6 alter physiology in iPSC-derived neurons **4. Brittany Bodnar, PhD candidate,** Center for Metabolic Disease Research, Department of Pathology and Laboratory Medicine, Lewis Katz School of Medicine at Temple University, Philadelphia, PA, USA **Title:** HIV infection of human iPSC-derived microglia-containing cerebral organoids
11:30 am-12:00 pm	**Box lunch (COP Lobby)**
12:00 pm-12:35 pm	**Bill Narayan Memorial Lecture** **Introduction: Santhi Gorantla, PhD,** Professor, Department of Pharmacology and Experimental Neuroscience, College of Medicine, University of Nebraska Medical Center, Omaha, NE, USA **Speaker: Yuri Persidsky, MD, PhD,** Professor and Chair, Department of Pathology and Laboratory Medicine, Lewis Katz School of Medicine at Temple University, Philadelphia, PA, USA **Title:** Alcohol and electronic cigarettes cause blood brain barrier (BBB) injury via purinergic receptor signaling
12:35 pm-1:10 pm	**Dr. Adarsh Kumar Lecture** **Introduction: Nazira El-Hage, PhD,** Associate Professor, Co-Director of the Graduate Program, Immunology & Nano-Medicine, Herbert Wertheim College of Medicine, Florida International University, Miami, FL, USA **Speaker: Shilpa Buch, PhD,** Professor, Department of Pharmacology and Experimental Neuroscience, College of Medicine, University of Nebraska Medical Center, Omaha, NE, USA **Title:** Multifaceted roles of exosomes in neuroHIV & opioid comorbidity
1:10 pm- 1:25 pm	**Bio-break**
1:30 pm- 2:35 pm	**Symposium 2: T time for neuroimmune pharmacology research** **Co-Chairs:** **Marta Catalfamo, PhD,** Associate Professor, Department of Microbiology and Immunology, Georgetown University School of Medicine, Washington, DC, USA **Sanjay B. Maggirwar, PhD, MBA,** Professor and Chair, Department of Microbiology, Immunology, and Tropical Medicine, School of Medicine and Health Sciences, The George Washington University, Washington, DC, USA
	**Speakers:** **1. Andrew Speidell, MS,** Graduate Student, Department of Neuroscience, Georgetown University Medical Center, Washington, DC, USA **Title:** Loss of forebrain cholinergic neurons in aged HIV-1 gp120 transgenic rodents is mediated through the p75NTR neurotrophin receptor **2. Angelo Mandarino, PhD,** Post-doctoral Fellow, Department of Pediatric Hematology/Oncology, Albert Einstein College of Medicine, Bronx, NY, USA **Title:** Effect of methamphetamine on establishment of reactivation of HIV-1 latency in myeloid cell lines and iPSC-derived microglia **3. Rebecca Warfield, PhD candidate**, Department of Neuroscience, Lewis Katz School of Medicine, Temple University, Philadelphia, PA, USA **Title**: Alterations in peripheral nociceptive signaling circuits during HIV/SIV infection contribute to the development of HIV-associated distal sensory polyneuropathy **4. Julie Joseph, Graduate Student**, Department of Microbiology and Immunology, Drexel University College of Medicine, Philadelphia, PA, USA **Title**: Role of exosomes in neuroinflammation caused by retroviral infection
2:35 pm- 3:00 pm	**Coffee/tea break/networking**
3:00 pm- 5:00 pm	**Symposium 3: Meet the *NeuroImmune Pharmacology and Therapeutics* (*NIPT*) Editors** **Co-Chairs:** **Howard Gendelman, MD**, Margaret R. Larson Professor of Internal Medicine and Infectious Diseases, Chair, Department of Pharmacology and Experimental Neuroscience, Department of Pharmacology and Experimental Therapeutics, College of Medicine, University of Nebraska Medical Center, Omaha, NE, USA **Jean M. Bidlack, PhD,** Professor and Associate Chair, Department of Pharmacology and Physiology, School of Medicine and Dentistry, University of Rochester Medical Center, Rochester, NY, USA
	**Speakers:** **1. Howard Gendelman, MD,** Margaret R. Larson Professor of Internal Medicine and Infectious Diseases, Chair, Department of Pharmacology and Experimental Neuroscience, Department of Pharmacology and Experimental Therapeutics, College of Medicine, University of Nebraska Medical Center, Omaha, NE, USA
	**Title:** Strategies for an HIV cure **2. Eliseo Eugenin, MD, PhD,** Associate Professor, William D. Willis Professorship in Neuroscience, Department of Neuroscience, School of Medicine, University of Texas Medical Branch, Galveston, TX, USA **Title:** The current status of the viral reservoirs within the CNS
	**3. Linda Chang, MD**, Professor, Vice Chair for Faculty Development, Department of Radiology and Nuclear Medicine, School of Medicine, University of Maryland, Baltimore, MA, USA **Title:** Brain abnormalities in patients with post-acute sequelae of COVID-19 (PASC) **4. Sabita Roy, PhD**, Professor, Biochemistry & Molecular Biology, Miller School of Medicine, University of Miami, Miami, FL, USA **Title:** Substance use disorder and gut brain axis **5. Kalipada Pahan, PhD**, Professor, Neurological Sciences, Biochemistry and Pharmacology Floyd A. Davis, MD, Endowed Chair, Department of Neurology, Research Scientist, Jesse Brown VA Medical Center, Rush University, Chicago, IL, USA **Title:** Stop paying tolls in the CNS for halting neurodegenerative diseases **Roundtable discussion for the new journal, *NeuroImmune Pharmacology and* Therapeutics** **Moderator: Howard Gendelman, MD** **Howard Gendelman, MD:** Vision for the new SNIP journal, *NeuroImmune Pharmacology and Therapeutics* (*NIPT*)
**Friday June 3, 2022 (seminars in COP 101)**	
8:00 am- 8:35 am	**Local Plenary Lecture** **Introduction: Chandravanu (CV) Dash, PhD,** Professor, Microbiology and Immunology and Center for AIDS Health Disparities Research, Meharry Medical College, Nashville, TN, USA **Speaker: Alejandro (Alex) Dopico, PhD**, Distinguished Professor and Chair, Department of Pharmacology, Addiction Science, and Toxicology, The University of Tennessee Health Science Center, Memphis, TN, USA **Title:** Ionic mechanisms of alcohol-induced brain hypoperfusion **– focus on BK channels.**
8:35 am- 9:40 am	**Local Symposium** **Co-Chairs:** **Anna Bukiya, PhD,** Professor, Department of Pharmacology, Addiction Science, and Toxicology, The University of Tennessee Health Science Center, Memphis, TN, USA **Chandravanu (CV) Dash, PhD**, Professor, Microbiology and Immunology and Center for AIDS Health Disparities Research, Meharry Medical College, Nashville, TN, USA **Speakers:** **1. Brad Grueter, PhD**, Associate Professor, Department of Pharmacology, Vanderbilt University, Nashville, TN, USA **Title:** Kappa opioid receptor modulation of nucleus accumbens microcircuits **2. Jui Pandhare, PhD**, Assistant Professor, Microbiology and Immunology and Center for AIDS Health Disparities Research, Meharry Medical College, Nashville, TN, USA **Title:** Molecular interactions between drug abuse and HIV-1 neuropathogenesis **3. Alina Cernasev, PhD**, Assistant Professor, Department of Clinical Pharmacy and Translational Sciences, College of Pharmacy, The University of Tennessee Health Science Center, Nashville, TN, USA **Title:** Role of pharmacists in directing care in the people living with HIV and substance use disorder **4. Asit Kumar, PhD**, Research Associate, Department of Pharmaceutical Sciences, College of Pharmacy, The University of Tennessee Health Science Center, Memphis, TN, USA **Title:** Effect of smoking constituents on the packaging of CYPs and inflammatory cytokines/chemokines in rat plasma derived extracellular vesicles
9:40 am- 10:05 am	**Coffee/tea break/networking**
10:05 am- 11:10 am	**ECI Symposium** **Co-Chairs:** **Gurudutt Pendyala, PhD**, Robert Lieberman Professor, Department of Anesthesiology, College of Medicine, University of Nebraska Medical Center, Omaha, NE, USA **Yisel Cantres-Rosario, PhD,** Associate Professor, Department of Microbiology and Medical Zoology, University of Puerto Rico, Medical Sciences Campus, PR, USA
	**ECI Pre-Doctoral Students:** **1. Austin Gowen**, Graduate Student, University of Nebraska Medical Center, Omaha, NE, USA **Title:** In utero opioid exposure induced vulnerability to later life brain injury
	**2. Nikhil Kota,** Undergraduate Student, Seton Hall University, South Orange, NJ, USA **Title:** Using IPA tools to characterize molecular pathways underlying the involvement of IRF7 in antiviral response to HIV
	**3. Rachel Podgorski,** Graduate Student, Temple University, Philadelphia, PA, USA **Title:** Chimeric R5 Simian-Human Immunodeficiency Virus SHIV.D replicates in the brain causes neuropathogenesis, and persists on antiretroviral therapy in rhesus macaques **4. Sana Qayum,** Graduate Student, University of Buffalo, NY, USA **Title:** Synthesis and characterization of a PLGA based cannabidiol (CBD) nanoformulation to treat neuropathic pain **5. Zachary Watson,** Graduate Student, University of Texas Medical Branch, Galveston, TX, USA **Title:** Investigating synaptic pruning by glia in HIV associated neurological dysfunction **ECI Post-Doctoral Fellows and Junior Faculty:** **1. Anna Bellizzi, PhD,**Post-Doctoral Fellow, Temple University, Philadelphia, PA, USA **Title:** Discovery and functional characterization of HIV-1-latency associated circular RNAs in African American women living with HIV **2. Subhash Chand, PhD,** Instructor, University of Nebraska Medical Center, Omaha, NE, USA **Titl e:** Methamphetamine induces the release of proadhesive extracellular vesicles and promotes syncytia formation: A potential role in HIV-1 neuropathogenesis **3. Peter Halcrow, PhD,** Post-Doctoral Fellow, University of North Dakota, Fargo, ND, USA **Title:** MU opioid receptor-mediated release of endolysosome iron increases levels of mitochondrial iron, reactive oxygen species, and cell death **4. Huajun Liang, PhD,** Post-Doctoral Fellow, University of Maryland School of Medicine, Baltimore, MD, USA **Title:** Abnormal brain diffusivity in participants with persistent neuropsychiatric symptoms after COVID-19 **5. Kristen McLaurin, PhD,** Post-Doctoral Fellow, University of South Carolina, Charleston, SC, USA **Title:** Disruption of choice: EcoHIV inoculation following a history of cocaine use
11:15 am- 12:15 pm	**ECI speed mentoring** **Co-Chairs:** **Gurudutt Pendyala, PhD**, Robert Lieberman Professor, Department of Anesthesiology, College of Medicine, University of Nebraska Medical Center, Omaha, NE, USA **Yisel Cantres-Rosario, PhD,** Associate Professor, Department of Microbiology and Medical Zoology, University of Puerto Rico, Medical Sciences Campus, PR, USA
12:15 pm- 12:45 pm	**Box lunch (COP Lobby)**
12:45 pm- 1:45 pm	**Business meeting (Dr. Jean M. Bidlack)**
1:45 pm- 2:50 pm	**Symposium 4: HIV and neurological diseases** **Co-Chairs:** **Syed Ali, PhD,** Senior Research Scientist, Center for Integrative Nanotechnology Sciences, University of Arkansas at Little Rock, President & CEO, NeuroLab International, PLLC, Little Rock, AR, USA **RV Srinivas, PhD,** Chief, Extramural Project Review Branch, National Institute on Alcohol Abuse and Alcoholism (NIAAA), National Institute of Health, Bethesda, MD, USA **Speakers:** **1. Golnoush Mirzahosseini, PhD candidate**, Department of Pharmaceutical Sciences, University of Tennessee Health Science Center, Memphis, TN, USA **Title:** LM11A-31 as an anti-stroke and anti-HIV drug candidate **2. Ilker K. Sariyer, PhD,** Associate Professor, Department of Microbiology, Immunology, and Inflammation, Lewis Katz School of Medicine at Temple University, Philadelphia, PA, USA **Title:** Molecular and cellular impact of morphine and HIV-1 NEF-EVs on ORPM1 pre-mRNA splicing **3. Jun Zhu, PhD,** Professor, Department of Drug Discovery & Biomedical Sciences, College of Pharmacy, University of South Carolina, Columbia, SC, USA **Title:** Novel allosteric modulator attenuates HIV-1 tat protein-induced inhibition of dopamine transporter and alleviates cognitive and cocaine rewarding effects in HIV-1 tat transgenic mice **4. Rose Booze, PhD,** Professor and Bicentennial Endowed Chair of Behavioral Neuroscience, Department of Psychology, College of Arts and Sciences, University of South Carolina, Columbia, SC, USA **Title:** Abnormal β-amyloid accumulation: Aging HIV-1 human and HIV-1 transgenic rat brain
2:50 pm- 3:55 pm	**Symposium 5: Selected from the submitted abstracts** **Co-chairs:** **Jerel Fields, PhD**, Assistant Professor, Department of Psychiatry, University of California, San Diego, La Jolla, CA, USA **Susmita Sil, PhD,** Assistant Professor, Department of Pharmacology & Experimental Neuroscience, University of Nebraska Medical Center, Omaha, NE, USA
	**Speakers:** **1. Aditya Bade, PhD,** Instructor, Department of Pharmacology and Experimental Neuroscience, University of Nebraska Medical Center, Omaha, NE, USA **Title:** Dolutegravir inhibition of matrix metalloproteinases: Functional mechanism underlying developmental neuro-abnormalities **2. Keshore R. Bidasee, PhD,** Professor, Department of Pharmacology and Experimental Neuroscience, University of Nebraska Medical Center, Omaha, NE, USA **Title:** Persistent diastolic dysfunction in DTG/TDF/FTC-treated HIV-infected NSG-humanized mice **3. Alejandra Borjabad, PhD,** Assistant Professor, Department of Medicine, Division of Infectious Diseases, Icahn School of Medicine at Mount Sinai, New York, NY, USA **Title:** Potentially beneficial effects of cannabidiol (CBD) in ECO-HIV infection in culture and mice **4. Maxim C-J Cheeran, PhD,** Associate Professor, College of Veterinary Medicine, University of Minnesota, St. Paul, MN, USA **Title:** Traumatic brain injury-induced persistent inflammation is associated with long-term behavioral deficits **5. Debashis Dutta, PhD,** Instructor, Department of Pharmacology and Experimental Neuroscience, College of Medicine, University of Nebraska Medical Center, Omaha, NE, USA **Title:** Methamphetamine potentiation of HIV-1 gp120-associated microglia neuroinflammation via NLRP3 activation **6. Seung Wan Yoo, PhD,** Research Associate, Department of Neurology, Johns Hopkins University School of Medicine, Baltimore, MD, USA **Title:** HIV infection primes the innate immune response to methamphetamine **7. Wei Jiang, MD,** Associate Professor, Department of Microbiology & Immunology Division of Infectious Diseases, Department of Medicine Medical University of South Carolina, Charleston, SC, USA **Title:** CSF anti-CD4 autoantibodies and neuroHIV pathogenesis **8. Marcus Kaul, PhD,** Professor, School of Medicine, Division of Biomedical Sciences, University of California at Riverside, Riverside, CA, USA **Title:** Long-term changes of neurotransmission-related gene expression correlate with impaired behavioral performance caused by methamphetamine and neuroHIV **9. Shamsudheen Moidunny, PhD,** Assistant Professor, Department of Surgery-Oncology Molecular Therapeutics, University of Miami School of Medicine, Miami, FL, USA **Title:** Role of gut microbiome in opioid withdrawal-induced anxiety and depression-like behaviors in HIV-1 transgenic mice **President’s transition, notes of appreciation, and award ceremony (Dr. Sulie L. Chang and Dr. Santosh Kumar)**
4:00 pm- 5:00 pm	**Conclusion**

### SNIP Conference Abstracts

Some abstracts for special presentations, guest speakers, and/or by presenter opt-out are not published in this brief report for SNIP’s 26^th^ Scientific Conference.

## Diversity and Inclusion SNIP Council (DISC) session:

### Monocyte biomarkers define sargramostim treatment outcomes for Parkinson’s disease

Abdelmoaty, MM, PhD ^1^, Machhi, J, PhD^1^, Yeapuri, P, PhD^1^, Shahjin, F, MS^1^, Kumar, V, PhD^2^, Olson, KE, PhD^1^, Mosley, RL, PhD^1^, Gendelman, HE, MD^1^.

*^1^Department of Pharmacology and Experimental Neuroscience, University of Nebraska Medical Center, Omaha, NE68198*.

*^2^Mass Spectrometry and Proteomics Core, University of Nebraska Medical Center, Omaha, NE 68198*.

Dysregulation of innate and adaptive immunity heralds both the development and progression of Parkinson’s disease (PD). Deficits in innate immunity in PD are defined by impairments in monocyte activation, function, and pro inflammatory secretory factors. Each influences disease pathobiology. To define monocyte biomarkers associated with immune transformative therapy for PD, changes in gene and protein expression were evaluated before and during treatment with recombinant human granulocyte-macrophage colony-stimulating factor (GM-CSF, sargramostim, Leukine®). Monocytes were recovered after leukapheresis and isolation by centrifugal elutriation, before and 2 and 6 months after initiation of treatment. Transcriptome and proteome biomarkers were scored against clinical motor functions. Pathway enrichments from single cell-RNA sequencing and proteomic analyses from sargramostim-treated PD patients demonstrate a neuroprotective signature, including, but not limited to, antioxidant, anti-inflammatory, and autophagy genes and proteins (LRRK2, HMOXl, TLR2, TLR8, RELA, ATG7, and GABARAPL2). This monocyte profile provides a novel strategy to track clinical immune-based interventions.

### Brief hydromorphone exposure during pregnancy sufficient to induce maternal and neonatal microbial dysbiosis

Abu, Y, BS ^1^, Tao, J, PhD^1^, Dutta, R, PhD ^1^, Yan, Y, PhD^1^, Vitari, N, BS^1^, Kolli, U, PhD^1^, Roy, S, PhD^1^.

*^1^Department of Surgery, University of Miami Miller School of Medicine, Miami, FL 33136*.

Prenatal opioid exposure is associated with significantly adverse medical, developmental, and behavioral outcomes in offspring, though the underlying mechanisms driving these impairments are still unclear. Accumulating evidence implicates gut microbial dysbiosis as a potential modulator of these adverse effects. However, how opioid exposure during pregnancy alters the maternal and neonatal microbiome remain to be elucidated. Here, we utilize a murine model of brief hydromorphone exposure during pregnancy to examine its impact on the maternal and neonatal microbiome. Fecal samples were collected at various timepoints in dams and offspring to interrogate longitudinal changes in the microbiome. Stomach contents at 2 weeks were also collected as a surrogate for breastmilk and microbial analysis was performed using 165 rRNA sequencing. Alongside alterations in the maternal gut microbial composition, offspring gut microbiota exhibited distinct communities at 2 and 3 weeks. Furthermore, functional profiling of microbial communities revealed significant differences in microbial community-level phenotypes gram-negative, gram-positive, and potentially pathogenic in maternal and/or neonatal hydromorphone exposed groups compared with controls. We also observed differences in stomach microbiota in opioid-exposed vs non-exposed offspring, which suggests breast milk may also play a role in shaping the development of the neonatal gut microbiota. Together, we provide evidence of maternal and neonatal microbial dysbiosis provoked even with brief hydromorphone exposure during pregnancy. Supported by NIH/R01DA050542.

### An integrated systems approach to decode the impact of adolescent nicotine exposure in pre and postnatal oxycodone exposed offspring

Flores, A, BS ^1^, Gowen, A, BS^1^, Schaal, V, BS ^1^, KouI, S, BS^1^, Hernadez, J, BS^1^, Yelamanchili, S, PhD^1^, Pendyala, G, PhD^1^.

*^1^Department of Anesthesiology, University of Nebraska Medical Center, Omaha, NE 68198*.

Gestational and post-gestational exposure to prescription opioids pose a critical public health risk. Recent literature chronicles significant neurodevelopmental and behavioral deficits between offspring exposed to the prescription drug oxycodone (oxy) in utero (IUO) and postnatal (PNO). While previous literature has shown that both pre-and postnatal exposure to oxy impacts neurodevelopment, a notable knowledge gap exists on how exposure to other drugs of abuse during a critical phase of development-adolescence impacts behavioral outcomes in the IUO and PNO offspring. Nicotine widely present in cigarettes represents a ubiquitous clinical interaction that current research does not include. The neurological impact of opioids and nicotine is further complicated when considering the dynamic stressor of withdrawal. This study employed an integrated systems approach to investigate escalating nicotine exposure in adolescence and subsequent nicotine withdrawal in our preclinical oxycodone rat model. Western blot analysis observed alterations of the blood-brain barrier (BBB) and synaptic proteins. RT-qPCR further validated dysfunction of cytokine production in the central nervous system consistent with compromised BBB and neuroimmune dysfunction. Peripheral nicotine metabolism was consistent with increased catabolism of nicotine with respect to PNO & IUO, an indicator of the increased likelihood of addiction. Behavioral assays (hot plate and marble-burying) observed a modest impact of nicotine in general and more specifically withdrawal with respect to nociception. Supported by NIH.

### HIV pre-exposure prophylaxis (PrEP) inhibits oligodendrocyte differentiation

Long, C, BS ^1^, Festa, L, PhD^2^, Grinspan, J, PhD^3^, Jordan-Sciutto, K, PhD^2^.

*^1^Neuroscience Graduate Group, University of Pennsylvania, Philadelphia, PA 19104*.

*^2^Department of Basic and Translational Sciences, School of Dental Medicine, University of Pennsylvania, Philadelphia, PA 19104*.

*^3^Department of Neurology, Children’s Hospital of Philadelphia, Philadelphia, PA 19104*.

Adolescents comprise one-fourth of individuals diagnosed with HIV in the United States each year. White matter abnormalities are a hallmark of HIV-associated neurocognitive disorders (HAND) and transcriptome analysis have found a significant decrease in the expression of genes associated with oligodendrocytes (OL), cells that produce myelin, in the white matter of ART treated, HIV+ individuals. Data from our lab has demonstrated that even in the absence of HIV, select antiretroviral drugs inhibit OL differentiation in vitro. PrEP, a combination of emtricitabine (FTC) and tenofovir disoproxil fumarate (TDF) is an effective method to prevent the transmission of HIV in adolescents at substantial risk for acquiring HIV. As myelination continues through adolescence and into adulthood, it is important to understand the effect of PrEP on developing white matter, which has not been investigated. Using our well established cell culture system for purification and differentiation of primary rat OLs, we demonstrated that treatment with FTC and TDF, alone or in combination, inhibited OL differentiation. Furthermore, treatment with FTC and TDF, alone or in combination, significantly decreased the number of acidic lysosomes and increased the pH of lysosomes in a dose dependent manner. Finally, reacidifying lysosomes restores OL differentiation in the context of FTC and TDF treatment. Our findings suggest that PrEP inhibits OL differentiation and the lysosome may be a therapeutic target to mitigate white matter damage in individuals on PrEP.

### Role of astrocyte-specific NLRP6 inflammasome in methamphetamine-mediated neuroinflammation

Oladapo, A, MS ^1^, Periyasamy, P, PhD^1^, Buch, S, PhD^1^.

*^1^Pharmacology and Experimental Neuroscience, University of Nebraska Medical Center, Omaha, NE 68198*.

Methamphetamine (Meth) is one of the most widely used illicit drugs, with its abuse posing a significant economic and health burden globally. Meth abuse also affects the CNS by activating glial cells and neurons, ultimately contributing to neuroinflammation. Mounting evidence implicates inflammasome proteins as critical mediators regulating cellular activation, thereby impacting altered homeostasis of several crucial biological processes. This study investigated the role of the astrocyte-specific inflammasome, NOD-like receptor family, pyrin domain-containing protein 6 (NLRP6) in Meth-mediated neuroinflammation. Our findings demonstrated dose-dependent upregulation of GFAP (astrocyte activation marker) and NLRP6 protein in both mouse and human primary astrocytes exposed to Meth. There was also a time-dependent upregulation of NLRP6 protein and its signaling mediators including caspase-1, Gasdermin D, and proinflammatory cytokines, Ill and IL18 in mouse and human primary astrocytes exposed to Meth (50 µM). Genetic silencing of NLRP6 using species-specific NLRP6 siRNA further validated the role of NLRP6- inflammasome signaling and cellular activation in Meth-exposed human and mouse primary astrocytes. These in vitro findings were also validated in the frontal cortices and striatum of 6-8 weeks old wild-type mice administered a ramped up dose of Meth for 14 days. Overall, this study underscores a novel role of astrocyte-specific NLRP6 inflammasome signaling in Meth-mediated cellular activation and neuroinflammation. Supported by Nebraska Center for Substance Abuse Research.

### CB2R agonist JWH-133 decreases CATB secretion and neurotoxicity from HIV-infected macrophages by attenuating NF-κB activation, oxidative stress, and lysosomal exocytosis

Rosario-Rodriguez, LJ, PhD ^1^, Gerena, Y, PhD^2^, Cantres-Rosario, Y, MS^3^, Carrasquillo-Carrion, K, MS^4^, Cartagena-Isern, Li, BS^5^, Garcia-Requena, LA, BS^6^, Cuadrado-Ruiz, JC, BS^7^, Rodriguez-De Jesus, AE, MS^3^, Borges-Velez, G, BS^1^, Roche-Lima, A, PhD^4^, Melendez, LM, PhD^1^.

*^1^Department of Microbiology and Medical Zoology, University of Puerto Rico, Medical Sciences Campus, San Juan, PR 00936*.

*^2^Department of Pharmacology, University of Puerto Rico, Medical Sciences Campus, San Juan, PR 00936*.

*^3^Translational Proteomics Center, University of Puerto Rico, Medical Sciences Campus, San Juan, PR00936*.

*^4^Bioinformatics and Health Informatics, University of Puerto Rico, Medical Sciences Campus, San Juan, PR 00936*.

*^5^Department of Chemistry, University of Puerto Rico, Rio Piedras Campus, San Juan, PR 00931*.

*^6^Department of Biology, University of Puerto Rico, Rio Piedras Campus, San Juan, PR 00931*.

*^7^Department of Biology, University of Puerto Rico, Bayamon Campus, Bayamon, PR 00959*.

There are no effective therapies available for HIV-associated neurocognitive disorders (HAND). We have demonstrated increased expression of cathepsin B (CATB), a lysosomal protease, in monocytes, plasma, and postmortem brain tissues of women with HAND. In vitro, increased CATB release from HIV-infected monocyte-derived macrophages (MDM) leads to neurotoxicity. Increased CATB secretion is associated with NF-κB activation, oxidative stress, and lysosomal exocytosis. Cannabinoid receptor type 2 (CB2R) activation decreases NF-KB signaling and oxidative stress. However, it is unknown if CB2R activation affects CATB secretion and neurotoxicity by HIV-infected MDM. We hypothesized that CB2R activation will decrease CATB secretion and neurotoxicity from HIV-infected MDM by attenuating NF-κB activation, oxidative stress, and lysosomal exocytosis. Primary MDM were inoculated with HIV-1ADA and treated with CB2R agonists JWH-133 and HU-308. CATB levels were determined from supernatants using ELISA. Antagonist/Agonist co-administration experiments were performed to determine if CB2R activation was responsible for the effects. Neuronal apoptosis was assessed using a TUNEL assay. MDM lysates were selected for Tandem Mass Tag (TMT) Labeling proteomics. Activation of CB2R with JWH-133 reduced CATB secretion and neurotoxicity from MDM. Proteomics results revealed that JWH-133 decreases CATB secretion and neurotoxicity by downregulating NFκB signaling, oxidative stress, and lysosomal exocytosis from HIV-infected MDM. JWH-133 represents a potential therapeutic ligand against HIV/MDM-induced neurotoxicity and HAND. Supported by NIH F99-NS113455 (LJRR), R25-GM061838 (LJRR), SC1GM11369--01 (LMM), U54GM133807, U54MD007600, P20GM103475, UPR-RCM, UPR-CCC.

### Serpin-derived peptide for the treatment against HIV in the CNS

Soler, YS, BS ^1^.

*^1^Department of Immunology and Nano-Medicine, Florida International University, Miami, FL 33199*.

In the brain, HIV predominantly infects cells such as microglia and astrocytes. These cells form virus reservoirs with low levels of infection that are very hard to eliminate. Despite the use of combined antiretroviral therapy (cART), which increases survival rates in HIV patients, the virus still persists as a chronic condition. The difficulty we still face is that cART is not able to control HIV replication or target inflammation in brain reservoirs, which is believed to be the main cause of HIV-associated neurocognitive disorder, neuronal dysfunction, and pain-related pathology.

Moreover, new studies suggest that some antiretroviral drugs used in HIV patients might be neurotoxic to the CNS. Because of these limitations of cART, the aim of my research is to test a new therapeutic drug (small peptide SP16) for treatment against HIV replication and virus-induced inflammation in brain cells reservoirs. Using an artificial 3D blood brain barrier (BBB) system, we have shown that the SP16 peptide does not affect BBB integrity, but rather the integrity is actually restored when SP16 is added to HIV-infected cells. In addition, our findings have revealed that exposure of HIV-infected CNS cells to SP16 caused a significant decrease in the secretion of the pro-inflammatory molecules, the HIV protein marker (p24), and the transcription factor involved in inflammation (NF-kB); and an increase in Akt expression, which is responsible for cell proliferation and survival. Overall, the anti-viral, anti inflammatory and pro-survival effects of SP16 are mediated by its interaction with LRP1.

### Methamphetamine and the S1 subunits of SARSCoV-2 variant spike proteins dysregulate human brain endothelial cells

Stangis, M, BS ^1^, Adesse, D, PhD^2^, Toborek, M, MD, PhD^1^.

*^1^Department of Biochemistry and Molecular Biology, University of Miami Miller School of Medicine, Miami, FL 33130*.

*^2^Laboratory of Structural Biology, Oswald Cruz Institute, Fiocruz, Rio de Janeiro, FL CEP 21045-900.*.

As the COVID-19 pandemic has continued to evolve, so too has our understanding that this is a whole-body disease, and the question of how the virus is able to influence the brain arose. We have previously shown the effects of SARS-CoV-2 infection and the effects of the S1 subunit of the spike protein alone on human brain endothelial cells, which cause decreases in tight junction proteins in the short term and eventual recovery and overexpression in the long term. In adapting our research to fit the pandemic, we have expanded our experiments to study differences in responses following both exposure to the S1 subunit of the delta strain of COVID-19, and to co-exposure of methamphetamine, as the world’s drug habits have been altered during these trying times. These changes in tight junctions appear to be further amplified in the short-term following co-exposure to Methamphetamine, and release of the inflammatory cytokine IL-6 was also shown to be increased following co-exposure. Preliminary data has also shown that mitochondrial respiration is also impacted in the short-term following both single and co-exposures to the S1 subunit and/or methamphetamine. As we begin to analyze our samples that have been exposed to the delta strain S1 subunit, we have begun to see similarities in the reduction of tight junction expression and will be further studying the previously mentioned areas of mitochondrial respiration and inflammatory cytokine release to map the differences in the severity of the responses of human brain endothelial cells to these different strains. Supported by multiple NIH grants and one each from INOVA Fiocruz and FAPERJ.

## President Symposium: SARS-CoV-2: Comorbidities and therapeutics

### Plenary Lecture: Past, present, and the future of SARS-CoV-2 therapeutics

Jonsson, CB, PhD ^1^.

*^1^Microbiology, Immunology, Biochemistry, UTHSC, Memphis, TN 38163*.

The successful development of several vaccines within less than a year to prevent COVID-19 represents a remarkable achievement, however, as of Spring of 2022, only 14.5% of people living in low-income communities have received one dose of a COVID-19 vaccine. Continued public health mitigation of COVID-19 requires unprecedented, global access to vaccines and therapeutics for all of society and demands additional treatment options for those who may not respond to vaccination. Crucial gaps remain, however, in our toolbox for prevention and treatment. Further, we have knowledge gaps in the underlying mechanisms driving cardiovascular complications, neurological manifestations and long COVID. I will discuss key considerations in mounting much needed drug discovery campaigns to identify new small molecules for treatment of COVID-19 as well as animal models required for drug discovery and development.

### Cerebrovascular pathology of SARS-CoV-2 infection

Toborek, M ^1^, Stangis, M^1^, Torices, S^1^, Adesse, D^1^.

*^1^Department of Biochemistry and Molecular Biology, University of Miami Miller School of Medicine, Miami, FL 33136*.

The CNS is well documented to be a target of SARS-CoV-2 infection, and studies detected SARS-CoV-2 in the brain and the cerebrospinal fluid of COVID-19 patients. The blood-brain barrier (BBB) was suggested to be the major route of SARS-CoV-2 entry into the brain. While neurological and cerebrovascular symptoms play a critical role in so called “long-COVID,” the interactions of SARS-CoV-2 with the brain microvasculature forming the BBB are largely unknown. We hypothesize that infection of brain microvessels by SARS-CoV-2 affects the integrity of the BBB and induces hyper-inflammatory responses via mitochondrial and epigenetic reprogramming. We completed RNA-Seq analyses of transcriptomics signatures of human brain microvascular endothelial cells (HBMEC) infected with SARS-CoV-2. The obtained data allowed us to identify several genes and pathways, which clustered around endothelial activation through NF-kappaB non-canonical pathway. Our work also indicated that following infection with SARS-CoV-2, human brain endothelial cells show decreases in tight junction proteins, accompanied by dysfunction in mitochondrial plasticity. These effects were also observed following exposure to the S1 subunit of the SARS-CoV-2 Spike protein. This down regulation in tight junction proteins further points toward a loss of BBB that may allow for the virus to enter the brain infect cells of the neurovascular unit. Overall, our research provides critically important and therapeutically-relevant information on the involvement of the cerebral microvasculature and the BBB in COVID-19 pathology. Supported by MH128022, MH122235, MH072567, HL126559, DA044579, DA039576, DA040537, DA050528, DA04715, INOVA-Fiocruz and FAPERJ.

### COVID-19 in pediatric immunocompromised hosts

Caniza, M ^1^.

*^1^Miguela Ayala Caniza, St Jude Children’s Research Hospital, Memphis, TN 38105*.

Immunocompromised children are at risk for a more severe outcomes from infections including respiratory viral infections. With the pandemic, to SARS-CoV-2 the impact in at-risk populations, especially in immunocompromised hosts became evident. Studies showed that COVID-19 can produce illness due to over-inflammation and immune-mediated multiorgan injury. However, children with immunosuppression receiving anti-cancer therapy and those with HIV have risks associated with the degree of immunosuppression and other comorbidities. Cancer treatments and other therapeutic treatments should be carefully evaluated and possibly not be interrupted in suspect or confirmed SARS-CoV-2 infection. Supported by St. Jude Children’s Research Hospital.

### SARS-CoV-2: Neuropsychiatric complications

Letendre, SL, MD ^1^.

*^1^Departments of Medicine and Psychiatry, University of California, San Diego, San Diego, CA 92103*.

SARS-CoV-2 has caused a tragic pandemic with more than 6 million deaths worldwide. While new vaccines and therapies have greatly improved disease outcomes, nearly 500 million people have been infected and are at risk for post-acute sequelae of COVID-19 (PASC). Neuropsychiatric complications of the infection are common and range from the mild (e.g., hyposmia) to the severe and life-threatening (e.g., encephalitis). Those who have more severe COVID-19 are at greater risk for these complications. The pathogenesis appears to be due to a combination of inflammation, thrombosis, hypoxia, and in some people, autoimmunity. While the acute effects of infection can be devastating, many people are affected by PASC, also called long COVID-19. Neuropsychiatric symptoms figure prominently in PASC and include cognitive and mood symptoms, insomnia, dysautonomia, chronic pain, and fatigue. These symptoms may result from brain injury suffered during the acute infection as well as a persistent inflammatory and prothrombotic state. COVID-19 could also unmask vulnerability to other conditions, such as Alzheimer’s Disease. While antiviral and anti-inflammatory drugs improve symptoms and survival in acute infection, no randomized clinical trials have yet supported safe and effective therapies for neuroPASC. While the severity and scale of COVID-19 have been tragic, the pandemic presents opportunities to better understand the biological mechanisms by which infections such as SARS-CoV-2 affect neuropsychiatric health and to develop new therapies that will benefit people with neuroPASC. Supported by National Institutes of Health.

## Symposium 1: Use of iPSC-derived CNS cells and brain organoids to model HIV infection, substance use and ARV treatment in CNS

### iPSC-based platforms reveal new opportunities for modeling HIV infection in the CNS

Akay-Espinoza, C, MD ^1^, Gesualdi, J^1^, Starr, A^1^, Nickoloff-Bybel, E, PhD^1^, Jordan-Sciutto, K, PhD^1^.

*^1^Department of Oral Medicine, University of Pennsylvania, School of Dental Medicine, Philadelphia, PA 19104*.

Despite vastly improved outcomes with antiretroviral therapy (ART), up to 50% of people with HIV infection (PWH) continue to experience neurocognitive (NC) impairment (NCI) and other neurological disorders. In the setting of ART, progression to more severe NC disorders is more likely in PWH with mild NCI than in unimpaired PWH. Studies on HIV infection in the CNS have been mainly limited to laboratory animal and postmortem studies, and freshly isolated primary cells nonetheless rapidly lose key in vivo features in vitro. The development of effective therapeutic approaches addressing persistent NCI in PWH on ART requires models closely recapitulating the key characteristics of specialized cell populations. Induced pluripotent stem cell (iPSC)-based models provide new opportunities for in vitro mechanistic studies of interactions among neurons, microglia, and astrocytes during HIV infection in the correct CNS context. We herein present evidence that models based on IPSC-derived neurons, astrocytes, and microglia can be utilized to i) dissect the replication dynamics of HIV infection, ii) understand the direct and indirect effects of HIV on specific cell types, iii) assess the impact of ART on cellular outcomes, and iv) interrogate comorbidities such as drugs of abuse in the context of HIV and ART. These models provide an important new platform for the interrogation of cellular, molecular, and genetic drivers of NCI in PWH as well as the development of novel therapeutic approaches in a system that more closely recapitulates the key determinants of HIV infection in the CNS. Supported by NIH, R01-DA052826; NIH, R44-MH119621.

### Evaluating the impact of antiretroviral drugs in a forebrain organoid model of cortical development

Christian, KM, PhD ^1^, LaNoce, E, BS^1^, Dumeng-Rodriguez, J, BS^1^, Garcia, A, BS^1^, Zhang, D, BS^1^, Song, H, PhD^1^, Ming, GL, MD, PhD^1^.

*^1^Department of Neuroscience, University of Pennsylvania, Philadelphia, PA 19104*.

Historically, pregnant populations have often been excluded from clinical trials and there are limited data on the impact of many therapeutic drugs on fetal brain development. Human induced pluripotent stem cells (iPSCs) offer new opportunities to investigate the safety of drugs on human neural cell types. This may be particularly important to help guide clinical decisions during pregnancy if there are multiple drugs that are considered effective. Human iPSCs can be cultured under conditions that allow for the self-organization of neural progenitors into organoids that model several features of the developing cortical forebrain. Based on results from an observational study in Botswana suggesting that the antiretroviral drug dolutegravir was associated with a slightly increased risk of neural tube defects if taken at the time of conception, we evaluated the impact of dolutegravir on early-stage cortical organoids. Our results thus far suggest that sustained exposure to dolutegravir, but not raltegravir, impacts organoid structure and organization and leads to a deficit in neurogenesis. RNA sequencing revealed an upregulation of stress-associated genes and a downregulation of genes related to neurogenesis. This study is a proof-of-principle to demonstrate the utility of iPSC-based models to generate complementary data that can be used to evaluate the impact of drugs on developing human neurons. Supported by NIH/NIDA (5R01DA049514).

### AUD-associated, noncoding SNP variants of KCNJ6 alter physiology in iPSC-derived neurons

Hart, RP, PhD ^1^, Popova, D, PhD^1^, Gameiro-Ros, I, PhD^2^, Pang, ZP, MD^3^, Slesinger, PA, PhD^2^.

*^1^Department of Cell Biology & Neuroscience, Rutgers University, Piscataway, NJ 08854*.

*^2^Department of Neuroscience, Icahn School of Medicine at Mt. Sinai, New York, NY 10029*.

*^3^Child Health Institute, RWJ School of Medicine, Rutgers University, New Brunswick, NJ 08854*.

Synonymous and noncoding single nucleotide polymorphisms (SNPs) in the KCNJ6 gene, encoding G protein-gated inwardly rectifying potassium (GIRK2) channel subunit 2, have been linked with increased electroencephalographic frontal theta event-related oscillations (ERO) in subjects diagnosed with alcohol use disorder (AUD). To identify molecular and cellular mechanisms in a human model, we generated excitatory glutamatergic neurons (iN) from iPSCs derived from four AUD-diagnosed subjects with KCNJ6 variants and four control subjects. Neurons were analyzed for changes in gene expression, morphology, calcium imaging and physiological properties. Single cell RNA sequencing predicts that KCNJ6 major allele neurons have altered patterns of synaptic transmission and cell projection morphogenesis, with minor allele neurons expressing lower levels of GIRK2, greater neurite area, and elevated excitability. Interestingly, exposure to intoxicating concentrations of ethanol induces GIRK2 expression and reverses functional effects in minor allele neurons. Ectopic overexpression of GIRK2 alone mimics the effect of ethanol to normalize induced excitability. We conclude that KCNJ6 variants decrease GIRK2 expression and reduce excitability and that this effect can be minimized or reversed with alcohol usage. Supported by NIAAA.

### HIV infection of human iPSC-derived microglia-containing cerebral organoids

Bodnar, B, BS ^1^, Wang, P, PhD^1^, Liu, J, PhD^1^, Zhang, D^5^, Wang, X, PhD^1^, Meng, F^1^, Wei, Z, PhD^1^, Fan, Y^1^, Li, Q, PhD^4^, Hu, WH, MD, PhD^1^, Ho, WZ, MD, MPh^1^.

*^1^Department of Pathology and Laboratory Medicine, Lewis Katz School of Medicine at Temple University, Philadelphia, PA 19140*.

*^2^Center for Metabolic Disease Research, Lewis Katz School of Medicine at Temple University, Philadelphia, PA 19140*.

*^3^Center for Substance Abuse Research, Lewis Katz School of Medicine at Temple University, Philadelphia, PA 19140*.

*^4^Nebraska Center for Virology, School of Biological Sciences, University of Nebraska-Lincoln, Lincoln, NE 68583*.

*^5^Smilow Center for Translational Research, University of Pennsylvania, Philadelphia, PA 19104.*.

Studying the mechanisms of HIV-associated neurocognitive disorders (HAND) requires an appropriate in vitro brain model. In this study, we developed human induced pluripotent stem cell (hiPSC)-derived microglia-containing cerebral organoids (MCOs) which contain not only astrocytes, neurons, and neural stem/progenitor cells, but also microglial cells, the primary target of HIV infection in the CNS. We demonstrated that MCOs expressed both the HIV major entry receptor (CD4) and coreceptors (CXCR4 and CCR5) and could be productively infected by HIV. Infected MCOs showed a steady and significant increase of HIV gag mRNA and p24 protein expression, followed by latent infection with only detectable gag DNA and little expression of the gag mRNA and p24 protein. HIV RNA could bedetected in infected MCOs by RNAscope in situ hybridization using antisense probes of the virus. Addition of HIV replication stimulants (TNFa, PMA, and SAHA) to latently infected MCO cultures could reactivate viral replication evidenced by elevated expression of HIV gag mRNA and p24 protein. Existence of replication-competent intact HIV provirus in reactivated MCOs was also demonstrated by viral outgrowth assays using HutR5 cells. Single-cell RNA sequencing analysis of HIV-infected MCOs revealed that microglia is the major target of the virus. These observations suggest that MCOs be an alternative and suitable in vitro brain model for studying both acute and latent HIV infection of microglia in the presence of other major brain cells, which is crucial to examine mechanisms underlying HAND immunopathogenesis.

## Bill Narayan Memorial Lecture:

### Alcohol and electronic cigarettes cause blood brain barrier (BBB) injury via purinergic receptor signaling

Persidsky, Y, MD, PhD ^1^, Mekala, M, PhD^1^, Gajghate, S, MS^1^, Rom, S, PhD^1^, Reichenbach, N, BS^1^.

*^1^Dept. Pathology/Laboratory Medicine, Temple University School of Medicine, Philadelphia, PA 19040*.

We have demonstrated BBB compromise (increase permeability, diminished expression of tight junction proteins/ glucose transporter) in animal models of chronic alcohol (EtOH) and e-Cig exposure (2 months). Current study explored potential molecular mechanisms associated with these effects. Using human brain endothelial cells (BMVEC), we showed that exposure to EtOH, acetaldehyde (Ace) or e-Cig resulted in mitochondrial dysfunction (decrease by 30-50% of spare respiration capacity). These changes paralleled diminution of mitochondrial OXPHOS markers including Complex- II, III and V. EtOH, Ace and e-Cig increased the intracellular Ca2+accumulation associated with changes in cytoskeleton contraction/diminished barrier function. Treatment with e-Cig conditioned media or EtOH/Ace also led to enhanced expression of markers of endoplasmic reticulum stress. As mitochondrial dysfunction can be accompanied by ATP release, we measured extracellular concentrations of ATP that acts as important cell signaling mediator. In response to EtOH, Ace and e-Cig treatments, BMVEC demonstrated 4- to 7- fold increase in extracellular ATP levels. We assessed expression of purinergic receptors and documented increased expression of the P2X7r and TRPV1 channels after exposure to EtOH, Ace and e-Cig indicating potential increase in receptor signaling. P2X7r inhibition normalized the expression of mitochondrial OXPHOS markers, mitochondrial function, diminished Ca2+accumulation and ATP release. These data indicate that purinergic signaling is involved in BBB injury due effects of alcohol or e-Cig. Supported by NIAAA, NIMH.

## Dr. Adrash Kumar Lecture:

### Multifaceted roles of exosomes in neuroHIV & opioid comorbidity

Buch, S, PhD ^1^, Sil, S, PhD^1^, Kannan, M, PhD^1^, Chemparathy, D, PhD^1^, Singh, S, PhD^1^, Liao, K, PhD^2^.

*^1^Department of Pharmacology and Experimental Science, University of Nebraska Medical Center, Omaha, NE 68198*.

*^2^Smidt Heart Institute, Cedars-Sinai Medical Center, Los Angeles, CA 90048*.

Neuronal damage and neuroinflammation are the hallmark features of Neuro-HIV. Several studies have implicated opioid abuse as a factor in the accelerated incidence and progression of NeuroHIV; the molecular mechanism(s) underlying this potentiation of neuropathogenesis, however, remains elusive. One of the mechanisms gaining popularity in the field, is the cellular crosstalk involving extracellular vesicles (EVs), that act as conduits for progression HIV & opioid-mediated synaptodendritic injury and neuroinflammation. Our previous findings have demonstrated multiferous roles of HIV Tat and Morphine-stimulated astrocyte derived-EVs (ADEVs) in mediating neuronal and microglial dysfunction. For example, it has been shown that Tat-ADEVs carrying proteins (amyloids as well as senescence markers) and miRs (miR7), can result in neuronal synaptodendritic. Since opioid abuse is known to go hand-in-hand with NeuroHIV, we also assessed the role of Mor-ADEVs. It was shown that miR-138 carried by the Mor-ADEVs can activate microglia by direct binding of the GUUGUGU motif of miR138 to the endosomal toll-like receptor (TLR)7. In addition to microglial activation, Mor-ADEVs can also impair microglial phagocytosis involving the TLR7-NF-kB -lincRNA-Cox2 axis. Notably, these findings formed the basis of assessing the therapeutic potential of delivering lincRNA-Cox2 siRNA intranasally, as a means to ameliorate morphine-mediated microglial activation. Furthermore, Mor can also mediate induction & release of miR-23a in ADEVs, which, upon uptake by the pericytes, leads to pericyte migration. Supported by NIDA.

## Symposium 2: T time for neuroimmune pharmacology research

### Loss of forebrain cholinergic neurons in aged HIV-1 gp120 transgenic rodents is mediated through the p75NTR neurotrophin receptor

Speidell, A, MS ^1^, Mocchetti, M, PhD^1^.

*^1^Department of Neuroscience, Georgetown University Medical Center, Washington, DC 20057*.

Human immunodeficiency virus type 1 (HIV) positive individuals exhibit a constellation of neurological symptoms, termed HIV-associated neurocognitive disorders (HAND). These symptoms affect nearly one in two people living with HIV (PLH), even in the post-combined antiretroviral therapy (cART) era. The continued production and release of viral proteins in the CNS is hypothesized to contribute to HAND neuropathogenesis. Our lab has shown that the HIV envelope protein gp120 alters neuronal proconvertase expression, increasing proneurotrophin abundance in an age-dependent manner and activating the pro-apoptotic proneurotrophin receptor p75NTR. We hypothesized that basal forebrain cholinergic neurons (BFCNs), which highly express p75NTR, are uniquely susceptible to gp120-driven neuronal apoptosis through this receptor. To this end, we crossbred gp120 transgenic (tg) and p75NTR-/- mouse colonies to obtain 3- and 12-month -old (mo) cohorts of wild type (wt), gp120tg, p75NTR+/- and p75NTR+/-gp120tg mice. Our data suggest that only aged gp120tg mice are impaired on a task of extinction of conditioned fear, a complex cognitive behavior subserved by BFCN innervation of the hippocampus and prefrontal cortex. Imaging endpoints obtained from BFCNs in our experimental groups also indicate a decrease in neurite complexity and suggest a reduction in the population of choline acetyltransferase-positive cells in the forebrain of 12mo+ gp120tg animals. Our results show that loss of BFCNs in HAND individuals may underpin the observed collection of neurological impairments in PLH. Supported by NINDS R01 079172, T32 041218, and F31 124490.

### Effect of methamphetamine on establishment and reactivation of HIV-1 latency in myeloid cell lines and iPSC-derived microglia

Pathak, R^1^, Mandarino, A, PhD ^2^, La Porte, A^1^, Dixit, U^1^, Prasad, VR, PhD^2^, Kalpana, GV, PhD^1^.

*^1^Department of Genetics, Albert Einstein College of Medicine, Bronx, NY 10461*.

*^2^ Department of Microbiology and Immunology, Albert Einstein College of Medicine, Bronx, NY 10461*.

Understanding the nature and mechanism of CNS HIV-1 latency is a key goal in mitigating HAND. To characterize transcriptional/post-transcriptional blocks in latency and to quantitate reactivation kinetics, we established a Single-cell Single Molecule multiplex IF and RNA-FISH based Assay (SMIRA). Using SMIRA, we employed latently infected myeloid cell lines to study kinetics of latency reactivation by LRAs and the effect of Meth. First, we studied U1 monocytic latency cell line model using PMA and TNF-α. HIV reactivation was slow, forming two transcription sites (TS) as early as 12h consistent with the presence of two integrated proviruses. Number of cells with TS reached a peak at 30h, but only a small percentage were activated. Methamphetamine reactivated very few U1 cells, but combined with PMA, it had an additive effect. We also tested the effect of Meth on HIV latency in two other cell line models of latency (THP89GFP and OM10.1) each containing a single integrated HIV-1 provirus. Our results are similar to those observed by SMIRA with U1, in that Meth alone has small effects, but it accentuates the reactivation observed by PMA. To study these questions in primary CNS cell lines, we have established iPSC-derived microglial cells. We confirmed that these cells are CD11b+ and CD45- by immunohistochemistry. We are currently infecting them with HIV-1ADA and monitoring the infection for several days using RNA-FISH and IF and we will be studying the effect of Meth on both establishment and reactivation of latency in these cells. Supported by NIH R01 DA043169 (MPIs: Dr. G. Kalpana and V. Prasad) and NIH T32 AI007501 (PI: V. Prasad).

### Alterations in peripheral nociceptive signaling circuits during HIV/SIV infection contribute to the development of HIV-associated distal sensory polyneuropathy

Warfield, R, BS ^1^, Robinson, JA, BS^1^, Smith, MD, MS^1^, Baak, S^1^, Burdo, TH, PhD^1^.

*^1^Department of Microbiology, Immunology and inflammation, Temple University Lewis Katz School of Medicine, Philadelphia, PA 19140*.

HIV-associated distal sensory polyneuropathy (HIV-DSP) has remained prevalent in the antiretroviral (ART) era. Using SIV-infected non-human primates (NHPs), we have identified atrophy in nociceptive neurons that is associated with increased inflammatory monocyte traffic to the dorsal root ganglia (DRG) during SIV infection. Levels of pro-inflammatory cytokines, IL-6 and IL-1β, are elevated in cerebral spinal fluid and plasma with SIV infection and are not significantly decreased in ART-treated NHPs. We have identified a significant increase in expression of nociceptive ion channels; transient receptor potential vanilloid (TRPV1), ankyrin (TRPA1), and the precursor form of brain derived neurotrophic factor (BDNF), proBDNF, in the DRGs of SIV-infected ART-treated NHPs compared to SIV-infected ART-naïve NHPs. We hypothesize that residual neuroinflammation despite ART drives alterations in nociceptive ion channel expression and BDNF leading to increased peripheral sensitization. To determine the underlying mechanism, we have developed and characterized human iPSC-derived peripheral sensory neurons (IPSC-PSNs). IPSC-PSNs treated with plasma-relevant concentrations of the ART regimen used in NHPs showed no significant upregulation of TRPV1/TRPA1 or BDNF. This indicates that ART itself may not directly contribute to increased expression of nociceptive ion channels and neurotrophins observed in NHPs. Ongoing studies are needed to determine the role of proinflammatory cytokines and immune cells in SIV/HIV-associated nociceptor sensitization. Supported by Temple Interdisciplinary and Translational NeuroAIDS Research Training Program.

### Role of exosomes in neuroinflammation caused by retroviral infection

Joseph, J, MS ^1^, Pinto, D, PhD ^2^, Rao, A, MS^1^, Carey, A, MD^3^, Stoffel, V, BS^1^, Bergmann-Leitner, E, PhD^2^, Jain, P, PhD^1^.

*^1^Department of Microbiology & Immunology, Drexel University College of Medicine, Philadelphia, PA 19129*.

*^2^Center of Infectious Disease Research, Walter Reed Army Institute of Research, Silver Springs, MD 20910*.

*^3^Department of Pediatrics, Drexel University College of Medicine, Philadelphia, PA 19102*.

Infection with HTLV-1 can lead to a chronic, progressive demyelinating condition in the spinal cord, a neuroinflammatory disease –HAM/TSP. Over the years, our laboratory has studied the quality of the anti-viral immunity in HAM/TSP patients and established a negative correlation between the aberrant activity of immune checkpoint (ICP) pathways and cytolytic potential of T cells. In recent years, ICPs have been shown to be secreted in a soluble form and carried on the surface of extracellular vesicles (EVs) including exosomes, with immunomodulatory activity. Consequently, we characterized these vesicles from HTLV-1 infected cell lines, including one representing HAM/TSP. Comparative analyses of membranous and soluble ICPs, solidified our previous observations with PD-1: PD-L1/L2 pathway but also revealed a distinct presence of a unique negative checkpoint receptor, BTLA and its ligand HVEM. This was also seen elevated in the sera of HAM/TSP patients. BTLA on exosomes was able to interact with its ligand in a modified ELISA assay validating its receptor function. HTLV-1 infection, more so its b-Zip protein , HBZ, has been linked to enhance sEV release via BDNF/Trkb and could serve as a causative mechanism for our current observations. Indeed, we saw that treatment with anti-retroviral drugs significantly reduced ICP levels and HBZ expression in cell systems and is being further evaluated in HBZ knockdown systems. Evaluating the effects of BTLA/HVEM blockade in restoring T-cell functions in HAM patients can assist in future immunotherapeutic strategies. Supported by NIH 1R01NS0971-47.

## Symposium 3: Meet the *Neuroimmune Pharmacology and Therapeutics* (*NIPT*) Editors

### Strategies for an HIV cure

Gendelman, HE, MD ^1^, Kevadiya, B, PhD^1^, Hasan, M, BS^1^, Edagwa, B, PhD^1^.

*^1^Pharmacology and Experimental Neuroscience, University of Nebraska Medical Center, Omaha, NE 68154*.

We developed the means to track, deliver, distribute, suppress and excise integrated proviral DNA by long-acting slow effective release antiretroviral and CRISPR-Cas9 excision therapies. These are delivered to “putative” viral reservoirs. Our research goals are to provide rapid and effective delivery of antiretroviral drug formulations and CRISPR guide RNAs to maximize therapeutic responses. Our science involves pharmacokinetic and pharmacodynamic (PK and PD) screening and viral excision. The work builds on prior success in cell-based and humanized mouse models. Supported by 5R01MH115860.

### The current status of the viral reservoirs within the CNS*.*

Donoso, M^1^, D’Amico, D^1^, Valdebenito, S^1^, Hernandez, C^1^, Prideaux, B^1^, Eugenin, EA ^1^.

*^1^ Department of Neuroscience, Cell Biology, and Anatomy, University of Texas Medical Branch (UTMB), Galveston, Texas, USA*.

The major barrier to cure HIV infection is the early generation and extended survival of HIV reservoirs in the circulation and several tissues. Currently, the techniques used to detect and quantify HIV reservoirs are mostly based on blood-based assays; however, it has become evident that viral reservoirs remain in several tissues.

Our manuscript describes a novel imaging multi-component method (HIV DNA, mRNA, and viral proteins in the same assay) to identify, quantify, and characterize viral reservoirs in tissues and blood products obtained from HIV-infected individuals even when systemic replication is undetectable. In the human brains of HIV-infected individuals under ART, we identified that microglia/macrophages and a small population of astrocytes are the main cells with HIV-integrated DNA. Only half of the cells with HIV-integrated DNA expressed viral mRNA, and one-third expressed viral proteins. Surprisingly, we identified residual HIV-p24, gp120, nef, vpr and tat protein expression and accumulation in uninfected cells around HIV-infected cells suggesting local synthesis, secretion, and bystander uptake. In conclusion, our data show that ART reduces the size of the brain HIV reservoirs; however, local/chronic viral protein secretion still occurs, indicating that the brain is still a major anatomical target to cure HIV infection.

Funding: This work was funded by The National Institute of Mental Health grant, MH128082, the National Institute of Neurological Disorders and Stroke, NS105584, and UTMB internal Texas funding to E.A.E.

### Brain abnormalities in patients with post-acute sequelae of COVID-19 (PASC)

Chang, L, MD ^1^, Liang, HJ, MD, PhD^1^, Wilson, E, MD^2^, Ryan, M, MS^1^, Oishi, K, MD, PhD^3^, Cunningham, E, BS^1^, Zhang, X, MS^1^, Kottilil, S, MD, PhD^2^, Ernst, T, PhD^1^.

*^1^Departments of Diagnostic Radiology and Nuclear Medicine, and Neurology, University of Maryland School of Medicine, Baltimore, MD 21201*.

*^2^Institute of Human Virology, University of Maryland School of Medicine, Baltimore, MD 21201*.

*^3^Department of Radiological Sciences, Johns Hopkins University School of Medicine, Baltimore, MD 21278*.

Background: Between 30-80% of individuals recovered from SARV-CoV2 infection suffer from post-acute sequelae of COVID-19 (PASC). Patients with PASC often have memory complaints and neuropsychiatric symptoms. How the brain may be impacted in PASC remains unclear. Methods 57 participants were evaluated: 30 PASC [10 men/20 women, 21-63 years old, 219±134 days since infection] and 27 SARS-CoV-2 negative controls [11 men/16 women, 25-68 years old). Each completed the NIH Toolbox® and PROMIS to evaluate cognition, motor function and emotions. Brain imaging studies, with diffusion tensor imaging (DTI), blood-oxygenation level dependent-functional MRI (BOLD-fMRI) and MR spectroscopy were also performed. Results Compared with controls, PASC had normal cognitive performance but elevated levels of psychiatric symptoms (anxiety, depression, fear, sadness, anger, and stress). However, on DTI, they showed more restricted diffusivity in multiple white matter tracts, and higher diffusivity in the amygdala in women. On BOLD-fMRI, PASC showed greater activation in the working memory network than controls. On MR spectroscopy, PASC had lower levels of neuronal markers (N-acetylaspartate and glutamate) and glial metabolite (myoinositol), as well as lower glutathione levels (especially in men) in the frontal regions compared to controls. Conclusions: The abnormalities on various brain imaging measures, despite normal cognitive performance, provide evidence for subclinical brain injury in PASC. Longitudinal follow-ups are needed to determine whether these brain abnormalities will normalize. Supported by R21NS121615.

### Stop paying tolls in the CNS for halting neurodegenerative diseases

Pahan, K, PhD ^1^.

*^1^Neurological Sciences, Rush University Medical Center, Chicago, IL 60612*.

Toll-like receptor (TLR) serves as an important link between innate and adaptive immune responses. Although TLRs respond mainly to bacteria, bacterial products, virus, and flagellin by transmitting a ligand-induced transmembrane signal, recently it is seen that aggregated proteins in the CNS activate TLR2. Induction of TLR2 activation depends on its association with the adapter protein MyD88. Accordingly, we have found that TLR2 and MyD88 levels are elevated in the CNS of patients with Alzheimer’s disease (AD) and Parkinson’s disease (PD). Since there is no specific inhibitor of TLR2, to target induced TLR2 from a therapeutic angle, we designed a peptide corresponding to the TLR2-interacting domain of MyD88 (TIDM) that binds to the BB loop of only TLR2, and not other TLRs. Interestingly, wtTIDM peptide inhibited microglial activation induced by fibrillar Aβ1-42 and alpha-syn, but not 1-methyl-4-phenylpyridinium, dsRNA, bacterial lipopolysaccharide, flagellin, or CpG DNA. After intranasal administration, wtTIDM reached the hippocampus, reduced hippocampal glial activation, lowered Aβ burden, attenuated neuronal apoptosis, and improved memory and learning in 5XFAD mice. However, wtTIDM was not effective in 5XFAD mice lacking TLR2. Similarly, in PFF-seeded A53T mice, nasal wtTIDM or genetic deletion of TLR2 reduces also glial inflammation, decreases α-syn spreading, and protects dopaminergic neurons. Therefore, selective targeting of TLR2 by wtTIDM may be beneficial for AD, PD and other neurological disorders in which TLR2/MyD88 signaling plays a role in disease pathogenesis. Supported by NIH grants (AG050431, AG069229 and NS108025).

## Local Symposium:

### Plenary Lecture: Ionic mechanisms of alcohol-induced brain hypoperfusion – focus on BK channels

Dopico, A, PhD ^1^.

*^1^Department of Pharmacology, Addiction Science, and Toxicology, University of Tennessee Health Science Center, Memphis, TN 38163.*.

In the present talk I will review the literature, mainly from my laboratory, dealing with the modulation of potassium channels of the BK type by concentrations of alcohol (ethanol) reached in blood that constitute legal intoxication in most states of the US (0.08 g/dL=17.4 mM), and the consequences of such modulation on organ physiology and behavior. From a historical perspective, the focus of the talk will go from the supraoptic system to striatal neurons to cerebral arteries. In all cases, modifications in cell physiology by ethanol exposure within each alcohol-naïve system will be analyzed in terms of the different molecular entities that determine the final response of BK channels to ethanol, including the identification of an alcohol-sensing site in BK channel-forming α subunits (i.e., slo1 channels) and the influence of different regulatory BK β subunits on the final response of a given system to alcohol exposure. The possibility that an ethanol-recognition site(s) in the BK channel plays a role in the neurophysiological and behavior responses evoked by ethanol in a rodent model of alcohol-induced blackout will be discussed. Lastly, the participation of the different elements that determine the tissue/organismal response to protracted ethanol exposure will be briefly presented, with insights obtained from invertebrate and vertebrate models. Supported by NIH R37-AA11560.

### Kappa opioid receptor modulation of nucleus accumbens microcircuits

Grueter, B, PhD ^1^, Coleman, BC, PhD^1^, Manz, KM, MD, PhD^1^.

*^1^Anesthesiology, Vanderbilt University Medical Center, Nashville, TN 37232*.

The dynorphin/kappa opioid receptor (KOR) system within the nucleus accumbens (NAc) contributes to negative affective states following withdrawal from illicit drugs, pain, and stress. In the NAc, parvalbumin fast-spiking interneurons (PV-FSIs) receive similar excitatory input as neighboring medium spiny neurons (MSNs) and inhibit MSN activity via feedforward inhibition, critically regulating NAc circuit dynamics. KORs presynaptically inhibit glutamate release onto MSNs, but dynorphin/KOR regulation of excitatory drive onto PV-FSIs remains unknown. Thus, we characterize KORs at glutamatergic synapses onto NAc PV-FSIs. Using PV-cre/Ai9(tdTom) mice and whole cell electrophysiology, we show that activation of KORs induces long-term depression (LTD) of excitatory drive onto PV-FSIs that is mediated by PKA and calcium/calcineurin dependent endocytosis of AMPA receptors. We also report that KORs preferentially modulate midline nuclei of the thalamus afferents and not prefrontal cortex afferents onto NAcc PV-FSIs. To determine the potential involvement of this mechanism in the response to stress, mice were subjected to restraint stress for one hour, a manipulation shown to increase NAc dynorphin. In stressed animals the KOR agonist no longer elicited LTD. Interestingly, the LTD is rescued by administration of the KOR antagonist, nor-BNI, prior to the stress. This work provides a novel mechanism and synaptic locus by which KORs modulate NAc circuit activity and provides evidence for the recruitment and/or dysregulation of this mechanism following stress. Supported by NIDA/2R01DA040630-06A1.

### Molecular interactions between drug abuse and HIV-1 neuropathogenesis

Pandhare, JP, PhD ^1^.

*^1^Center for AIDS Health Disparities Research, Meharry Medical College, Nashville, TN 37208*.

Cocaine, a commonly abused drug among HIV patients, has been associated with accelerated HIV disease and AIDS-related mortality, even among ART adherent patients. In addition, HIV-1 infection in the brain leads to a range of neurological dysfunctions that are broadly termed as HIV-1 associated neurological disorders (HAND). Cocaine is a highly addictive psychostimulant drug that has been linked to increased risk of developing HIV-1 neuropathogenesis. Several lines of evidence suggest that cocaine affects HAND pathogenesis by increasing viral load and inducing neuronal damage via oxidative stress leading to apoptosis as well as autophagy. We have reported a novel mechanism illustrating that cocaine exposure mediates modulation of proline metabolism to trigger neuronal autophagy and its implication in HAND. HIV-1 and cocaine are also known to induce effects via alterations in micro RNA (miRNA) expression and subsequent post-transcriptional regulation of gene expression. We have demonstrated that cocaine-induced downregulation of miR-125b in primary CD4+ T cells results in increased viral protein production. Furthermore, our studies have identified the mechanism by which miRNAs- miRNA-125b/ miR-124 that are cocaine regulated miRNAs mediate posttranscriptional regulation of PolyADP-ribose polymerase-1 (PARP-1). This cocaine mediated PARP-1 regulation is responsible for alterations in gene expression that contribute to the reward mechanisms of cocaine. Collectively, our studies provide fundamental new insights into the role of miRNAs in HIV-1 and cocaine induced effects. Supported by NIDA, NIMHD.

### Role of pharmacists in directing care in the people living with HIV and substance use disorder

Cernasev, A ^1^, Kumar, S, PhD^1^.

*^1^University of Tennessee Health Science Center, College of Pharmacy, Nashville, TN 37211*.

Background: The U.S. opioid epidemic continues to have a significant, negative impact on public health. Although the number of opioid prescriptions has decreased nationwide, Tennessee continues to rank third in prescribing in the U.S. Considering Americans live in close proximity to a community pharmacy, pharmacists are well positioned to interact and direct care for Persons Living With HIV (PLWH). Methods: Interviews were used as a qualitative approach for this study. Recruitment of PLWH occurred via fliers in Tennessee and continued until saturation was achieved. All interviews were audio recorded and transcribed verbatim by a professional transcription service. Thematic Analysis was performed by one researcher using Dedoose, a qualitative software to extract themes. Results: Sixteen interviews were conducted between December 2019 and August 2020. Each interview was approximately 40-120 minutes in length. The majority of subjects identified themselves as men (n=13) and African American (n=14). Two subjects stated that they were illiterate (i.e., could not write or read). Thematic analysis showed that ‘lived experiences’ were associated with (1) taking antiretroviral treatment to stay “alive” and (2) encounters with the pharmacists that helped to develop a trustful relationship. Conclusions: Three critical key aspects emerged for this patient population: (1) achieving clinical goals, (2) navigating the health care system, (3) more detailed counseling session when they receive opioid medications could be crucial. Community pharmacists can apply these findings for progress.

### Effect of smoking constituents on the packaging of CYPs and inflammatory cytokines/chemokines in rat plasma derived extracellular vesicles

Kumar, A, PhD ^1^, Sinha, N, BS^1^, Haque, S, PhD^1^, Kodidela, S, PhD^1^, Wang, T, PhD^2^, Martinez, AG, PhD^2^, Chen, H, PhD^2^, Kumar, S, PhD^1^.

*^1^Department of Pharmaceutical Sciences, University of Tennessee Health Science Center, Memphis, TN 38163*.

*^2^Department of Pharmacology, University of Tennessee Health Science Center, Memphis, TN 38163*.

In this study, we aimed to investigate the effect of nicotine self-administration (SA-NIC) in response to menthol and audiovisual (AV) cue on packaging of CYP2A6 and inflammatory and oxidative stress modulators in rat plasma-EVs. We also studied the interaction between benzo(a)pyrene (BaP) and plasma-EVs in this study. After performing basic characterizations of EVs, we investigated the effects of SA-NIC in response to menthol and AV on the packaging of nicotine-metabolizing CYP2A6 enzyme in plasma-derived EVs through western blot. We observed an increase in the EV packaging of CYP2A6 as compared to EV obtained ‘after’ SA-NIC with AV (p < 0.01) and SA-NIC with menthol (p < 0.01). No significant packaging of AOEs were observed. Next, we determined the levels of cytokines/chemokines in the plasma-EVs using multiplex ELISA. Interestingly, packaging of IL-1β was above 50% in EVs, both ‘before’ and ‘after’ SA-NIC, suggesting that IL-1β is circulating in plasma majorly via EVs. Next, we set out to evaluate the combined effects of BaP and plasma-EVs on the activation of cytokines/chemokines in SVGA cells. We observed that BaP significantly elevated IL-1β levels at 48h (P < 0.05). However, treatment of BaP and plasma EVs in combination did not significantly alter the levels of other cytokines/chemokines. This study provides evidence that nicotine in response to menthol and AV cues can package altered levels of CYP2A6, and cytokines/chemokines in plasma EVs. In addition, we suggest that BaP might contribute to the neuroinflammation by inducing IL-1β production in SVGA cells. Supported in part by funding from the NIH grant DA047178 (SK) and NIDA Grant DA047638 (HC).

## SNIP Early Career Investigators Travel Awards (ECITA): Pre-Doctoral Students

### In utero opioid exposure induced vulnerability to later life brain injury

Gowen, AM, BS ^1^.

*^1^Anesthesiology, University of Nebraska Medical Center, Omaha, NE 68106*.

Mild traumatic brain injury (mTBI) is a well observed phenomenon. However, the pathophysiology of mTBI is not well understood. As the field rapidly seeks to fill this lack of knowledge, there exists a comorbid sub-group of individuals who are not only more prone to the harmful biological sequelae from mTBI, but also more prone to mTBI themselves: neonatal abstinence syndrome (NAS) sufferers. The incidence of NAS caused by chronic prenatal opioid exposure is on the rise with a 2017 estimated 80 cases of newborns suffering from NAS occurring every day. NAS has been shown to cause cortical thinning, particularly in the motor cortex. NAS also increases future susceptibility to encephalopathies. Currently, there is no investigation regarding the impacts of NAS on later life mTBI. Our study seeks to elucidate how opioid NAS will impact mTBI sufferers during childhood. During childhood, cortical development is rapidly peaking and a potential mTBI at this time point could have severe inflammatory and pathophysiological effects. By analyzing physiological, inflammatory, and synaptic proteomics data of young rats post-mTBI we can characterize the differential response to injury between NAS subjects and normally developing subjects. Our work will provide insight into potential mechanisms of persistent changes associated with prenatal opioid exposure that could contribute to later-life neurological dysfunction.

### Using IPA tools to characterize molecular pathways underlying the involvement of IRF7 in antiviral Response to HIV

Kota, NK, BS ^1^ , Vigorito, MK, PhD^2^, Krishnan, VK, BS^1^, Chang, SL, PhD^1^.

*^1^Institute of Neurolmmune Pharmacology, Seton Hall University, South Orange, NJ 07079*.

*^2^Department of Psychology, Seton Hall University, South Orange, NJ 07079*.

Interferon Regulatory Factors (IRFS) regulate transcription of type-I interferons (IFNs) and IFN-stimulated genes. We previously reported that IFN-regulatory factor 7 (IRF7), is significantly upregulated in the brain of HIV-1 transgenic (HIV-1Tg) rats compared to F344 control rats in a region dependent manner. Our RNA deep-sequencing data were deposited in the NCBI SRA database with Gene Expression Omnibus (GEO) number GSE47474. Our current study utilized QIAGEN CLC Genomics Workbench and Ingenuity Pathway Analysis (IPA) to identify molecular pathways underlying the involvement of IRF7 in the HIV antiviral response. In addition, the GSE152416 dataset containing RNA data from HIV-1 positive patients was retrieved from the GEO database. The differential expression between HIV-Hg and F344 rats as well as HIV-positive and control patients was collected from GSE47474 and GSE152416, respectively. The IPA Pathway Explorer tool identified the 479 molecules associated with IRF7. The “Core Expression Data Analysis" function was used to identify the significant canonical pathways in the datasets with or without IRF7 and its associated molecules. It was found that IRF7 and its associated molecules increased the production of T-Helper 1 cells production (Thl), decreased the production of T-Helper 2 cells (TH2), and upregulated the levels of cAMP Response Element Binding Protein (CREB), resulting in the increased transcription of genes relating to neurogenesis, and memory consolidation. Overall, our meta-analyses demonstrate that IRF7 is crucial in the antiviral response to HIV viral proteins. Supported by NIH Grant R01DA0462582.

### Chimeric R5 simian-human immunodeficiency virus SHIV.D replicates in the brain, causes neuropathogenesis, and persists on antiretroviral therapy in rhesus macaques

Podgorski, RM ^1^, Robinson, JA^1^, Bar, K, MD^2^, Burdo, TH, PhD^1^.

*^1^Department of Microbiology, Immunology and Inflammation, Temple University Lewis Katz School of Medicine, Philadelphia, PA 19140*.

*^2^Department of Infectious Disease, University of Pennsylvania, Philadelphia, PA 19104*.

The characterization of persistent viral reservoirs is critical to ensure efficacy of a functional HIV cure. Thus, a non human primate model of persistence in viral reservoirs is necessary. Myeloid cells make up a critical and understudied component of HIV persistence in the central nervous system (CNS). Here, we demonstrate CNS replication and neuropathogenesis after antiretroviral therapy (ART) in rhesus macaques (RM) using novel macrophage-tropic transmitted/founder virus SHIV.D. SHIV.D encodes CCRS-tropic HIV-1 M subtype D env gene and replicates efficiently in RM macrophages and CD4+ T cells. We utilize SHIV.D to model HIV-1 infection and neuropathogenesis in RM. Six RM were infected with SHIV.D and established viral set points of >10"3 copies/ml. After establishment of infection, RM were treated with daily ART for 24 weeks. Following ART cessation, plasma virus rebounded to pre-ART setpoint in all RM. lmmunohistochemistry (IHC) and RNA in situ hybridization (ISH) were performed on brain tissue samples from RM necropsied while viremic and after additional >6 months on ART. Brain tissue of viremic RM revealed active viral replication as well as macrophage inflammation. In RM necropsied after additional >6 months suppressive ART, SHIV RNA decreased but macrophage inflammation persists. Dual IHC/ISH co staining revealed persistent SHIV.D replication in RM myeloid cells during viremia and suppression. The SHIV.D RM model will be used to further determine mechanisms of persistent neuroinflammation through ART in the CNS.

### Synthesis and characterization of a PLGA based cannabidiol (CBD) nanoformulation to treat neuropathic pain

Qayum, S, MS ^1^, Schmitt, R, MS^2^, Abbasi, Z, MD^1^, lgnatowski, T, MD^3^, Schwartz, SA, MD, PhD^1^, Prasad, PN, PhD^2^, Mahajan, SD, PhD^1^.

*^1^Department of Medicine, Division of Allergy, Immunology, and Rheumatology, Jacobs School of Medicine and Biomedical Sciences/University at Buffalo, Buffalo, NY 14203*.

*^2^Department of Chemistry, Institute for Lasers, Photonics and Biophotonics/University at Buffalo, Buffalo, NY 14203*.

*^3^Department of Pathology & Anatomical Sciences, Jacobs School of Medicine & Biomedical Sciences/University at Buffalo, Buffalo, NY 14203*.

Cannabidiol (CBD) has emerged as a promising drug for treating neuropathic pain. CBD is highly lipophilic and can cross the blood-brain barrier (BBB), but its bioavailability is limited, and clearance is quick, rendering its effectiveness in the brain. Our goal is to improve CBD solubility and increase its bioavailability in the brain, and thereby increase its effectiveness in the treatment of chronic neuropathic pain, by developing a novel mPEG-PLGA nanoparticle-based CBD nanoformulation. CBD was encapsulated within the mPEG-PLGA nanoformulation using an emulsion evaporation technique which enables production of nanoparticles in the size range ideal for BBB permeability. Nanoparticles were characterized for size and morphology using DLS (dynamic light scattering) and TEM (Transmission Electron Microscopy). The surface charge was determined using zetapotential analysis and the encapsulation efficiency was determined by UV-VISspectroscopy. The nanoformulation incorporated a CySdye that enabled visualizing cellular uptake and allowed examining BBB permeability. Our data indicate that the size of our nanoformulation is 38nm and the zeta potential was -22mV and the encapsulation efficiency of CBD mPEG-PLGA was 95%. Experiments to determine the percent BBB permeability of the nanoformulation using the in-vitro BBB model and assessment of the efficacy of the nanoformulation using an in-vivo pain management mouse model are ongoing. It is expected that this nanoformulation will improve the pharmacokinetic profile of CBD and enhance its efficacy against neuropathic pain. Supported by SR01DA047410-02.

### Investigating synaptic pruning by glia in HIV associated neurological dysfunction

Watson, Z^1^, Zheng, J^1^, Spurgat, M^1^, Ru, W^1^, Tang, SJ^2^.

*^1^Department of Neuroscience, Cell Biology, and Anatomy, University of Texas Medical Branch, Galveston, TX 775SS2*.

*^2^Department of Anesthesiology, Renaissance School of Medicine, Stony Brook University, Stony Brook, NY 11794*.

Synapse loss induced by neurotoxic viral proteins such as gp120 is implicated in the pathogenesis of HIV-associated neurological disorders such as pain and dementia. A recent study from our lab detailed a crucial signaling pathway required for gp120 to induce microglial synapse degeneration. However, the underlying synapse elimination mechanism remains elusive. Recent studies and our preliminary data have revealed a critical role for glia in synapse pruning, therefore we hypothesize that synapse pruning is a mechanism by which glial cells contribute directly to synapse loss induced by gp120. We compared glial synapse engulfment between wild type and gp120 transgenic mice (gp12otg) in the cortex and spinal dorsal horn. (n=3 per group). lmmunofluorescent staining was performed for markers of glial cells and synaptic material. 3D confocal images showed increased glial volume and cell body count suggesting activation of microglia and astrocytes in both tissues studied. Astrocyte engulfment of both Shank1 and vGlut1 increased from WT to gp120tg in the cortex (p=.002, p=l.Se-7). In the spinal dorsal horn, compared to WT, gp120tg, astrocyte engulfment of Shank1 appeared to increase (p=.06), and vGlut1 engulfment increased (p=.0006). There were no significant differences found in synaptic engulfment by microglia between WT and gp120tg in either tissue. These results suggest that synaptic pruning by astrocytes, but not microglia, is induced by gp120 in HIV associated neurological dysfunction. Supported by NIH R0l-NS-079166, NIH R0l-NS-095747, NIDA/NIH R0l-DA-036165.

## SNIP Early Career Investigators Travel Awards (ECITA): Post-Doctoral Fellows and Junior Faculty

### Discovery and functional characterization of HIV-1-latency associated circular RNAs in African American women living with HIV

Bellizzi, A, PhD^1^, Kaminski, R, PhD^1^, Cipriaso, JM, BS^1^, Wollebo, HS, PhD^1^, Sariyer, IK, PhD^1^.

*^1^Department of Microbiology, Immunology and Inflammation, Lewis Katz School of Medicine at Temple University, Philadelphia, PA 19140*.

The main obstacle to HIV cure is the persistence of the reservoir of latently infected cells that harbor the replication-competent virus. Moreover, continued chronic neuroinflammation in people with HIV (PWH) leads to the development of the HIV-associated neurocognitive disorder (HAND) in over 40% of PWH. The lack of reliable biomarkers of persistent viral reservoir and HAND prevents the development of successful targeted therapies. Circular RNAs (circRNAs) are new class of unique non-coding RNAs derived from non-canonical splicing of their cognate mRNA. Our preliminary microarray-based profiling of circRNA expression in PBMCs from 19 virally suppressed African American women living with HIV identified a set of highly differentially expressed circRNAs compared to the healthy donors. We have manipulated the expression levels of selected circRNAs by overexpression or Cas13d-mediated degradation and analyzed the effects on virus reactivation in latently infected T cell line (JLatl0.6). Interestingly, circ000780 derived from the FAM107B gene of unknown function, was consistently downregulated in almost all patient samples and in also JLatl0.6 cells. Moreover, overexpression of this circRNA resulted in reactivation of the latent virus in a subset of transfected cells. Additionally, we identified a set of circRNAs having expression levels correlated with the neurocognitive status of HIV-positive patients. In conclusion, latently HIV- 1 infected cells have a distinct circRNA signature, which can be further exploited to develop novel biomarkers and therapeutic modalities to HIV.

### Methamphetamine induces the release of proadhesive extracellular vesicles and promotes syncytia formation: A potential role in HIV-1 neuropathogenesis

Chand, S, PhD ^1^ , DeMarino, C, PhD^2^, Gowen, A^1^, Cowen, M^2^, Al-Sharif, S^2^, Kashanchi, F, PhD^2^, Yelamanchili, SV, PhD^1^.

*^1^Department of Anesthesiology, University of Nebraska Medical Center, Omaha, NE 68198*.

*^2^Laboratory of Molecular Virology, School of Systems Biology, George Mason University, Manassas, VA 20110*.

Despite the success of antiretroviral therapy (ART), the high prevalence of HIV-1-associated neurocognitive disorders (HAND) poses a significant challenge for the general well-being of people living with HIV (PWH). Methamphetamine (meth) and related compounds are among the most commonly used illicit drugs. Intriguingly, PWH who are meth users have a comparatively higher rate of HAND and exhibit a higher viral load in the brain. Effectively, all cell types secrete nano-sized lipid membrane vesicles, referred to as extracellular vesicles (EVs), that can function as intercellular communication to modulate the physiology and pathology of the cells. This study shows that meth treatments on chronically HIV-infected promonocytic U1 cells induce the release of EVs that promote cellular clustering and syncytia formation, a phenomenon that facilitates HIV pathogenesis. Our analysis also revealed that meth exposure increased intercellular adhesion molecule-1 (ICAM-1) and HIV-Nef protein expression in both large (10 K) and small (100 K) EVs. Further, when meth EVs were applied to uninfected naïve monocyte-derived macrophages (MDMs), we saw a significant increase in cell clustering and syncytia formation. Furthermore, treatment of MDMs with antibodies against ICAM-1 and its receptor, lymphocyte function-associated antigen 1 (LFA1), substantially blocked syncytia formation and reduced the number of multinucleated cells. In summary, our findings reveal that meth exacerbates HIV pathogenesis in the brain by releasing proadhesive EVs, promoting syncytia formation and HIV infection.

### MU opioid receptor-mediated release of endolysosome iron increases levels of mitochondrial iron, reactive oxygen species, and cell death

Halcrow, PW, PhD ^1^, Kumar, N, MS^1^, Hao, E, BS^1^, Khan, N, PhD^1^, Meucci, O, MD, PhD^2^, Geiger, JD, PhD^1^.

*^1^Department of Biomedical Sciences, University of North Dakota School of Medicine and Health Sciences, Grand Forks, ND 58201*.

*^2^Department of Physiology and Pharmacology, Drexel University School of Medicine, Philadelphia, PA 19104*.

Opioids are widely used analgesics and prone to abuse due to their euphoric and dependency effects.

Functioning through opioid receptors, opioids including morphine and DAMGO activate mu-opioid receptor (MOR), increase intracellular reactive oxygen species (ROS) levels, and induce cell death. Ferrous iron (Fe^2+^) through Fenton-like chemistry increases ROS levels. Endolysosomes are “master regulators of iron metabolism" central to iron trafficking as they contain readily-releasable Fe^2+^ stores and an acidic lumen linked to iron homeostasis. However, the opioid-induced mechanisms influencing endolysosome-iron homeostasis and downstream-signaling events are unknown. Using SH-SYSY neuroblastoma and U87MG astrocytoma cells we found morphine and DAMGO de-acidified endolysosomes, decreased endolysosome Fe^2+^ levels, increased cytosol and mitochondria Fe^2+^ and ROS levels, depolarized mitochondrial membrane potential, and induced cell death; effects blocked by the nonselective and selective MOR-antagonists naloxone and β-funaltrexamine. Deferoxamine, the endolysosome-iron chelator, inhibited opioid-induced increases in cytosolic and mitochondrial Fe^2+^ and ROS; the endolysosome-resident two-pore channels inhibitor NED-19 and the mitochondrial permeability transition pore inhibitor TRO blocked these opioid induced effects. Thus, opioid-induced increases in cytosolic and mitochondrial Fe^2+^ and ROS and cell death appear downstream of endolysosome de-acidification and Fe^2+^ efflux. These findings provide new insight into opioid pharmacological actions and new therapeutic possibilities. Supported by P30GM100329, U54GM115458, R01MH100972, R01MH105329, R01MH119000, 2R01NS065957, 2R01DA032444.

### Abnormal brain diffusivity in participants with persistent neuropsychiatric symptoms after COVID-19

Liang, H, PhD^1^, Ernst, T, PhD^1^, Oishi, K, MD, PhD^2^, Ryan, M, MS^1^, Wilson, E, MD^3^, Levine, A, MD^4^, Cunningham, E, BS^1^, Kottilil, S, MD, PhD^3^, Chang, L, MD^1^.

*^1^Diagnostic Radiology and Nuclear Medicine, University of Maryland School of Medicine, Baltimore, MD 21201*.

*^2^Department of Radiology, Johns Hopkins University School of Medicine, Baltimore, MD 21287*.

*^3^Institute of Human Virology, Department of Medicine, Division of Infectious Disease, University of Maryland School of Medicine, Baltimore, MD 21201*.

*^4^Department of Medicine, Division of Pulmonary & Critical Care Medicine, University of Maryland School of Medicine, Baltimore, MD 21201*.

Background: Neuropsychiatric symptoms are common in individuals with post-acute sequelae of COVID-19 (PASC). Several studies found participants had altered white matter integrity within 6 months after COVID-19 regardless of PASC symptoms. Methods: We compared 23 participants with PASC (182 days (range: 42-484 days) since COVID-19 diagnosis, 8 men) to 24 COVID- controls who never had COVID-19 symptoms (11 men) on cognitive function (NIH Toolbox®), emotions (PROMIS®), and diffusion tensor imaging metrics. Fractional anisotropy (FA), axial, radial, and mean diffusivities (MD) were assessed using an automated atlas in 9 white matter and 6 subcortical brain regions. Results: Compared with controls, PASC participants had higher FA in bilateral sagittal strata (SS), right superior longitudinal fasciculus (SLF), and lower diffusivities in the right cingulum, (p-values=0.004-0.041). In women, PASC had higher left amygdala MD than controls (Sex*PASC-p=0.006). Lower SS-FA predicted less fatigue in all participants (r=D.515, p<0.001). Higher left amygdala-MD predicted greater fatigue (r=0.613, p<0.001) and anxiety (r=D.687, p<D.001) across all women, but higher perceived stress (r=0.449, p=0.002) in all participants. Conclusions: The microstructural alterations in the PASC group are similar to those in stress-related disorders. These diffusivity-related PASC symptoms might be attributed to stress. Supported by NIH/R21NS121615.

### Disruption of choice: EcoHIV inoculation following a history of cocaine use

McLaurin, KA, PhD ^1^, Li, H, MD, PhD^1^, Mactutus, CF, PhD^1^, Harrod, SB, PhD^1^, Booze, RM, PhD^1^.

*^1^Cognitive and Neural Science Program, University of South Carolina, Columbia, SC 29208*.

Independently, chronic cocaine use and HIV-1 viral protein exposure induce neuroadaptations in the mesolimbic DA system; how the mesolimbic DA system responds to HIV-1 infection following chronic drug use, however, has remained elusive. The innovative chimeric HIV (EcoHIV) rat heralds an opportunity to investigate these comorbidities using a pretest-posttest design. After a history of sucrose and cocaine self-administration, male and female rats were evaluated in a concurrent choice self-administration paradigm for 5 days, whereby rats responded for either sucrose (5% w/v) or cocaine (0.33 mg/kg/inf). During the pretest assessment, male rats exhibited a significantly greater number of responses (preference) for cocaine, whereas female rats preferred sucrose. Subsequently, rats received retroorbital injections of either saline (male, n=9, female, n=9) or EcoHIV (male, n=10, female, n=10) and were reevaluated. Three days after inoculation, both EcoHIV male and female rats failed to exhibit a preference for either cocaine or sucrose. Six weeks after inoculation, EcoHIV male rats developed a preference for sucrose, whereas EcoHIV females, again, failed to exhibit a reinforcer preference. Saline male and female rats maintained their preference for cocaine or sucrose, respectively, during both posttest evaluations. EcoHIV rats exhibited a prominent disruption in choice behavior despite no alterations in sensitivity to sucrose or cocaine. Thus, the EcoHIV rat affords a biological system to model how the mesolimbic DA system responds to HIV-1 infection following chronic drug use. Supported by DA013137, MH106392, NS100624.

## Symposium 4: HIV and neurological diseases

### LM11A-31 as an anti-stroke and anti-HIV drug candidate

Mirzahosseini, G ^1^, Kodidela, S, PhD^1^, Sinha, N^1^, Zhou, L^1^, Ishrat, T, PhD^1^, Kumar, S, PhD^1^.

*^1^Pharmaceutical Sciences, University of Tennessee Health Science Center, Memphis, TN 38163*.

The existing antiretroviral therapy (ART) drugs cannot cross the blood-brain barrier and have suboptimal brain concentration. In this study, we found a new drug candidate , LM11A-31, a p75 neurotrophin receptor (P75^NTR^) ligand for both stroke and HIV. The drug is hydrophilic and crosses the BBB, which can be a major advantage compared to the ART drugs. In this study, we found that LM11A-31 suppresses HIV replication without causing toxicity on HIV-infected cells and significantly reduces infarction volume in mice following stroke. We studied concentration (10-1000 nM)- and time-dependent (1-3 days) effect of LM11A-31 on an HIV-infected macrophage cell lines (U1). We found that 100 nM of the drug could suppress the viral load in U1 cells on day 2. Our results also showed that 100 nM of LM11A-31 does not cause toxicity on day 2 and day 3 and significantly reduces toxicity at day 1. Further, our results illustrated that combination therapy of LM11A-31 and DRV reduced the protein expression of IL-1B on day 2, which is known to be upregulated upon HIV infection. Also, LM11A-31 decreased the protein expression of superoxidase dismutase and catalase on day 2, which is consistent with reduced toxicity and total antioxidant capacity. Moreover, we found that LM11A-31 remarkably reduced hemorrhagic transformation and brain edema 24 h after stroke in mice. LM11A-31 reduced inflammatory markers after stroke compared to the control. This study suggests that LM11A-31 may be a potential therapeutic agent for both suppressing HIV replication in the brain and improving the outcome of stroke. Supported by MH125670 and DA047178.

### Molecular and cellular impact of morphine and HIV-1 NEF-EVs on OPRM1 pre-mRNA splicing

Sariyer, IK, PhD^1^, Donadoni, M, PhD^1^, Huang, W, BS^2^, Yarandi, S, PhD^1^, Burdo, TH, PhD^1^, Chang, SL, PhD^2^.

*^1^Department of Microbiology, Immunology, and Inflammation, Temple University School of Medicine, Philadelphia, PA 19140*.

*^2^Institute of NeuroImmune Pharmacology and Department of Biological Sciences, Seton Hall University, South Orange, NJ 07079*.

Clinically used opioids, such as morphine, activate the mu opioid receptor (MOR) encoded by Opioid Receptor Mu 1 (OPRM1) gene. Examination of the opioid receptor genes showed that the human OPRM1 pre-mRNA undergoes extensive alternative splicing events and capable of expressing 21 isoforms. However, characterization of OPRM1 signaling is generalized, and only one isoform (MOR-1) has been extensively studied. Compounding this issue is the increasing significance of intravenous drug abuse in HIV neuropathogenesis. Here, we investigated the molecular impact of morphine and HIV-1 on regulation of OPRM1 pre-mRNA splicing in in vitro and in vivo models. Our results suggested that morphine treatment specifically induces the alternative splicing of MOR-1X isoform among the other isoforms analyzed in neuronal cells. Interestingly, alternative splicing and expression of MOR-1X isoform was also induced in postmortem brain tissues obtained from people with HIV (PWH). Additionally, treatment of control rats with morphine induced alternative splicing of MOR-1X in the brain regions involved in the reward pathways. More interestingly, HIV-1 transgenic (HIV-1Tg) rats, showed an additive induction of MOR-1X isoform with the exposure to morphine. To further assess the possible role of HIV secretory proteins in alternative splicing of OPRM1 gene, we analyzed the impact of HIV-1 Tat, gp120 and Nef proteins on alternative splicing of MOR-1X isoform. While the Tat and gp120 had no visible effects, treatment of neurons with Nef induced MOR-1X alternative splicing that was comparable to treatment. Supported by NIH/NIDA.

### Novel allosteric modulator attenuates HIV-1 tat protein-induced inhibition of dopamine transporter and alleviates cognitive and cocaine rewarding effects in HIV-1 tat transgenic mice

Zhu, J, MD, PhD ^1^, Davis, S, MS^1^, Jimenez-Torres, A, PhD^1^, Ferris, M, PhD^2^, Zhan, CG, PhD^3^, McLaughlin, J, PhD^4^, Augelli-Szafran, C, PhD^5^.

*^1^College of Pharmacy, University of South Carolina, Columbia, SC 29208*.

*^2^School of Medicine, Wake Forest University, Winston-Salem, NC 27109*.

*^3^College of Pharmacy, University of Kentucky, Lexington, KY 40536*.

*^4^College of Pharmacy, University of Florida, Gainesville, FL 32611*.

*^5^Department of Chemistry, Southern Research Institute, Birmingham, AL 35205*.

We have demonstrated that HIV-1 Tat protein allosterically modulates dopamine reuptake via dopamine transporter (hDAT). This study determined whether a novel allosteric modulator, SRI-32743, ameliorates the effects of Tat binding to DAT and alleviates Tat-induced potentiation of cognitive impairment by novel object recognition (NOR) testing and cocaine reward by cocaine-conditioned place preference (CPP) in inducible Tat transgenic (iTat-tg) mice. SRI-32743 (50 nM) in vitro inhibited [3H]DA uptake and [3H]WIN35,428 binding, and decreased the affinity of cocaine inhibiting [3H]DA uptake in combination with cocaine compared to cocaine alone in cells expressing hDAT. SRI-32743 decreased the cocaine-induced dissociation rate of [3H]WIN35,428 binding and attenuated Tat protein-inhibited [3H]DA uptake and [3H]WIN35,428 binding. Induction of Tat expression in iTat-tg mice by a 14-day administration of doxycycline resulted in a 31.7% reduction of phase 3 recognition index in NOR and a 2.7-fold potentiation of cocaine-CPP compared to the respective vehicle-treated iTat-tg mice. Systemic administration (i.p.) of SRI-32743 prior to behavioral testing ameliorated Tat-induced impairment of NOR (at a dose of 10 mg/kg) and the Tat-induced potentiation of cocaine-CPP (at a dose of 1 or 10 mg/kg). No effect was observed in saline-treated (uninduced) iTat-tg or doxycycline-treated G-tg (Tat-null) mice. These findings validate that Tat and cocaine interaction with DAT can be modulated through an allosteric modulation manner, suggesting a potential therapeutic intervention for HAND. Supported by NIH/DA035714 and DA041932.

### Abnormal β-amyloid accumulation: Aging HIV-1 human and HIV-1 transgenic rat brain

Booze, RM, PhD ^1^, Li, H, MD^1^, McLaurin, KA, PhD^1^, Mactutus, CF, PhD^1^, Likins, B, BS^1^, Huang, W, PhD^2^, Chang, SL, PhD^2^.

*^1^Cognitive and Neural Sciences, University of South Carolina, Columbia, SC 29208*.

*^2^Department of Biological Sciences, Seton Hall University, South Orange, SC 07079*.

The prevalence of HIV-associated neurocognitive disorders (HAND) is significantly greater in older, relative to younger, HIV-1 seropositive individuals; the neural pathogenesis of HAND in older HIV-1 seropositive individuals, however, remains elusive. To address this knowledge gap, abnormal protein aggregates (i.e., β-amyloid) were investigated in post-mortem HIV-1 seropositive individuals with HAND and in the brains of aging (12-14 month old) HIV-1 transgenic (Tg) rats. First, in post-mortem HIV-1 seropositive individuals with HAND, intraneuronal β-amyloid accumulation was observed in the hippocampal dentate gyrus and dorsolateral prefrontal cortex, although no amyloid plaques were found in these brain regions. Second, in aging HIV-1Tg rats, immunohistochemistry and western blots revealed abnormal intraneuronal β-amyloid accumulation in the hippocampus and prefrontal cortex (PFC) of HIV-1 Tg rats, relative to F344/N control rats. Critically, β-amyloid was co-localized with neurons in the hippocampus and cortex regions, supporting a potential mechanism underlying synaptic dysfunction in the HIV-1 Tg rat. Consistent with these neuropathological findings, HIV-1 Tg rats exhibited prominent alterations in the progression of temporal processing relative to control animals. Collectively, intraneuronal β-amyloid aggregation observed in the hippocampus and prefrontal cortex of both HIV-1 seropositive individuals and the HIV-1 Tg rat supports a potential factor underlying HIV-1 associated synaptodendritic damage and HAND. Supported by National Institutes of Health (NIH) grants MH106392, DA013137, NS100624, U24MH100931, U24MH10093, U24MH10092.

## Symposium 5: Selected from the submitted abstracts

### Dolutegravir inhibition of matrix metalloproteinases: Functional mechanism underlying developmental neuro-abnormalities

Bade, AN, PhD ^1^, Foster, E, BS^1^, Liu, Y, PhD^2^, Edagwa, B, PhD^1^, Gendelman, HE, MD^1^.

*^1^Department of Pharmacology and Experimental Neuroscience, University of Nebraska Medical Center, Omaha, NE 68198*.

*^2^Department of Radiology, University of Nebraska Medical Center, Omaha, NE 68198*.

Dolutegravir (DTG) is a first-line antiretroviral drug used in combination therapy for the treatment of human immunodeficiency virus type-1 (HIV-1) infection. Due to roll out of generic DTG-based regimen and rising pretreatment resistance to non-nucleoside reverse transcriptase inhibitors in resource limited countries, 15 million HIV-1 infected people will be treated with DTG by year 2025. This includes women of child-bearing age who remain a significant infected population. However, growing data have suggested that DTG is associated with birth defects and postnatal developmental neurologic abnormalities following periconceptional usages. To this end, uncovering an underlying mechanism for DTG-associated adverse fetal development outcomes has gained research interest. We now report that DTG inhibits matrix metalloproteinases (MMPs) activities that could affect fetal neurodevelopment. DTG was found to be a broad-spectrum MMPs inhibitor. It was more potent MMPs inhibitor than doxycycline. DTG was found to bind Zn++ at the catalytic domain to inhibit MMPs activities. Moreover, inhibition of MMPs was found to be a INSTI class effect. Studies performed in pregnant mice showed that DTG readily reaches to the fetal CNS during gestation and inhibits MMPs activity during critical period of brain development. Further postnatal evaluation of brain health in mice pups identified neuroinflammation and neuronal damage following in utero DTG exposure. Thus, we conclude that DTG inhibition of MMPs activities during gestation has the potential to affect pre- and post-natal neurodevelopment. Supported by NICHD.

### Persistent diastolic dysfunction in DTG/TDF/FTC-treated HIV-infected NSG humanized mice

Bidasee, KR, PhD ^1^, Dash, PK, PhD^1^, Alomar, FA^2^, Guo, L^1^, Matthews, S^1^, Gorantla, S^1^.

*^1^Department of Pharmacology and Experimental Neuroscience, University of Nebraska Medical Center, Omaha, NE 68130*.

*^2^Department of Pharmacology and Toxicology, College of Clinical Pharmacy, Imam Abdulrahman Bin Faisal University, Dammam, 31441*.

Nearly 50% of persons living with HIV-1 infection (PLWH) have early-onset cardiac diastolic dysfunction (DD), a harbinger for diastolic heart failure and frequent hospital visits. Therapeutic strategies to blunt DD in PLWH are virtually non-existent as its molecular causes remain poorly understood. Here we show using multi-modal echocardiography that HIV-infected NOD.Cg-PrkdcscidIl2rgtm1Wjl/SzJ humanized mice (Hu-mice) treated with first line antiviral combination dolutegravir/tenofovir disoproxil fumarate/emtricitabine via feed for 10 weeks to suppressed plasma viremia below detection levels, developed degrees II/III DD. In vivo photo-acoustic imaging, a hybrid technology based on laser excitation of hemoglobin with echocardiography, revealed significant reductions in saturated oxygenated hemoglobin flow in the vasculature of the anterior walls. Hearts from DTG/TDF/FTC-treated HIV-infected Hu-mice injected with BSA-fluorescein isothiocyanate conjugate (50 µl of 40 mg/mL) ten minutes prior to sacrifice also revealed significant micro-ischemia and vascular leakage, akin to that reported in PLWH. The hypoxia-induced transcription factor HIF-1a and the cytotoxic glycolysis metabolite methylglyoxal were also ∼3-fold higher in hearts from untreated and DTG/TDF/FTC-treated HIV-infected Hu-mice compared to uninfected controls. These data are the first to show that DTG/TDF/FTC-treated HIV-infected Hu-mice can recapitulate the DD reported in PLWH. They also suggest correlations between DD, HIF-1a, microvascular damage/leakage and ischemia in the Hu-mouse model and PLWH. Supported by R56 HL151602-01A1.

### Potentially beneficial effects of cannabidiol (CBD) in ecoHIV infection in culture and mice

Borjabad, A, PhD ^1^, Kelschenbach, J, PhD^1^, Carvallo, L, PhD^1^, Hadas, E, PhD^1^, Volsky, DJ, PhD^1^.

*^1^Department of Medicine/Division of infectious diseases, Icahn School of Medicine at Mount Sinai, New York, NY 10029*.

Cannabidiol (CBD), a non-psychoactive cannabis component, is freely available and widely used for relieving pain and anxiety commonly experienced by people living with HIV (PLWH) and it has been described as having several anti-inflammatory effects. However, its long-term effects on HIV infection and progression of HIV neurocognitive disease remain largely undetermined. Here, we use in vitro models and EcoHIV infected mice to investigate the effects of CBD on virus load, inflammatory markers, brain transcriptional profiles, and behavioral outcomes. Following kinetic and dose optimization studies, our preliminary results show a reduction of EcoHIV viral load in monocyte/macrophage-derived RAW 264.7 and microglia-derived SIM-A9 cell lines and in primary murine macrophages pre-treated with CBD. Virus load was also reduced in the brain of EcoHIV infected mice pretreated with 30 mg/kg of CBD. This effect was accompanied by altered transcriptional signature in brain tissue compared to untreated infected mice that included down-regulation of immune-related genes TNF-α, CXCL10 and C3. However, CBD treatment of EcoHIV mice had no significant effect on neurocognitive disease in these animals. Our data indicate that CBD can have beneficial effects in mitigating some HIV effects in vitro and in vivo. Supported by NIDA R01 DA052844-01 /NIDA U01 DA053629.

### Traumatic brain injury-induced persistent inflammation is associated with long-term behavioral deficits

Cheeran, MCJ, PhD ^1^, Krishna, VD, PhD^1^, Emmit, NE, MS^1^, Crane, AT, PhD^2^, Patterson, M^3^, Williams, J, PhD^3^, Grande, AW, MD, PhD^2^, Low, WC, PhD^2^.

*^1^Veterinary Population Medicine, College of Veterinary Medicine, University of Minnesota, St. Paul, MN 55108*.

*^2^ Department of Neurosurgery, University of Minnesota Medical School, Minneapolis, MN 55455*.

*^3^ Department of Integrative Biology & Physiology, University of Minnesota Medical School, Minneapolis, MN 55455*.

Traumatic brain injury (TBI) is associated with impairment of physical, cognitive, and psychosocial function. Evidence from experimental and clinical studies indicates that TBI increases the risk of developing substance use disorder and this risk is strongly associated with the inflammatory response to injury. A controlled cortical impact murine model was used to characterize neuroinflammation to mild and moderate TBI. We found that monocyte/macrophages infiltration into the brain peaked at 3 days post-injury (dpi) in both TBI models, with increased numbers of activated CD86+ and MHC II+ macrophages. While most immune cells returned to sham-injured levels by 15 dpi, higher numbers of brain macrophages expressing CD86 were seen at 15 and 30 dpi, even with mild TBI, indicative of persistent neuroinflammation. Although fate mapping using CCR2/CX3CR1 dual reporter mice confirmed immigration of macrophages at 3 dpi, we found that infiltrating macrophages were not derived from circulation or peripheral bone marrow using bone marrow chimeras. In addition, while motor function in injured animals returned to baseline levels with the resolution of acute neuroinflammation, animals with TBI exhibited spatial learning deficits on the Barnes maze assay and showed increased sensitivity to morphine in a conditioned place preference test. Injured animals showed stronger place preference even at lower morphine doses. These results suggest that persistent macrophage activation associated with TBI may influence behavioral outcomes like substance use disorder. Supported by Minnesota Spinal Cord and Traumatic Brain Injury Research Grant Program, MN Office of Higher Education and T32 DA007097, NIDA.

### Methamphetamine potentiation of HIV-1 gp120-associated microglia neuroinflammation via NLRP3 activation

Dutta, D, PhD ^1^, Liu, JN, MD, PhD^1^, Xu, EQ, MD, PhD^1^, Xiong, H, MD, PhD^1^.

*^1^Neurophysiology Laboratory, Department of Pharmacology and Experimental Neuroscience, University of Nebraska Medical Center, Omaha, NE 68198*.

HIV-1-associated neurocognitive disorder (HAND) remains highly prevalent in infected patients despite combined antiretroviral therapy (cART). Methamphetamine (Meth) abuse exacerbates HAND. The mechanisms for Meth exacerbation of HAND are not fully understood. Viral protein gp120 has been identified as a potent neurotoxin and activation of microglia inflammasome plays an important role in the HAND pathogenesis. It is our hypothesis that Meth exacerbates HAND by enhancing gp120-induced, microglia-associated neuroinflammation. To test this hypothesis, we studied the effects of Meth and gp120 on microglial NLRP3 activation and IL-1β production in primary rat microglial cultures. Our results showed Meth enhanced gp120-induced microglia activation detected by immunofluorescence labeling of Iba-1 expression and potentiated gp120-induced IL-1β processing and secretion assayed by western blot and ELISA. Meth also induced the colocalization of NLRP3 and caspase-1 in gp120-primed microglia and increased total ROS production synergistically with gp120. The Meth-associated potentiation of IL-1β processing and secretion in gp120-primed microglia was significantly attenuated by an NLRP3 inhibitor MCC950 or by a mitochondrial superoxide scavenger Mito-TEMPO. These results demonstrated that Meth potentiated gp120-associated microglia NLRP3 inflammasome activation via ROS signaling. Supported by NIH grant 5R01DA050540.

### HIV infection primes the innate immune response to methamphetamine

Xu, J-C, PhD^1^, Yoo, SW, PhD ^2^, Deme, P, PhD^2^, Dawson, VL, PhD^1^, Haughey, NJ, PhD^2^.

*^1^Institute for Cellular Engineering, Johns Hopkins University School of Medicine, Baltimore, MD 21210*.

*^2^Department of Neurology, Johns Hopkins University School of Medicine, Baltimore, MD 21210*.

Methamphetamine use by people infected with HIV increases viral loads, immune activation, and levels of inflammatory cytokines. Here we used microglia colonized 3-D human forebrain organoids to investigate the effects of HIV (FOR-organoidsMicro+/HIV+) and methamphetamine on innate immune function. In microglia, HIV-Bal infection was characterized by steady increases of viral production from day 1-25. HIV-Ada infection was below detectable limits days 1-6, followed by a low-level of viral replication until day 20, and a linear increase in replication until day 25. HIV-RF infection was characterized by low-level replication over the first 4 days followed by a reduction to very low levels of replication for the remaining 25 days. In FOR-organoidsMicro+/HIV+ concentrations of multiple ceramides were increased and the corresponding sphingomyelins decreased. Inhibition of nSMase2 reduced concentrations of several ceramides and increased levels of the corresponding sphingomyelins. The addition of Meth to FOR-organoidsMicro+/HIV+ did not further increase ceramides, but robustly increased secretion of IL-1-beta, TNF-alpha, IL-6, IL-2, IL-4, IL-12p70 and IL-10. Meth-induced secretion of inflammatory cytokines was blunted by inhibition of nSMase2 with PDDC. These data suggest that HIV infection primes the innate immune system to robustly respond to Meth. This priming event involves nSMase2 and the bioactive lipid ceramide. These preliminary findings are consistent with the emerging concept of innate immune memory, where a glial phenotype adapts its response to previous exposures. Supported by NIDA DA052272.

### CSF anti-CD4 autoantibodies and neuroHIV pathogenesis

Jiang, W, MD ^1^, Cheng, D, MS^1^, Luo, Z, PhD^1^, Ndhlovu, L, MD^4^, Gisslen, M, MD^2^, Price, R, MD^3^.

*^1^Department of Microbiology and Immunology, Medical University of South Carolina, Charleston, SC 29425*.

*^2^Sahlgrenska Academy, University of Gothenburg, 41645, Gothenburg, Sweden, University of Gothenburg, Sweden, Gothenburg, 41645*.

*^3^San Francisco General Hospital, University of California San Francisco, San Francisco , CA 94110*.

*^4^Department of Medicine, Division of Infectious Diseases, Weill Cornell Medicine, New York, NY 10065*.

Background. Our previous work has shown the pathologic role of anti-CD4 IgG autoantibodies in blunted CD4+ T cell recovery in people living with HIV (PWH) with antiretroviral therapy (ART) and viral suppression. Methods. In the current study, 35 ART-naïve PWH, 24 aviremic and ART-treated PWH and 16 HIV seronegative controls had plasma and cerebrospinal fluid (CSF) anti-CD4 IgG antibodies measured by autoantibody protein array. CSF markers of neuroinflammation, neuronal injury and blood-brain barrier (BBB) permeability were assessed as well. BBB permeability was evaluated by the CSF to serum albumin quotient (QAlb) by nephelometry. CSF neurofilament light chain (NfL) concentration was measured by a sensitive immunoassay using an ELISA kit; intra-assay coefficients of variation were below 10%. Since CSF NfL changes with age, CSF NfL levels were age-adjusted to 50 years for comparisons across subjects, and considered normal if below 967 ng/L. The cutoff value to separate high versus low anti-CD4 IgG level was set by 95 percentile of controls had both CSF and plasma anti-CD4 IgG levels lower than 50 ng/mL, as well as by isolated anti-CD4 IgGs from plasma levels of 50 ng/mL in PWH have been shown to mediate antibody dependent cytotoxicity (ADCC) against CD4+ T cells. The differences in continuous measurements were analyzed by non-parametric Mann-Whitney U tests for two-group comparisons and by the ANOVA and Kruskal-Wallis tests for more than two-group comparisons. Correlations were analyzed by Spearman correlation tests. Age adjusted P values were calculated by ANCOVA. Supported by NIAID R01-AI128864.

### Long-term changes of neurotransmission-related gene expression correlate with impaired behavioral performance caused by methamphetamine and neuroHIV

Kaul, M, PhD ^1^, Shah, R, BS^1^, Maung, R, BS^1^, Ojeda-Juarez, D, PhD^2^, Harahap-Carrillo, IS, MS^1^, Roberts, AJ, PhD^4^, Sanchez, AB, PhD^3^, TMARC, Group^3^.

*^1^School of Medicine, Division of Biomedical Sciences, University of California, Riverside, Riverside, CA 92521*.

*^2^Center for Infectious and Inflammatory Disease, Sanford Burnham Prebys Discovery Medical Institute, La Jolla, CA 92037*.

*^3^Department of Psychiatry, University of California, San Diego, San Diego, CA 92093*.

*^4^Animal Models Core, The Scripps Research Institute, La Jolla, CA 92037*.

Methamphetamine (METH) use is frequent among people living with HIV-1 and aggravates HIV-associated neurocognitive disorders (HAND). Yet, the underlying pathological mechanisms are incompletely understood. Transgenic mice expressing a soluble viral envelope protein gp120 of HIV-1 in the brain (gp120tg) share key neuropathological features with NeuroHIV/AIDS patients. We previously exposed young gp120tg mice to an escalating METH binge regimen for 25 days and analyzed the animals 7 months afterwards. We found that METH and gp120 exposure caused besides neuronal injury also behavioral impairment and lasting differential expression of neurotransmission-related genes. For this study, we correlated neurotransmission-related gene expression with gp120 and METH exposure and with behavioral performance. Significant correlations were found for gp120 and METH in both cortex and hippocampus, indicating that neurotransmitter receptors, such as Htr7 and Gria 3, as well as transporters, such as Slc7a11, and signaling factors, such as Grk6, are affected by METH, HIV and their combination. However, behavioral outcomes for spatial learning and memory (Barnes Maze, % time in target quadrant, spatial strategy, errors) correlated most significantly with different genes of all three categories, such as receptors Gabra1, Gabrg3, Htr2b, transporter Slc32a1, and signaling factors Akt1, Pkb3, Sncaip. Thus, METH and HIV affect neurotransmission and behavior in a lasting fashion at multiple levels, and our study provides a framework for the future identification of causal gene networks. Supported by NIH, MH087332, DA052209 and DA026306.

### Role of gut microbiome in opioid withdrawal-induced anxiety and depression-like behaviors in HIV-1 transgenic mice

Moidunny, S, PhD ^1^, Kolli, U, PhD^1^, Oppenheimer, M, BS^1^, Valdez, E, BS^1^, Chupikova, I, MD^1^, Roy, S, PhD^1^.

*^1^Department of Surgery, University of Miami Miller School of Medicine, Miami, FL 33136*.

Neuropsychiatric disorders such as anxiety and depression are prevalent in HIV-1 patients using opioids and are associated with poor adherence to antiretroviral therapy and higher mortality rates. Growing evidence indicate that the gut microbiome, and compounds it produces, may play a role in mental health conditions. Thus, understanding the gut-brain axis in HIV-associated anxiety and depression will open new avenues for therapeutic intervention. We have recently shown that chronic opioid use causes gut microbial dysbiosis, gut epithelial barrier disruption and subsequent translocation of the gut microbes and/or bacterial products to circulation in mice, resulting in systemic inflammation. In this study, we investigated whether or not these effects of morphine are associated with development of anxiety and depression-like behaviors in HIV-1 transgenic mice and wild-type mice. Mice were spontaneously withdrawn from morphine and were subjected to a battery of behavioral tests: open-field test, elevated plus maze, sociability and tail suspension test. At the end of behavioral testing, mice were euthanized and tissues (brain and gut) were collected for immunohistochemistry and molecular analyses. Fecal samples were analyzed using Nextera HiSeq 2500 sequencing (shotgun metagenomics). We provide evidence that morphine-induced dysbiosis of the gut bacteria is associated with anxiety and depression-like behaviors, more severely in HIV-1 transgenic mice. Ongoing studies will determine whether restoration of these gut bacteria will rescue the behavioral abnormalities in these mice. Supported by NIDA.

## SNIP Regular Conference Posters:

### Drugs repurposing for the treatment of HIV-associated neurocognitive disorder by AI-based literature mining

Aksenova, M, PhD^1^, Ciu, C^1^, Sybrand, J^4^, Tyagin, I^4^, Odhiambo, D^1^, Lucius, M^1^, Ji, H^1^, Pena, E^3^, Lizarraga, S^2^, Zhu, J^1^, Wyatt, M^1^, Safro, I^4^, Shtutman, M^1^.

*^1^Department of Drug Discovery and Biomedical Sciences, College of Pharmacy, University of South Carolina, Columbia, SC 29208*.

*^2^Department of Biological Sciences, University of South Carolina, Columbia, SC 29208*.

*^3^Department of Statistics, University of South Carolina, Columbia, SC 29208*.

*^4^School of Computing, Clemson University, Clemson, SC 29631*.

HIV-1 Associated Neurocognitive Disorder (HAND) is a clinically detrimental complication of HIV infection. Viral proteins released from infected cells cause neuronal toxicity. In addition, substance abuse in HIV-infected patients greatly exacerbates the severity of neuronal damage. To repurpose small molecule inhibitors for anti-HAND therapy, we employed AI-based literature mining systems that we developed. All human genes were analyzed and prioritized by the system to find previously unknown targets connected to HAND. The list was narrowed to those with known small molecule inhibitors developed for other applications and lacking systemic toxicity in animal models. Finally, we tested the activity of small molecules targeted against the proteins of five prioritized genes to protect against the combined neurotoxicity of HIV-Tat and cocaine in primary neuronal cultures. Three prevented Tat and cocaine toxicity. The compounds are the FDA-approved drug Amlexanox; Tazemetostat, a selective EZH2 inhibitor in Phase II clinical trials; and Dead Box RNA helicase three inhibitor RK-33. Further, with the analysis of transcriptomics modulations by the compounds, we determined additional candidates. Despite the disparate molecular targets of these drugs, the analysis revealed a common mechanism of neuroprotection, namely that modulation of astrocytes and microglia status prevents the toxicity of Tat and cocaine. These findings show that literature mining provides a novel way to accelerate the possibility of repurposing drugs for novel HAND treatments. Supported by NIH/NADA.

### HIV-1 gp120 enhances glutamate neurotoxicity: Implications for HAND pathogenesis

Liu, JN, MD, PhD^1^, Xie, JY, MS^1^, Dutta, D, PhD^1^, Xiong, H, MD, PhD^1^.

*^1^Department of Pharmacology and Experimental Neuroscience, University of Nebraska Medical Center, Omaha, NE 68198*.

Individuals infected with HIV-1 often develop neurological symptoms collectively termed as HIV-1-associated neurocognitive disorders (HAND). HAND remains prevalent despite combined antiretroviral therapy (cART). In contrast to frank neuronal damage and loss seen in severe form of HAND, more subtle changes on synaptodendritic, neuronal, and neuronal circuitry dysfunction are believed to drive the HAND. We hypothesize that the presence of soluble gp120 in the brain of cART-treated patients may dysregulate excitatory synaptic transmission by modulating glutamate (Glu) from physiological neurotransmitter to pathological excitotoxic substance. To test this hypothesis, we studied effects of gp120 (10-20pM) and glutamate (1μM) on spontaneous miniature excitatory synaptic current (mEPSC) and morphology of dendritic spines in rat cortical neuronal cultures. Our results showed that gp120 and glutamate each along, at low doses, had no significant effects on mEPSCs and dendritic spines, but increased the mEPSC frequency, decreased numbers of dendritic spines and induced neuronal apoptosis when applied in combination. The gp120 enhancement of Glu toxicity was significantly attenuated by T140, an X4 receptor blocker, demonstrating gp120 enhancement of Glu neurotoxicity via chemokine receptor X4. Our results may implicate gp120 alteration of brain Glu functionality from a neurotransmitter to a pathological toxicant in patients treated with cART. Supported by NIH NIDA R01DA50540.

### Association of plasma eicosanoid levels with immune, viral, and cognitive outcomes in people with HIV

Deme, P, PhD^1^, Moniruzzaman, M, PhD^1^, Heaton, RK, PhD^2^, Ellis, RJ, MD, PhD^2^, Moore, DJ, PhD^2^, Letendre, SL, MD^2^, Sactor, NC, MD^1^, Haughey, NJ, PhD ^1^.

*^1^Department of Neurology, Johns Hopkins University School of Medicine, Baltimore, MD 21287*.

*^2^HIV Neurobehavioral Research Program and Departments of Neurosciences and Psychiatry, University of California, San Diego, CA 92093*.

Inflammatory cytokines elevated in people with HIV (PWH) are largely reduced with suppressive ART. However, the impact of ART on eicosanoid metabolism is unknown. Eicosanoids are bioactive lipids derived from the metabolism of arachidonic acid (AA) and other polyunsaturated fatty acids (PUFAs) that play major roles in regulating vascular function, inflammation, and immune function. In plasma from people with HIV (PWH;n=95) 39 of 42 eicosanoids (PUFA derived metabolites) were elevated compared to people without HIV (PWoH;n=25) (5% FDR; α=0.0531). In PWH, elevated eicosanoids were associated with lower current and nadir CD4 counts (5% FDR; α=0.02). Plasma eicosanoid levels were similarly elevated in virally suppressed and not-fully suppressed PWH, and plasma viral loads were positively associated with pro-inflammatory eicosanoids (5% FDR;α=0.0459, and 10%FDR;α=0.10). There were no associations between baseline plasma eicosanoid levels and global deficit score, speed of information processing (SIP), learning, recall or motor function. Seven eicosanoid metabolites (3 DHA, 3 AA, 1 LA) were negatively associated with verbal fluency, 4 eicosanoid metabolites (2 EPA, 2 DHA) were negatively associated with executive function, and a striking 30 eicosanoid metabolites (5 EPA, 7 DHA, 13 AA, 5 LA) were negatively associated with working memory performance. However, these associations did not persist following FDR correction. Our findings suggest that HIV infection is associated with a robust increase in plasma eicosanoids that is not resolved by ART. Supported by This study was supported by the NIH awards AA0017408, MH077542, MH075673, and CHARTER Study HHSN271201000027C.

### Caloric restriction mimetic 2-deoxyglucose reduces inflammatory signaling in human astrocytes: Implications for therapeutic strategies targeting neurodegenerative diseases

Fields, JA, PhD^1^, Vallee, KJ, BS^1^.

*^1^Department of Psychiatry, University of California, San Diego, La Jolla, CA 92093*.

Therapeutic interventions are greatly needed for age-related neurodegenerative diseases. Astrocytes regulate many aspects of neuronal function including bioenergetics and synaptic transmission. Reactive astrocytes are implicated in neurodegenerative diseases due to their pro-inflammatory phenotype and close association with damaged neurons. Strategies to reduce astrocyte reactivity may support brain health. Caloric restriction and a ketogenic diet limit energy production via glycolysis and promote oxidative phosphorylation, which has gained traction as a strategy to improve brain health. However, it is unknown how caloric restriction affects astrocyte reactivity in the context of neuroinflammation. We investigated how a caloric restriction mimetic and glycolysis inhibitor, 2-deoxyglucose (2-DG), affects interleukin 1β-induced inflammatory gene expression in human astrocytes. Human astrocyte cultures were exposed to 2-DG or vehicle for 24 h and then to recombinant IL-1β for 6 or 24 h to analyze mRNA and protein expression, respectively. Gene expression levels of proinflammatory genes were analyzed by real-time PCR, immunoblot, and immunohistochemistry. As expected, IL-1β induced elevated levels of proinflammatory genes. 2-DG reversed this effect at the mRNA and protein levels without inducing cytotoxicity. Collectively, these data suggest that inhibiting glycolysis in human astrocytes reduces IL-1β-induced reactivity. This finding may lead to novel therapeutic strategies to limit inflammation, enhance bioenergetics and treat neurodegenerative diseases. Supported by National Institute of Health.

### Lipid signaling in Alzheimer’s disease pathophysiology

Khan, MM, PhD^1^, Mondal, K, PhD^2^, Xiao, J, MD, PhD^1^, McDonald, M, PhD^1^, Mandal, N, PhD^2^.

*^1^Department of Neurology, College of Medicine, Memphis, TN 38163*.

*^2^Department of Ophthalmology, Hamilton Eye Institute, Memphis, TN 38163*.

Alzheimer’s disease (AD) is the leading cause of dementia, which impacts millions of aging people worldwide. Excessive or uncontrolled inflammation is a common pathophysiological feature for many neurodegenerative disorders, including AD. Bioactive lipids are known to play a significant role in immune responses and inflammatory processes by promoting inflammation and resolving inflammation. Lipids are highly abundant in the dry mass of the CNS, providing important structural and metabolic supports. However, alterations in lipid metabolism can influence the pathophysiology of various neurodegenerative diseases, including AD. We set out to determine the role of bioactive sphingolipids (predominantly inflammatory) and omega-3 polyunsaturated fatty acids (n-3 PUFA, mostly pro-resolving) in AD pathology. We found significantly higher levels of sphingolipids in the hippocampus of 7-month-old 5XFAD mice by lipidomic analysis. In an in vitro assay, when treated with short-chain ceramide, mouse brain-derived mixed neuronal cultures showed a significant increase in inflammatory markers and cell death. However, neuronal cultures developed from mice that produce a higher level of endogenous n-3 PUFA (Fat1 mice) were resistant to Aβ42-induced neurotoxicity. This study suggests an association between sphingolipid accumulation and neuroinflammation in AD mouse brain. Bioactive ceramide can cause inflammation and neuronal dysfunction, an effect that may be offset by higher levels of endogenous n-3 PUFA. These results support an intricate role of lipid signaling in AD pathology.

### HiPSC-microglia: a critical cell type to enable in vitro modeling of neurotoxicity of therapeutic agents and neuroimmune effects relevant to neurodegenerative and neurodevelopmental diseases

McDonough, PM, PhD^1^, Gordon, KL, PhD^1^, Riines, CG, PhD^1^, Gesualdi, J, PhD^2^, Akay-Espinosa, C, MD^2^, Jordan-Sciutto, KL, PhD^2^, Price, JH, MD, PhD^1^.

*^1^Biology, Vala Sciences Inc., San Diego, CA 92121*.

*^2^Perelman School of Medicine, University of Pennsylvania, Philadelphia, PA 19104-6030*.

Microglia participate in neuroimmune response pathways linked to neurodegenerative (e.g., Alzheimer’s Disease, Parkinson’s Disease, HIV-Associated Neurocognitive Disorders, etc.) and neurodevelopmental (Autistic Spectrum Disorders, Bipolar Disorder, Schizophrenia, Epilepsy, etc.) afflictions. However, due to their location within the brain, microglia cannot be isolated from living people for in vitro research, and primary microglia harvested post-mortem tend to rapidly de-differentiate, in culture. Microglial-like cells derived from human induced pluripotent stem cells (hiPSC-MG) represent a potential practical and important alternative to primary microglia for in vitro research, as they can be derived in large batches and their differentiation state can be optimized via appropriate culture protocols. We are characterizing the ability of hiPSC-MG to modify, enhance, or mitigate neurotoxic effects of HIV antiretrovirals in the context of HAND, via the use of automated digital microscopy and computer assisted image analysis of cellular structures (High Content Analysis) and cellular activity (calcium and voltage transients, via Kinetic Image Cytometry). Our data suggests that including hiPSC-MG in cocultures with hiPSC-excitatory neurons may have a protective/nurturing effect against toxicity of elvitegravir (integrase-strand transfer inhibitor) and related compounds. These cells and approaches are likely to have widespread applications in elucidating the pathways associated with neuroimmune responses in neurological disorders. Supported by National Institutes of Health/NIMH R44 MH119621.

### Proteomics and cytokine analysis of host factors associated with COVID-19 disease severity in Puerto Rico

Melendez, LM, PhD^1^, Cantres-Rosario, Y, MS^2^, Rodriguez de Jesus, A, MS^3^, Carrasquillo, K, MS^4^, Mendez, LB, PhD^5^, Roche-Lima, A, PhD^4^, Cantres-Rosario, Y, PhD^1^, Rosario-Rodriguez, L, PhD^1^, Rivera-Nieves, V^6^, Bertran, J, MD^7^.

*^1^Dept of Microbiology and Medical Zoology, School of Medicine/Medical Sciences Campus, San Juan, PR 00935*.

*^2^Translational Proteomics Center, UPR Comprehensive Cancer Center, San Juan, PR 00935*.

*^3^Translational Proteomics Center/Center for Collaborative Research in Health Disparities, Academic Affairs/ UPR Medical Sciences Campus, San Juan, PR 00935*.

*^4^Integrated Informatics Services, Center for Collaborative Research in Health Disparities, Academic Affairs/ UPR Medical Sciences Campus, San Juan, PR 00983*.

*^5^Department of Science & Technology, Ana G Mendez, Carolina, PR 00983*.

*^6^Interdisciplinary Studies/Natural Sciences, University of Puerto Rico Rio Piedras, San Juan, PR 00925*.

*^7^Infectious Diseases, Auxilio Mutuo Hospital, San Juan, PR 00919*.

Severe Acute Respiratory Syndrome virus (SARS-CoV-2) causes coronavirus 2019 disease (COVID-19) ranging from asymptomatic to severe disease. We hypothesize that host factors will determine COVID-19 disease severity. After IRB approval, a total of 120 men and women ages 21-80 yrs were recruited in Puerto Rico. Plasma samples from 22 COVID-19 positive patients with asymptomatic / mild symptoms (n=8), with moderate symptoms (n=10) and hospitalized with severe symptoms (n=4) were collected and compared to COVID-19 matched negative controls (n=8). Cytokines associated with a severe COVID-19 outcome were identified and quantified from plasma using a human cytokine array. Quantitative proteomics was performed to analyze plasma using Tandem Mass Tag (TMT) labeling, Proteome Discoverer, Limma Statistics and IPA. We found a significant decrease of MCP-1 in COVID-19 pos itive compared with negative controls (p<0.05). MCP-1 chemokine has been associated with COVID-19 disease severity in other populations. Basic FGF (bFGF), a chemoattractant cytokine with tissue repair roles was significantly decreased in moderate to severe COVID-19 patients. Proteomics revealed 76 dysregulated significant proteins, 2 upregulated and 54 proteins downregulated. Upregulated proteins in severe disease included hemoglobin and Serum Amyloid P component (SAPC). IPA revealed lipid and metabolic regulator pathways activation, atherosclerosis signaling, and coagulation as activated in severe COVID-19. This study uncovers protein and cytokine markers associated with the severity of COVID-19 in Hispanics. Supported by UPR COVID19, U54MD007600, P20GM103475, U54GM133807, and the UPR Comprehensive Cancer Center.

### Immunomodulatory potential of granulocyte-macrophage colony-stimulating factor (gm-csf) in alpha-synuclein overexpressing mice and Parkinson’s disease patients

Olson, KE^1^, Lu, Y, BS^1^, Namminga, KL, BS^1^, Woods, A, PhD^2^, Joseph, SB, PhD^2^, Mosley, RL, PhD^1^, Gendelman, HE, MD^1^.

*^1^Pharmacology and Experimental Neuroscience, University of Nebraska Medical Center, Omaha, NE 68198*.

*^2^Calibr, Scripps, La Jolla, CA 92037*.

Aberrant innate and adaptive immune responses are linked to neuroinflammation and Parkinson’s disease (PD) progression. Augmentation of peripheral immune regulatory and neuroprotective cells is a novel therapeutic strategy for PD. Granulocyte-macrophage colony-stimulating factor (GM-CSF) has previously been successfully utilized as an immunomodulator in pre-clinical and clinical evaluations. However, its short half-life and limited bioavailability limits it wide-spread clinical use. Therefore, a long-acting GM-CSF formulation (mPDM608) was created and assessed for its therapeutic potential in a preclinical PD model. Administration of mPDM608 to mice overexpressing alpha-synuclein within the substantia nigra resulted in increased levels of CD4+CD25+FoxP3+ regulatory T cells (Treg) and CD11b+Ly6C-Ly6C+ myeloid-derived suppressor cells (MDSC) within spleen and peripheral blood. Treatment also increased nigrostriatal dopaminergic survival and decreased reactive microglia. Therapeutic profiles were comparable to treatment with recombinant GM-CSF. Additionally, clinical evaluation of recombinant GM-CSF was assessed and proved beneficial during a two-year trial period. GM-CSF treatment resulted in improved motor activity coordinate with elevated Treg frequencies and stable immunosuppressive capacity. Together, the current findings suggest that use of long-acting GM-CSF would improve PD outcomes. Supported by MJFF and NIH.

### Neuroprotective role of IL-2 in expanding regulatory T-cells in Parkinson’s disease

Saleh, M, BS^1^, Marcovic, M, PhD^1^, Yeapuri, P, PhD^1,2^, Namminga, KL, BS^1^, Lu, Y, BS^1^, Olson, KE, PhD^1^, Gendelman, HE, MD^1^, Mosley, RL, PhD^1^.

*^1^Pharmacology and Experimental Neuroscience, University of Nebraska Medical Center, Omaha, NE 68102*.

*^2^Pharmaceutical Sciences, University of Nebraska Medical Center, Omaha, NE 68102*.

Increasing the number and/or function of regulatory T cells (Tregs) is a therapeutic strategy that seeks to attenuate the neuroinflammatory cascade responsible, in part, for the progression of neurodegeneration in Parkinson’s Disease (PD). Enhancing peripheral Treg populations can interdict in the ongoing neuroinflammatory process and spare dopaminergic neurons from unfavorable autoimmune reactivity in the brain. Herein, we report the neuroprotective role of interleukin-2 (IL-2) through expansion and transfer of Treg cells in 1-methyl-4-phenyl-1,2,3,6-tetrahydropyridine (MPTP) intoxicated mice. For these studies, donor mice were injected intraperitoneally (IP) with low-dose IL-2 (2.5 x 104 IU) over a course of 5 days to increase Treg populations. The resulting Treg cells were harvested and adoptively transferred to recipient mice lesioned with MPTP. Adoptive transfer of IL-2 induced Tregs were found to elevate tyrosine hydroxylase (TH)+ dopaminergic neuronal survival in the striatum and nigra by 74% and 51% respectively when compared to MPTP control. Furthermore, IL-2 treatment was accompanied in tandem by a 3-fold increase in CD4+ CD25+ FOXP3+ cell populations in the IL-2 group over Phosphate Buffered Saline (PBS) control. The implication of these findings further corroborates the utility of immune modulation for the treatment of neurodegenerative disorders and may warrant further research or development of an optimized IL-2 therapy for the purpose of favorable immunological phenotype shift in PD and afford beneficial outcomes accordingly. Supported by National Institutes of Health grant T32 NS105594, R01 NS36126(HEG), RO1 NS034239 (HEG and RLM); P01 MH64570; P30 MH062261 .

### Dynamic role of astrocyte-derived extracellular vesicles in HIV associated neurological disorders

Sil, S, PhD^1^, Chemparathy, D, PhD^1^, Buch, S, PhD^1^.

*^1^Department of Pharmacology and Experimental Neuroscience, University of Nebraska Medical Center, Omaha, NE 68198*.

Although cART usage has increased the lifespan of HIV+ individuals, paradoxically, its dependence is also associated with increased risk of Alzheimer’s-like pathology, as a comorbidity of HIV-Associated Neurocognitive Disorders (HAND). Based on our previous findings that astrocytes play a major role in HIV Tat-mediated amyloidosis, & since amyloids can be released in extracellular vesicles, we sought to assess whether HIV-1 Tat stimulated astrocyte derived EVs (ADEVs) containing toxic amyloids could lead to neuronal injury invitro & synaptodegeneration & cognitive impairments when administered in the brains of naïve mice. Our previous studies have demonstrated the role of HIF-1α as an upstream regulator of HIV1-Tat mediated astrocytic amyloidosis. We thus hypothesized that blocking HIF-1α could likely mitigate neuronal injury. Rat hippocampal neurons exposed to Tat-ADEVs carrying amyloids led to alteration of dendritic arborization, spines, abundance of spine sub-types, synaptic proteins & post-synaptic currents. Silencing of astrocytic HIF-1α not only reduced the numbers of ADEVs & amyloid cargoes, but also ameliorated Tat-ADEV induced neuronal injury. Interestingly, injection of Tat-ADEVs in the hippocampus of naïve mice, resulted in synaptodegeneration & cognitive impairment(s). These impairment(s) were abrogated in mice injected with HIF-1α silenced ADEVs. This study underscores the role of amyloid carrying ADEVs in mediating synaptodegeneration leading to cognitive impairments associated with HAND, and highlights the protective role of HIF-1α in this process. Supported by R25MH080661- John Hopkins University (S. Sil), R21AG069541 (S. Sil) and RO1DA044586 (S. Buch) from National Institute of Health.

### Brain structural correlates of postural instability in HIV infection

Sullivan, EV, PhD^1^, Zahr, NM, PhD^1^, Sassoon, SA, PhD^2^, Pohl, KM, PhD^1^, Pfefferbaum, A, MD^2^.

*^1^Department of Psychiatry & Behavioral Sciences, Stanford University School of Medicine, Stanford, CA 94305*.

*^2^Center for Health Science, SRI International, Menlo Park, CA 94025*.

People living with HIV infection (PLWH) exhibit postural instability on force platform testing, with longer sway paths observed with older age and pedal two-point discrimination impairment. We sought relations between sway path length, performed with and without vision, and age-adjusted MRI-derived cortical and cerebellar volumes in 123 PLWH and 76 age-range-matched controls. As a conservative approach, we first used linear models to identify interactions between group and brain-balance correlations; for significant interactions, we then report simple correlations within PLWH. Sway path without vision correlated with temporal (r=-.27, p=.0025) and occipital (r=-.24, p=.009) cortical and cerebellar (r=-.19, p=.032) volumes. Sway path performed with vision correlated with temporal (r=-.30, p=.0007) and occipital (r=-.25, p=005) volumes. Because 62 PLWH were comorbid for alcohol use disorder (AUD), we reanalyzed the data with the 61 PLWH without AUD (PLWH-AUD). The correlations replicated and were stronger in PLWH-AUD despite the smaller sample: temporal (no vision r=-.43, p=.0006; vision r=-.45, p=.0003), occipital (no vision r=-.38, p=.003; vision r=-.35, p=.006), cerebellar (no vision r=-.30, p=.02; vision =-.28, p=.027) volumes. Brain analyses with 2-point discrimination revealed supramarginal parietal volume interactions with group and significant bivariate correlations with or without AUD: PLWH+AUD (r=-.34, p=.0004) and PLWH-AUD (r=-.43, p=.001). These relations suggest CNS contributions specific to HIV-associated postural instability irrespective of AUD history. Supported by NIAAA/AA017347.

### Poor subjective sleep is associated with cognitive impairment in men but not women living with HIV

Zahr, NM, PhD^1^, Pfefferbaum, A, MD^2^, Sullivan, EV, PhD^1^.

*^1^Psychiatry and Behavioral Sciences, Stanford University School of Medicine, Stanford, CA 94304*.

*^2^Neuroscience, SRI International, Menlo Park, CA 94025*.

Poor sleep undermines the health of persons living with HIV [PLWH]. Prevalence estimates of insomnia are 58% in PLWH relative to 6-33% in the general population. The Pittsburgh Sleep Quality Index (PSQI) is a validated measure of subjective sleep quality. Relations between PSQI-defined insomnia and cognition in PLWH remain unclear and may be modulated by sex. Here, 101 PLWH (35 women), matched with controls (n=56, 25 women) on age, sex, and body mass index (BMI), had lower education, socio-economic status, and premorbid IQ (all p<.0001). A global PSQI score >5 distinguishes good from poor sleepers: PLWH scored 7.5±4.2; controls scored 4.4±2.2. Relative to controls (all p<.0001), PLWH had higher Alcohol Use Disorders Identification Test and Beck Depression Inventory totals; poorer Quality of Life (QoL); and lower cognitive composite scores: executive functioning (EF), attention and working memory (ATWM), verbal and visual learning (VVL), VV memory (VVM), and motor skills (MS). Among all PLWH, higher PSQI correlated with lower premorbid IQ (p=.0008) and QoL (p<.0001); relations with cognitive variables were not significant. Because women LWH had higher PSQI scores than men (p=.04), relations were re-evaluated in women and men LWH separately. Men, but not women LWH showed correlations between higher PSQI scores and lower premorbid IQ (p=.0006), QoL (p<.0001), ATWM (p=.005), VVL (p=.005), and VVM (p=.009). Relations between poor subjective sleep and cognition selective to men PLWH highlight the need to test for sex differences relevant to HIV outcomes. Supported by NIAAA.

## SNIP Early Career Investigators (ECI) Conference Posters:

### Blocking PANX-1 confers neuroprotection to SIV-infected rhesus macaques

Ajasin, D, PhD^1^, Eugenin, E, PhD^1^.

*^1^Department of Neuroscience, Cell Biology and Anatomy, University of Texas Medical Branch, Galveston, TX 77555*.

About 50% of PLWH develop Neuro-HIV which is associated with BBB disruption despite ART. Recently, our laboratory showed ATP levels is significantly higher in PLWH and positive correlation between ATP levels in patients’ sera and severity of Neuro-HIV. Studies have shown neurons are not susceptible to HIV-infection but can be affected by secreted viral proteins like gp120, Tat, and pro-inflammatory proteins from the infected cells in the CNS like microglia, perivascular macrophages, and astrocytes. Prior work from our laboratory shows, HIV-gp120 induces the opening of Panx-1 hemichannel in astrocytes and PBMCs. Also, Panx-1 opening causes increased release and spread of toxic factors such as ATP to neighboring cells and stimulation of pro-inflammation. Therefore, this project focuses on understanding the contribution of Panx-1 to Neuro-HIV and exploring the potential for neuroprotection of PLWH via blocking Panx-1 channels using SIV-infection of Macaca mulatta model. We therefore hypothesize that blocking Panx-1 will significantly reduce viremia, increase CD4 T cells recovery, and confer neuroprotection in the animal model. Our data indicates SIV infection in M. mulatta is unperturbed by blocking Panx-1 but confers neuroprotection. We also observed SIV-infection causes the opening of Panx-1 channels and blocking it shuts down the channel activities. Equally, we observed changes in the different dendritic spine morphology sub-groups which is also suggestive of neuroprotection. Supported by NS105584 and MH126082.

### Insights into sudden cardiac death in persons living with in HIV-1 infection: Dysregulation of type 2 ryanodine receptors by HIV-Tat, efavirenz, atazanavir and ritonavir

Alomar, FA, PhD^1^, Tian, C, PhD^2^, Schroder, E^2^, Bidasee, SR^2^, Alshabeeb, M, PhD^3^, Payne, J, MD^4^, Gendelman, HE, MD^2^, Bidasee, KR, PhD^2^.

*^1^Department of Pharmacology and Toxicology, College of Clinical Pharmacy, Imam Abdulrahman Bin Faisal University, Dammam, 31441*.

*^2^Department of Pharmacology and Experimental Neuroscience, University of Nebraska Medical Center, Omaha, NE 68130*.

*^3^Population Health Research Section, King Abdullah International Medical Research Center, Riyadh, 11481*.

*^4^Department of Medicine/Cardiac Electrophysiology, University of Nebraska Medical Center, Omaha, NE 68198*.

Persons living with HIV infection (PLWH) are 3-5 times more likely to die from sudden cardiac death (SCD) compared to uninfected individuals, and molecular reasons for this remain poorly understood. When cardiac ryanodine receptors (RyR2) become dysregulated, a tachycardia can elicit early and delayed after depolarizations, ventricular arrhythmias and SCD. Herein [3H]ryanodine binding and single channel assays were used to investigate if HIV-Tat, and the first line anti-retroviral drugs, efavirenz (EFV), atazanavir (ATV) and ritonavir (RTV) can bind to and alter RyR2 activity. HIV-Tat, ATV and RTV dose-dependently potentiated [3H]ryanodine binding to RyR2 while EFV decreased [3H]ryanodine binding to RyR2. In single channel assays using purified RyR2 reconstituted into artificial lipid bilayer, cis addition of full-length HIV-Tat (7 - 56 ng/mL), ATV (704 - 8448 ng/ml), EFV (316 - 3792 ng/ml) and RTV (721- 8752 ng/ml) dose-dependently increased the open probability (Po) of RyR2 by increasing its dwell time in the opened state. HIV-Tat (28 and 42 ng/ml) also induced a state of reduced conductance (50% of maximum) that was reversible. Higher concentrations of ATV, EFV and RTV reduced the Po of RyR2. Pre-treating RyR2 with low HIV-Tat (7 ng/ml) to mimic a situation that may occur in PLWH hastened EFV’s ability to close RyR2 and potentiated the ability of ATZ and RTV to open/activate RyR2. From these new data we conclude that HIV-Tat and therapeutic doses ATZ, EFV and RTV are contributing to the increased risk of SCD in PLWH by disrupting the function of RyR2. Supported by R56 HL151602-01A1.

### Analysis of CRISPR/Cas9 as an HIV-1 cure strategy using a targeted sgRNA library-based approach

Berman, R^1,2^, Dampier, W^1,2,3^, Atkins, A^1,2^, Allen, A^1,2^, Pirrone, V^1,2^, Passic, S^1,2^, Ahmed, A^1,4^, Szep, Z^5,6^, Nonnemacher, MR^1,2,7,^ Wigdahl, B^1,2,5,7^.

*^1^Department of Microbiology and Immunology, Drexel University College of Medicine, Philadelphia, PA, USA*.

*^2^Center for Molecular Virology and Gene Therapy, Institute for Molecular Medicine and Infectious Disease, Drexel University College of Medicine, Philadelphia, PA, USA*.

*^3^School of Biomedical Engineering, Science and Health Systems, Drexel University, Philadelphia, PA, USA*.

*^4^Center for Genomic Sciences, Institute for Molecular Medicine and Infectious Disease, Drexel University College of Medicine, Philadelphia, PA, USA*.

*^5^Center for Clinical and Translational Medicine, Institute for Molecular Medicine and Infectious Disease, Drexel University College of Medicine, Philadelphia, PA, USA*.

*^6^Division of Infectious Diseases and HIV Medicine, Department of Medicine, Drexel University College of Medicine, Philadelphia, PA, USA*.

*^7^Sidney Kimmel Cancer Center, Thomas Jefferson University, Philadelphia, PA, USA*.

HIV-1 persistence has been attributed to the latent viral reservoir of integrated proviral DNA in tissues including the peripheral blood, lymphoid tissue, brain, gut, and likely other tissues. An *in silico* prediction algorithm trained on a dataset of patient-derived LTRs isolated from PBMCs led to the discovery of broad-spectrum selected molecular guide RNA (gRNA) targets (SMRT) gRNAs. SMRT gRNAs are predicted to cleave 100% of patient-derived LTR samples as well as a dataset of publicly available patient sequences, which would lead to inactivation or excision of the integrated provirus. *In vitro* study of the SMRT gRNAs revealed high cell viability and cleavage activity through flow cytometry and fluorescent microscopy in TZM-bl, P4R5, and J-Lat cell systems. Sequences of HIV-1 LTR have been obtained from an independent set of patient-derived tissue samples from the NNTC in the brain and spleen with Illumina NextSeq. The PBMC-derived SMRTs showed >90% predicted efficacy for the brain sequences. Furthermore, the Multiple Lentiviral Expression System (MULE) is currently being used to engineer lentiviral constructs to deliver Sa or Sp Cas9 and targeted gRNAs to cells. These lentiviruses will be produced in a library approach to simultaneously measure the ability of thousands of gRNAs to silence HIV-1 expression. Accumulation of this data as well as delivery of a library of gRNAs will allow analysis of Sa and Sp Cas9 enzyme efficiency, gRNA specificity – including consideration of the effect of a leading G nucleotide in the protospacer, and off- and on-target cleavage efficacy. This work is supported by NIMH R01 MH110360 (Contact Multi-PI, BW), NIMH P30 MH092177 (CNAC/CTRSC, Drexel Component PI, BW), NIMH T32 MH079785 (Drexel Component PI, BW).

### Copper as a novel regulator of PERK

Bond, SE, PhD ^1^, Beratan, NR^2^, Bond, MK, BS^2^, Brady, DC, PhD^3^, Jordan-Sciutto, KL, PhD^2^.

*^1^Department of Biochemistry and Biophysics, University of Pennsylvania, Perelman School of Medicine, Philadelphia, PA 19104*.

*^2^Department of Oral Medicine, University of Pennsylvania, School of Dental Medicine, Philadelphia, PA 19104*.

*^3^Department of Cancer Biology, University of Pennsylvania, Perelman School of Medicine, Philadelphia, PA 19104*.

PERK is a transmembrane protein kinase that heads one of the three signaling arms that make up the Unfolded Protein Response (UPR). The UPR is a homeostatic signaling network which senses ER and protein misfolding stress and enacts signaling to lower the cell’s folding load while increasing folding capacity. Activation of the UPR has been noted in patient samples and models of various neurodegenerative conditions, including HIV-Associated Neurocognitive Disorders (HAND). Furthermore, attempts to modulate disease progression by targeting the UPR have suggested that the UPR is dysregulated in such conditions and may play a role in disease pathogenesis. PERK inhibition and activation specifically have been shown to be both beneficial and exacerbating depending on disease context. This is likely due to the biphasic nature of PERK signaling, where PERK activity is required for stress response and tolerance, but can also lead to apoptotic induction depending on the degree of signaling. These findings highlight the need for further delineation of regulators of PERK signaling which may be targeted for therapeutic intervention. Here we present evidence that copper is a novel regulator of PERK, binding of which is required for PERK kinase activity. Copper manipulations can also be used to modulate PERK activity and outcomes in the cell. Finally, we present evidence that copper may be deficient in the brains of people with HIV, suggesting that impaired PERK activity may be leading to chronic UPR activation and contributing to pathogenesis in these patients. Supported by NIH/NIMH/1R01MH109382-01A1.

### In vitro modeling of HIV-1 protein expression in human monocyte-derived macrophages for use in comparative studies with infected human liver macrophages

Brantly, AC, MS^1^, Matt, SM, PhD^1^, Yeakle, K, BS^1^, Bouchard, M, PhD^1^, Gaskill, PJ, PhD^1^, Nonnemacher, MR, PhD^1^.

*^1^Microbiology and Immunology, Drexel University, Philadelphia, PA 19102*.

HIV-1 and HBV cause chronic viral infections and coinfected individuals have an increased risk for developing cirrhosis and hepatocellular carcinoma. The basis for this difference is not clear, but HIV-1- infected T cells and liver macrophages likely contribute to an altered liver microenvironment, exacerbating disease. The heterogenous liver macrophage population, including Kupffer cells and human monocyte derived macrophages (hMDMs), may be a reservoir for HIV-1 as viral RNA and DNA have been detected in patient-derived livers. This project examined hMDMs and liver macrophages infected with HIV-1ADA, a RS tropic virus. The number of p24-containing cells and formation of multi-nucleated giant cells, a hallmark of HIV-1 infection in tissues, particularly the brain, was evaluated using high content imaging on the CX7 High Content scanner. Changes in virion production were evaluated by measuring p24 levels in supernatant using AlphaLISAs. Our results show that an increasing number of infected cells correlates with increased viral production over time and production of multi-nucleated giant cells. Furthermore, treatment of hMDMs with HBV proteins (Sand E antigens) resulted in increased p24 production and percentage of p24-positive cells. This phenotype appears to be reflected in other macrophage types, including tissue resident macrophages. This data is a critical step in establishing a baseline model of HIV-1 infection dynamics to further evaluate changes in HIV-1 infection in cells of a myeloid origin within liver tissues in the absence or presence of HBV. Supported by This work is supported by R0l NS089435 (Contact Multi-PI, MRN), R0l DA039005 (PI, PJG), and R21 Al158024 (Multiple-PIs, MB and M).

### A novel cannabinoid receptor 2 agonist, PM289, may have therapeutic potential at the level of the cerebral microvasculature due to inducible expression of CB2 following experimental traumatic brain injury

Bullock, TA, MS^1^, Leonard, BM, MS^1^, Kahn, JA, BS^1^, Hale, JF, MS^1^, Morales, P, PhD^2^, Jagerovic, N, PhD^2^, Andrews, AM, PhD^1^, Ramirez, SH, PhD^1^.

*^1^Department of Pathology and Laboratory Medicine, Lewis Katz School of Medicine at Temple University, Philadelphia, PA 19140*.

*^2^Instituto de Química Médica, Consejo Superior de lnvestigaciones Científicas, Madrid, 28101*.

Traumatic brain injury is a substantial public health issue for which there are no approved pharmacological interventions. Previous research shows Cannabinoid Receptor 2 (CB2) agonists promote resolution of inflammation in multiple neuroinflammatory disease states. Here, we hypothesized CB2 is a potential pharmacological target at the level of the cerebral vasculature following experimental TBI. First, C57BL/6 mice were given a moderate TBI and CNR2 gene expression was analyzed via qRT-PCR at 4, 8, 24, and 48 hours post injury. Results indicated a 20-fold upregulation of CNR2 in mouse microvessels isolated from the area of impact 24 hours after injury. To subsequently evaluate the translational aspects of targeting CB2 on the cerebral endothelium, we treated human brain microvascular endothelial cells with TNFa and a novel CB2 agonist, PM289. PM289 is a novel chromenopyrazole derivative featuring enhanced solubility and specificity for CB2. Utilizing an in vitro blood brain barrier model (BBB), we analyzed the effects of PM289 on the integrity of the BBB using Electrical Cell-Substrate Impedance Sensing and Western Blot. Results showed that PM289 significantly reduced TNFa induced disruptions to the physical barrier. Moreover, PM289 attenuated TNFa-induced endothelial activation. Taken together, these results show that CB2 is a viable treatment target at the level of the cerebral vasculature following experimental neurotrauma. Ongoing studies are aimed at interrogating the intracellular signaling events responsible for these cerebrovascular protective effects. Supported by 5 R01DA052970-02.

### Targeting human and macaque CCR5 genes using CRISPR-SaCas9 gene-editing platform

Caocci, M, PhD^1^, Burdo, T, PhD^1^, Kaminski, R, PhD^1^.

*^1^Center for Neurovirology and Gene Editing, Temple University, Philadelphia, PA 19140*.

C-C Chemokine receptor type 5 (CCR5) plays a key role in HIV infection as a co-receptor for HIV entry into host cells and cell-to-cell spread. The crucial role of CCR5 in HIV infection was confirmed by the discovery of the delta-32 mutation in the coding region of CCRS. People homozygous for this mutation are resistant to HIV infection. Furthermore, CCR5Δ32/Δ32 hematopoietic stem cell transplantation was found to cure HIV-1 disease in three individuals: “a Berlin patient,” “a London patient,” and most recently, a woman from New York. In this study, we examined the potential application of the CRISPR-SaCas9 platform to edit the human and macaque CCR5 genes. First, the set of candidate SaCas9 specific gRNAs targeting coding sequences of CCR5 genes was selected and tested in primary human and simian PBMCs. Sequencing results were then analyzed using ICE CRISPR analysis tool. The single gRNAs showing the most efficient on-target cleavage activity were then paired, enabling CRISPR-mediated excision of the CCRS gene. Finally, PCR genotyping/Sanger sequencing confirmed the efficient and specific excision of the CCRS gene in primary immune cells. In addition, the same technique was used to verify the lack of off-target activity of selected gRNAs in the human and macaque genomes. These strategies will explore the curative activities of gene editing of cellular receptor to reduce the latent HIV/SIV cell reservoir and delay viral rebound after discontinuation of ART. Supported by CRISPR for Cure Martin Delaney Collaboratory for HIV cure UMl Al164568-01 and cofunded by NIAID, NIMH, NIDA, NINDS, NIDDK.

### Lung remodeling and fibrosis in morphine-dependent, SIV-infected macaques

Chemparathy, DT, PhD^1^, Sil, S, PhD^1^, Callen, S, BS^1^, Chand, HS, PhD^2^, Sopori, M, PhD^3^, Wyatt, TA, PhD^4^, Buch, S, PhD^1^.

*^1^Department of Pharmacology and Experimental Neuroscience, University of Nebraska Medical Center, Omaha, NE 68198*.

*^2^Department of Immunology and Nano-Medicine, Florida International University, Miami, FL 33199*.

*^3^Respiratory Immunology Division, Lovelace Respiratory Research Institute, Albuquerque, NM 87108*.

*^4^Department of Internal Medicine, University of Nebraska Medical Center, Omaha, NE 68198*.

Improved survival of people living with HIV (PLWH) with the advent of anti-retroviral therapy is accompanied with increased prevalence of HIV-associated comorbidities. Chronic lung anomalies are recognized as one of the most devastating sequelae in PLWH. The limited available data describing the lung complications in PLWH with a history of opioid abuse warrants more research to better define the course of disease pathogenesis. The current study was conducted to investigate the progression of lung tissue remodeling in a morphine-dependent rhesus macaque model of Simian Immunodeficiency Virus (SIV) infection. Pathological features of lung remodeling including histopathological changes, oxidative stress, inflammation, and proliferation of fibroblasts was investigated in archival lung tissues from the SIVmac-251/macaque model with or without morphine dependence. Lungs of morphinedependent, SIV-infected macaques exhibited significant fibrotic changes and collagen deposition in the alveolar as well as the bronchiolar region. There was increased oxidative stress, pro-fibrotic TGF, fibroblast proliferation and trans-differentiation, epithelial-mesenchymal transition, and matrix degradation in SIV-infected macaques, which was further exacerbated in the lungs of morphine-dependent macaques. Interestingly, there was increased inflammationassociated remodeling in SIV-infected macaques compared with morphine-dependent SIV-infected macaques. Our findings thus suggest that SIV independently induces fibrotic changes in macaque lungs, and that, this is further aggravated by morphine. Supported by Nebraska Center for Substance Abuse Research (NCSAR).

### Evaluating integrated HIV-1 quasispecies using near full length sequencing of cohort samples in the context of neuroHIV

Collins, ME, BS^1^, De Souza, DR, MS^1^, Dampier, W, PhD^1,2^, Spector, C, MS^1^, Pirrone, V, PhD^1,2^, Passic, S, MS^1,2^, Malone, K^3^, Tillman, S^4^, Szep, Z^3,4^, Wigdahl, B, PhD^1,2,3,5^, Nonnemacher, MR, PhD^1,2,35^.

*^1^Department of Microbiology and Immunology, Drexel University College of Medicine, Philadelphia, PA 19107*.

*^2^Center for Molecular Virology and Translational Neuroscience, Institute for Molecular Medicine and Infectious Disease, Drexel University College of Medicine, Philadelphia, PA 19107*.

^3^Center for Clinical and Translational Medicine, Institute for Molecular Medicine and Infectious Disease, Drexel University College of Medicine, Philadelphia, PA 19107

^4^ Division of Infectious Diseases and HIV Medicine, Drexel University College of Medicine, Philadelphia, PA 19107

^5^Sidney Kimmel Cancer Center, Thomas Jefferson University, Philadelphia, PA 19107

The integrated human immunodeficiency virus type 1 (HIV-1) provirus forms a stable but latent viral reservoir in various tissues. It has been shown that both replication competent and defective proviral sequences exist in the viral reservoir. At least some defective proviruses are able to produce viral proteins. While many individuals achieve undetectable levels of replicating virus as a result of anti-retroviral therapy (ART), current methods for monitoring viral load may not reflect the full landscape. Droplet digital PCR (ddPCR), along with near full length (NFL) amplification and third-generation sequencing (TGS) are valuable tools to evaluate the absence or presence of genes which may contribute to chronic HIV-1 neuropathogenesis and disease. Sequencing the PBMC compartment has shown an approximate 30 percent failure of amplification of the regions encoding viral accessory proteins. We hypothesized that the reason for such differential results could be due to accumulation of replication incompetent defective proviruses. Given these observations, we have used NFL strategies to target provirus and examine the full spectrum of integrated proviruses within the PBMC compartment of individuals in context of cognitive impairment. AmpIicon analyses were conducted utilizing the MinION Nanopore TGS platform. Initial results showed ART-suppressed individuals had mostly defective virus as the predominant species. Future analyses will apply the NFL strategy to determine to examine the potential role of defective genomes in the emergence of neuroHIV. Supported by NIMH P30 MH092177, NIMH T32 MH079785, and R01 NS089435.

### A long-acting tenofovir prodrug suppresses HBV replication for over three months

Das, S^1^, Wang, W^1^, Cobb, D^1^, Ganesan, M^1^, Fonseca, F^1^, Nayan, MU^1^, Deodhar, SS, Hanson, B^1^, Makarov, E^1^, Osna, NA^1^, Edagwa, B^1^, Poluektova, LY^1^.

*^1^Pharmacology and Experimental Neuroscience, University of Nebraska Medical Center, Omaha, NE 68198*.

Background: Tenofovir (TFV) prodrugs (TFV alafenamide, TAF, TFV disoproxil fumarate, TDF) are recommended for chronic hepatitis B (HBV) treatment in HIV co-infection. However, their short half-lives warrant frequent administration, leading to treatment failure by patient non-adherence. To this end, we transformed TFV into a long acting prodrug (NM1TFV) and demonstrated sustained diphosphate metabolite levels in Sprague Dawley rats. [Nat Commun12,5458(2021)]. We now demonstrate, a single intramuscular dose of NM1TFV provides sustained efficacy in HBV mice models over three months with no notable adverse events. Methods: MlTFV was synthesized, and nano formulated (NM1TFV) by high-pressure homogenization. Solid drug nanocrystals of TAF (NTAF) were produced as a control. HBV-infected humanized liver TK-NOG, and HBV transgenic Tg05 mice were administered a single intramuscular injection of 168 mg/kg TFV equivalents of NM1TFV or NTAF. HBV DNA and HBsAg levels in peripheral blood were assessed biweekly. HBV markers HBcAg and HBsAg were evaluated on stained liver sections. Drug levels were quantified by mass spectrometry. Results: NM1TFV suppressed HBV DNA in blood to undetectable levels in all infected humanized mice over twelve weeks with stable human albumin levels. High M1TFV drug concentrations were recorded at 12 weeks in livers and injection sites. NTAF exhibited limited inhibition on HBV DNA replication levels, consistent with undetectable liver drug concentrations at 12 weeks. Enhanced sustained efficacy was demonstrated for NM1TFV compared to NTAF in HBV transgenic mice.

### Verapamil inhibits TXNIP-NLRP3 inflammasome activation and preserves functional recovery after intracerebral hemorrhage in mice

Devlin, P, BS^1^, Ismael, S, PhD^1^, Stanfill, A, PhD^2^, lshrat, T, PhD^1^.

*^1^Department of Anatomy and Neurobiology, The University of Tennessee Health Science Center, Memphis, TN 38163*.

*^2^Nursing-Acute and Tertiary Care, The University of Tennessee Health Science Center, Memphis, TN 38163.*.

Intracerebral hemorrhage (ICH) is the second most common type of stroke, with no satisfactory treatment options. Recent studies from our group and others indicated a potential positive effect of verapamil, a commonly prescribed calcium channel blocker with thioredoxin-interacting protein (TXNIP) inhibitor properties, in ischemic stroke. It is unclear whether there would be a beneficial effect of verapamil administration in ICH. Therefore, this study was designed to determine the neuroprotective effects of verapamil in a murine ICH model. ICH was induced in adult male C57BL/6 mice by stereotactic injection of 0.075 U of collagenase IV into the caudate-putamen or sham surgery. Verapamil (0.15 mg/kg) or saline was administered intravenously at 1 h post-lCH followed by oral (lmg/kg/d) administration in drinking water for 28 days. Motor and cognitive function were assessed using established tests for motor coordination, spatial learning, short- and long-term memory. A subset of animals was sacrificed at 72 hours after ICH for molecular analysis. Verapamil treatment reduced expression of TXNIP and NOD-like receptor pyrin domain-containing-3 inflammasome activation in the perihematomal area. These protective effects of verapamil were associated with decreased proinflammatory mediators, microglial activation, and blood-brain barrier permeability markers, and paralleled less phosphorylated nuclear factor kappa B level. Our findings demonstrate that short as well as long-term low-dose verapamil treatment effectively attenuates motor and cognitive impairments in this ICH model. Supported by Bridge funding (E073005058- Bridge Support-2022) and the National Institute of Health (R01- NS097800) to Tauheed lshrat.

### The interaction of methamphetamine with X4 and R5 HIV-1 strains: Implications for blood-brain barrier dysfunction

Fattakhov, N, PhD^1^, Becker, S, BS^1^, Torices, S, PhD^1^, Park, M, PhD^1^, Toborek, M, MD, PhD^1^.

*^1^Department of Biochemistry and Molecular Biology, University of Miami Miller School of Medicine, Miami, FL 33136*.

Methamphetamine (METH) abuse is a frequent comorbidity among individuals infected with HIV-1 and may be associated with accelerated onset and increased severity of HIV-1-associated neurocognitive disorders (HAND). Although the toxicity of HIV-1 and METH on neurons, astrocytes and brain endothelial cells has been widely studied, there are no reports addressing the influence of co-morbid METH abuse and HIV-1 infection on blood-brain barrier (BBB) pericytes. Therefore, the aim of this study was to investigate the combined effects of METH and HIV-1 strains on BBB function in different in vitro models and whether pericyte infection by HIV-1contributes to these effects. We found that METH in combination with CXCR4-tropic (NL4-3) HIV-1strain or CCRS-tropic (JR-CSF) HIV-1 strain caused a more pronounced decrease in TEER than either condition alone within the contact co-culture model of brain endothelial cells with pericytes. We also determined that the combination of NL4-3 strain with METH additively increased permeability of 10 kDa fluorescein isothiocyanate-dextran across endothelial monolayers in additive manner in non-contact and contact co-cultures with pericytes. Moreover, we determined that METH enhanced viral replication and interleukin-6 release by human brain pericytes infected with NL4-3, but not JR-CSF strain. Collectively, our results indicate that HIV-1 and METH amplify BBB damage through paracrine signals from human brain vascular pericytes and may be dependent on HIV-1 co-receptor tropism. Supported by NIH grants DA044579, DA039576, DA040537, DA050528, DA047157, MH128022, MH122235, MH072567, and HL126559.

### HIV-1 infection in novel humanized microglial mouse induces neurocognitive and mood disorders

Fernandes, A, BS^1^, Samuelson, M, PhD^1^, Guo, L, PhD^1^, Makarov, E, MS^1^, Mathews, S, PhD^1^, Gorantla, S, PhD^1^.

*^1^Department of Pharmacology and Experimental Neuroscience, University of Nebraska Medical Center, Omaha, NE 68198*.

Effective antiretroviral treatment has lessened the severity but not the frequency of Human immunodeficiency virus (HIV)-associated neurocognitive disorders (HAND), ranging from deficits in learning and memory to elevated levels of anxiety and depression. A relevant animal model is needed to better understand the HIV brain infection and molecular mechanisms of HAND. We recently developed a novel humanized glial mouse reconstituted with both the human immune system and microglia. HIV infection in mice mirrored the neuropathology of human disease, such as the presence of multinucleated giant cells, astrogliosis, and synapto-dendritic and myelin loss. Reinforcement of the model for the studies of neuroHIV also requires the display of behavioral deficits as seen in humans. To these ends, mood disorders like anxiety and depression and the deficits in memory and learning were analyzed using the open field test (OFT), sucrose anhedonia test, novel object recognition (NOR), and elevated plus-maze (EPM). HIV-infected mice displayed higher anxiety levels and a slower rate of habituation in OFT suggested by no change in stereotypical grooming and lower percent change in move time. NOR revealed memory deficits with a significantly lower recognition index of the novel object. EPM also showed memory deficits with higher latency to enter the closed arm after repeated trials, and anxiety with lower bouts of head dips. A significant level of depression with lower sucrose preference due to anhedonia was evident. Humanized glial mouse enables studies of neuroHIV in presence of HIV infection. Supported by DHHS/NIH/NIDA/1R01DA054535-01.

### Lysosomal de-acidification inhibits oligodendrocyte maturation: Implications for HIV associated neurocognitive disorders

Festa, LK, PhD^1^, Clyde, AE, BS^4^, Long, CC, BS^5^, Roth, LM, PhD^3^, Grinspan, JB, PhD^2^, Jordan-Sciutto, KL, PhD^1^.

*^1^Department of Basic and Translational Sciences, University of Pennsylvania School of Dental Medicine, Philadelphia, PA 19104*.

*^2^Division of Neurology, Children’s Hospital of Philadelphia, Philadelphia, PA 19104*.

*^3^Abbvie Foundational Neuroscience Center, Abbvie, Cambridge, MA 02139*.

*^4^College of Arts and Science, University of Pennsylvania, Philadelphia, PA 19104*.

*^5^Neuroscience Graduate Group, University of Pennsylvania School of Medicine, Philadelphia, PA 19104*.

Antiretroviral therapy (ART) has led to a reduction in the most severe forms of HIV-associated neurocognitive disorders (HAND); however, cognitive impairment continues to persist in approximately 30- 50% of HIV+ patients. As the severity of HAND has shifted with the advent of ART, so has the pathology; however, white matter alterations continue in the post-ART era. Work in our laboratory has revealed that ARVs from numerous classes, including integrase strand transfer inhibitors (INSTls), inhibit oligodendrocyte (OL) differentiation through a variety of pathways. We sought to determine whether bictegravir (BIC), a frontline INSTI, prevents oligodendrocyte maturation. Using the cuprizone model of demyelination followed by once daily oral gavage of BIC, we observed significant attenuation of remyelination as seen by continued lack of mature Ols (ASPA+) and an increase in oligodendrocyte precursor cells (OPCs; NG2+). To determine the potential mechanism underlying this phenomenon, we focused on lysosomal de-acidification since our previous work has revealed that certain ARVs from other classes disrupt lysosomal pH. Using an in vitro culture system, BIC de-acidified lysosomes in both OPCs and mature Ols, as well as significantly impaired lysosomal degradative capacity without altering overall lysosome number. The TRPMLl agonist, ML-SAl, blocked the ability of BIC to de-acidify lysosomes and to inhibit OL differentiation. Taken together, our data reveal the critical role of proper lysosome acidification in modulating OL differentiation and maturation. Supported by NIH R01MH098742; NIH R01MH126773; NIH T32AI007632.

### A novel 3D microfluidic model of the neurovascular unit for the study of traumatic brain injury and neuroinflammation

Galpayage Dona, KNU, PhD^1^, Galie, PA, PhD^2^, Ramirez, SH, PhD^1^, Andrews, AM, PhD^1^.

*^1^Department of Pathology, Lewis Katz School of Medicine, Temple University, Philadelphia, PA 19140*.

*^2^Department of Bioengineering, Rowan University, Glassboro, NJ 08028*.

Millions experience a traumatic brain injury (TBI) annually, most of which are non-fatal, but which can yield long lasting physical, cognitive, and behavioral disorders. A growing body of evidence suggests that the damage to the blood-brain-barrier (BBB) significantly contributes to TBI pathophysiology. Thus, understanding the biochemical, cellular, and molecular mechanisms that follow TBI, especially with the BBB, can aid in the development of treatments. However, few in vitro models of TBI exist that include vascular elements, fluidic flow, 3D arrangement, and interactions with other cells of the neurovascular unit (NVU). We have developed a novel 3D microfluidic model using light-based bioprinting technology. Scaffolds were constructed using cross-linkable photoinks Polyethylene glycol Diacrylate and Gelatin methacryloyl. Primary brain microvascular endothelial cells were then seeded into vascular lumens and perfused to create fully endothelized 3D branched vascular networks. Moreover, cells of the CNS (ie. astrocytes, neurons etc.) can be embedded into the scaffold to create a NVU. Results show the formation of a barrier, which was disrupted with treatment with TNF-alpha. Additionally, immune-endothelial interactions were observed in real-time. Importantly, we demonstrate that these scaffolds are compatible with a uniaxial stretch device to mimic the primary phase injury after a TBI. Overall, the use of this next-generation microfluidic model of the NVU helps bridge the transability gap for the development of new neuroprotective therapeutic strategies. Supported by NIH K01DA046308 NIH R01DA052970 Shriner’s Hospital for Children 85180-PHl-20.

### Cell-type specific effects of PERK-B associated single nucleotide polymorphisms in HAND pathology

Ghura, S, MS^1^, Bond, S, PhD ^2^, Shi, X ^3^, Akay-Espinoza, C, MD ^3^, Jordan-Sciutto, K.L., PhD ^3^.

*^1^Department of Systems Pharmacology and Translational Therapeutics, University of Pennsylvania, Philadelphia, PA 19104*.

*^2^Department of Biochemistry and Biophysics, University of Pennsylvania, Philadelphia, PA 19104*.

*^3^Department of Basic and Translational Sciences, University of Pennsylvania, Philadelphia, PA 19104*.

Antiretrovirals have increased the life expectancy of people living with HIV, however about 50% of them present with a spectrum of neurocognitive impairments termed HIV-associated neurocognitive disorder (HAND). At the cellular level, increased ER stress leading to potentially maladaptive unfolded protein response (UPR) activation has been reported. A downstream event in UPR is the activation of ER stress sensor PERK, which has been implicated in neurodegenerative disorders including HAND. The gene encoding PERK is known to contain three non-synonymous SNPs in 31% of the general population. These SNPs are genetically linked forming haplotype B (PERK-B) distinct from the more common haplotype (61%), PERK-A. Notably, PERK-B is associated with increased risk for progressive supranuclear palsy. We have found that PERK-B associates with worse cognitive global deficit score in HAND patients. Current literature on the impact of PERK-BSNPs on its activity remain contradictory. We hypothesize that PERK-B has a higher kinase activity, potentially predisposing individuals to neuronal dysfunction. We have developed a novel mouse model expressing PERK-B associated exonic SNPs. In vitro and in vivo data from this model suggest that PERK B activity is increased in myeloid lineage cells, specifically macrophages, while no difference in PERK activation are observed between PERK-A and PERK-Bin neurons. Further, we see evidence of increased kinase activity in liver and pancreas. These findings suggest PERK-B SNPs modulate cell type-specific mechanisms underlying HAND pathology. Supported by NIH.

### Nano-carrier based drug delivery strategy with antiretroviral therapy and nutraceutical to suppress HIV-1 in CNS reservoirs

Godse, S, MS^1^, Zhou, L, MS ^1^, Kumar, A, PhD^1^, Sinha, N, BS ^1^, Kodidela, S, PhD ^1^, Kumar, S, PhD ^1^.

*^1^Department of Pharmaceutical Sciences, University of Tennessee Health Science Centre, Memphis, TN 38163*.

CNS reservoirs primarily perivascular macrophages, and microglia serve as a sanctuary for ongoing HIV-1 replication. Chronic inflammation, cytokine release, and cellular damage resulting due to viral replication at HIV hideouts, culminates in neuronal complications termed as HIV associated neurocognitive disorder (HAND). Major hurdles in the effective eradication of latent HIV-1 reservoirs from CNS and following neuronal complications are attributed to poor penetration of anti-retroviral drugs (ART) drugs into the CNS, neuronal damage induced by viral replication, and ART associated neurotoxicity. Improved drug concentration at the CNS HIV-1 reservoirs and implication of nutraceutical as adjuvant will achieve effective HIV-1 suppression and HAND compared to native drug. In the present study we have developed PLGA nanoformulation with ART01 and NC01. ART01 is FDA approved antiretroviral drug and NC01 is a nutraceutical with proven anti-inflammatory and antioxidant activities. PLGA based formulation is capable of transmigration of ART drug across BBB. These PLGA nano-formulations were characterized for particle size, zeta potential, drug encapsulation, drug compatibility and drug release. Our results showed that the PLGA-ART drug formulation increases the permeability and efficacy of ART drug in in-vitro BBB model. We are in the process of determining biodistribution of PLGA-ART01/PLGA-NC01 formulations in plasma and different organs (liver, spleen, kidney, and brain) in wild type mice. In the end we will study viral suppression of HIV-1 in brain macrophages and microglia. Supported by NIH 1R21MH125670-01A1.

### Therapeutic potential of dietary omega-3 polyunsaturated fatty acids in traumatic brain injury

Grambergs, R, BS^1^, Del Mar, N, MS^3^, Lenchik, N, MD^1^, Mondal, K, PhD^1^, Tahia, F, MS^1^, Tytanic, M, BS^2^, Brush, S, MS^2^, Agbaga, MP, PhD^2^, Reiner, A, PhD^3^, Mandal, N, PhD^1^.

*^1^Department of Ophthalmology, University of Tennessee Health Science Center, Memphis, TN 38163*.

*^2^Department of Ophthalmology, University of Oklahoma Health Science Center, Oklahoma City, OK 73104*.

*^3^Department of Anatomy and Neurobiology, University of Tennessee Health Science Center, Memphis, TN 38163*.

Attenuation of neuroinflammation has emerged as a promising strategy for treatment of traumatic brain injury (TBI). Diffuse axonal injury and microvascular insult secondary to TBI can induce widespread neuroinflammation and microglial activation, leading to long-term neurodegeneration and neuropsychological dysfunction. Metabolites of omega-3 polyunsaturated fatty acids (n-3 PUFA) have demonstrated efficacy in ameliorating inflammation, but n-3 PUFAs have not been studied thoroughly in the context of TBI. Recently, we have shown that Fat1-transgenic mice, which produce a fatty acid desaturase that converts pro-inflammatory n-6 PUFAs such as arachidonic acid (AA) into pro-resolving n-3 PUFAs such as eicosapentaenoic acid (EPA), resist behavioral deficits and have reduced microglial activation and neuroinflammatory markers following blast-induced TBI, relative to wild-type (WT) mice. We hypothesize that high-dose n-3 PUFA supplementation via fish oil-enriched diet can confer similar protection in WT Balb/c mice relative to control diet mice. Before and after exposure to either SO-PSI focal air blast-induced TBI or 0- PSI sham treatment, mice will undergo motor and cognitive assessments by rotarod, tail suspension, and open field activity tests, followed by histochemical analyses of brain tissue. Preliminary data show that brain and plasma of WT mice on n-3 PUFA diet from birth have significantly elevated EPA and decreased AA levels relative to control. We expect that enrichment of n-3 PUFAs will prevent development of TBl-mediated neurological dysfunction in these mice. Supported by National Eye Institute, US Department of Defense.

### Multi-exon guide RNAs targeting HIV-1 proviral DNA for the elimination of latent infection

Hasan, M, MS^1^, Zhang, C, BS^2^, Weight, E, BS^2^, Zaman, L, BS^2^, Deol, P, PhD^2^, Patel, M, MS^2^, Cohen, J, MS^2^, Mathews, S, PhD^2^, Poluektova, L, PhD^2^, Gorantla, S, PhD^2^, Kevadiya, BD, PhD^2^, Dash, PK, PhD^2^, Gendelman, HE, MD^2^.

*^1^Pharmaceutical Sciences, University of Nebraska Medical Center, Omaha, NE 68198-5800*.

*^2^Pharmacology and Experimental Neuroscience, University of Nebraska Medical Center, Omaha, NE 68198-5800*.

Integrated human immunodeficiency virus type one (HIV-1) proviral DNA cannot be eliminated by conventional antiretroviral therapy (ART). Moreover, high HIV-1 mutation rates leading to viral diversity, immune evasion, and antiretroviral drug resistance underlie viral persistence. To these ends, we have developed then validated the TatDE rlNP systems capable of CRISPR RNA cargo carriage. rlNPs carry gRNAs targeting multiple HIV-1 exons (tat1-2/rev1- 2/gp41). TatDE rLNP excised diverse HIV-1 strains and demonstrated its potential as an HIV-1 curative therapy. In the current works, a field-flow fractionation multi-angle light scattering coupled with in-line refractive index and UV detector (FFF-MALS-dRI-UV) method was developed and validated to analyze the LNP batch to batch variability. Small-angle X-ray scattering (SAXS) was used in tandem to analyze LNP properties, including arrangement and porosity evaluations. The structure of the LNPs was visualized, in tandem, by cryo-transmission electron microscopy. These data served to validate the stability and narrow particle distribution. In-vitro studies demonstrated the ability of TatDE rLNPs to excise proviral DNA from latently infected cells. TatDE rLNPs were evaluated in HIV-1 infected humanized mice during antiretroviral drug therapy suppressed conditions. The latter evaluations are underway. Together, our data support “proof of concept" for further developing CRISPR TatDE rLNP therapies for HIV sterilization. Supported by 2R01 NS034239, SR01MH121402, 1R01Al158160, R0l DA054535, POl DA028555, R0l NS126089, R0l NS36126, POl MH64570, P30 MH062261.

### The microbiome implications in morphine withdrawal

Herlihy, B, MS^1^, Praveen, S, PhD^1^, Roy, S, PhD ^1^.

*^1^Neurscience, University of Miami, Miami, FL 33136*.

The ongoing opioid epidemic has caused numerous deaths, increasing rates of hospitalizations, and left millions of people struggling with opioid use disorder. Opioids are commonly prescribed for pain management, however chronic exposure can result in dependence, which requires the presence of the drug to prevent withdrawal. Additionally, the negative symptoms of withdrawal can drive people to seek out the substance again to find relief, often contributing to the development and continuation of addiction. Even though opioid use disorder is a growing problem, there are few therapeutic strategies to combat addiction. This study investigated the microbiome as a potential therapeutic target for morphine withdrawal, since chronic morphine treatment causes dysbiosis of the microbiome, and this microbial shift has been shown to contribute to the development of morphine tolerance. Results showed that depleting the microbiome with an antibiotic cocktail caused a shift in the timing of peak withdrawal severity, with antibiotic treated mice displaying the most severe withdrawal symptoms at 6hrs after morphine pellet removal, as compared to 12hrs for water treated controls. This also resulted in shorter duration of withdrawal for antibiotic treated mice. Additionally, 16sRNA sequencing of fecal samples during the withdrawal process reveal a unique microbiome composition that occurs during peak withdrawal symptoms, suggesting that there are key bacteria contributing to the behavioral symptoms of morphine withdrawal that could be future therapeutic targets. Supported by T32DA045734.

### Targeting prostaglandin E2 receptor EP2 in the treatment of high-risk neuroblastoma

Hou, R^1^, Yu, Y, PhD^1^, Jiang, J, PhD^1^.

*^1^College of Pharmacy, University of Tennessee Heath Science Center, Memphis, TN 38163*.

Neuroblastoma (NB) is the most common childhood extracranial solid tumor. Despite enormous improvements achieved in NB management, the long-term overall survival for high-risk NB remains abysmal. Cyclooxygenase-2/prostaglandin E2 (COX-2/PGE2) cascade fosters an inflammation-enriched microenvironment in NB, promoting tumor proliferation and invasion. Our study aims to elucidate the specific downstream PGE2 receptor (EP) subtype that is directly involved in promoting NB growth, and to evaluate the feasibility of inhibiting the PGE2 receptor as a new strategy to treat high-risk NB. By analyzing gene expression profiles of the COX-2/PGE2/EP cascade from five major NB patient datasets on R2 platform, we identified PGE2/EP2 signaling axis is highly associated with the aggressiveness of high-risk NB. Concordantly, a time resolved fluorescence resonance energy transfer (TR-FRET) assay indicated EP2 receptor is the culprit Gas-coupled receptor that induces cAMP signaling in high-risk NB. Our recently developed selective and bioavailable small-molecule EP2 antagonists have demonstrated potent suppressive effects on the tumor growth and associated angiogenesis in human NB xenografts mice models. These outcomes were further confirmed using NB cell lines in which the EP2 receptor is abated by CRISPR/Cas9-mediated genome editing. Collectively, our data suggest that the PGE2/EP2 pathway contributes to the growth and malignant potential of high-risk NB. Inhibition on EP2 receptor by our drug-like compounds might provide a novel therapeutic strategy for this lethal disease. Supported by National Institutes of Health (NIH)/National Institute of Neurological Disorders and Stroke (NINDS) Grants R21NS109687.

### HIV-1 tat-mediated microglial activation involves a novel ferroptosis signaling

Kannan, M, PhD^1^, Periyasamy, P, PhD^1^, Buch, S, PhD^1^.

*^1^Pharmacology and Experimental Neuroscience, University of Nebraska Medical Center, Omaha, NE 68198*.

Despite the efficacy of cART in controlling viremia, the CNS continues to harbor viral reservoirs. The persistence of low-level virus replication leads to early viral proteins accumulation, including HIV-1 Tat protein. Understanding the molecular mechanism(s) by which viral proteins, such as HIV-1 Tat, can activate microglia is thus of prime importance. In this study, we demonstrated the role of acyl-CoA synthetase 4 (ACSL4)-mediated ferroptosis in HIV-1 Tat-mediated microglial activation and neuroinflammation. Our results showed that exposure of mouse primary microglial cells to HIV-1 Tat resulted in an induction of ferroptosis, which was characterized by increased levels of ACSL4, oxidized phosphatidylethanolamine, lipid peroxidation, the labile pool of iron, ferritin heavy chain-1, mitochondrial membrane rupture, elevated proinflammatory cytokines with a concomitant decrease in glutathione peroxidase-4 levels. Both pharmacological inhibitors (ferrostatin-1 and deferoxamine) and gene silencing approaches using ACSL4 siRNA further validated the critical role of ferroptosis in HIV-1 Tat-mediated microglial activation and neuroinflammation. Bioinformatics analyses identified miR-204 as an upstream modulator of ACSL4, which was further confirmed using argonaute immunoprecipitation. These in vitro findings were also validated in the prefrontal cortices, striatum, and hippocampus of HIV-1 transgenic rats. Overall, this study underscores a novel role of miR-204/ACSL4-mediated ferroptosis in HIV-1 Tat-mediated microglial activation and neuroinflammation. Supported by Nebraska Center for Substance Abuse Research.

### Extracellular vesicles released from macrophages modulates IL-1β in astrocytic and neuronal cells

Kodidela, S, PhD^1^, Kumar, A, PhD^1^, Sinha, N, MS^1^, Zhou, L, MS^1^, Godse, S, MS^1^.

*^1^Pharmaceutical Sciences, The University of Tennessee Health Science Centre, Memphis, TN 38103*.

Background: We have recently demonstrated that long-term exposure of cigarette smoke condensate (CSC) to HIV uninfected (U937) and infected (U1) macrophages are known to induce packaging of pro-inflammatory molecules, particularly IL-1β, in extracellular vesicles (EVs). Therefore, we hypothesize that exposure of EVs derived from CSC treated monocyte/macrophages to CNS cells can increase their IL-1βlevels, contributing to neuroinflammation.

Methods: The U937 and U1 differentiated macrophages were treated once-daily with CSC (lOµg/ml) for 7-days. After 7 days of treatment, the EVs isolated from 1ml media of these macrophages were treated to 1ml of media containing human astrocytic (SVGA) and neuronal cells (SH-SYSY) once daily for 3 days and 2 days, respectively, in the presence of CSC. At the end of the treatment, we examined the protein expression of superoxide dismutase-1 (SODl), catalase (CAT), and IL-1β using western blot. The differences in the relative expression of protein levels between groups were compared using ANOVA, and p<0.05 is considered significant. Results: We observed that the U937 cells have lower expression of IL-113compared to their respective EVs, confirming that most of the produced IL-1βare packaged into EVs. IL-1β protein expression was higher in the SVGA (p<0.05) and SH-SYSY cells that received EVs from U937 and Ul macrophages than those that received no EVs in both control and CSC groups. Conclusion: The increased IL-1βlevels via EV-mediated cell-cell interactions could contribute to neuroinflammationin uninfected and HIV-infected settings. Supported by Funding from the NIH grant DA047178 (Santosh Kumar).

### Effect of endothelial targeted dual antioxidant enzyme conjugate therapy in traumatic brain injury and neuroinflammation

Leonard, BM^1^, Bullock, TA^1^, Shuvaev, VV^2^, Andrews, AM, PhD^1^, Muzykantov, VR, PhD^2^, Ramirez, SH, PhD^1^.

*^1^Department of Pathology & Laboratory Medicine, Lewis Katz School of Medicine at Temple University, Philadelphia, PA 19140*.

*^2^Institute for Translational Medicine and Therapeutics, University of Pennsylvania, Philadelphia, PA 19104*.

Oxidative stress responses following TBI are key contributors of the secondary injury phase. High-energy reactive oxygen and nitrogen species (ROS/RNS) potentiates immune responses, compromises nearby healthy CNS tissue, and disrupts blood brain barrier (BBB) function. Therefore, we hypothesize that attenuating ROS/RNS at the vasculature after TBI will result in improved BBB integrity. Utilizing amino chemistry, dual conjugates (dual AOE/ICAM) consisting of the antioxidant enzymes, superoxide dismutase 1 (SOD1) and catalase, were covalently linked to anti-ICAM-1 Ab. In order to determine that generating the conjugate did not alter AOE function, endothelial cells were exposed to increasing concentrations of hydrogen peroxide (H2O2) and SIN-1. Concentrations added for 1h, ranged from luM to lmM and 200uM to lmM for H2O2 and SIN-1 respectively. Dual AOE/ICAM conjugates at a concentration of 100ng/ml were then added for an hour. Using the Seahorse Mito Stress Assay, our results show that dual AOE/ICAM effectively mitigated mitochondrial stress due to oxidative damage. Measured by oxygen consumption rate (OCR), compared to untreated, the dual AOE/ICAM show significant improvements in OCR against H2O2 (p= 0.0018) and SIN-1 (p<0.0001). Next, to simulate the oxidative stress response from mechanical injury, a Cytostretcher (CuriBio) with endothelial cells was used. Addition of the conjugates following a 20% mechanical strain significantly reduced oxidative stress which promoted an accelerated recovery and inhibition of cellular activation. Supported by Shriners Hospitals for Children.

### Methamphetamine and SARS-CoV-2 induced inflammasome activation

Machhar, JS, MS^1^, Bedi, H, MD^1^, Abbasi, S, MD^1^, Reynolds, JL, PhD^1^, Mahajan, SD, PhD^1^.

*^1^Department of Medicine, Division of Allergy, Immunology, and Rheumatology, Jacobs School of Medicine and Biomedical Sciences/University at Buffalo, Buffalo, NY 14203*.

Methamphetamine (METH) is an addictive stimulant drug that causes long term neurotoxic effects. A significant increase in METH abuse has been reported during the COVID-19 pandemic and SARS-CoV2 infected METH abusers are likely to develop significant CNS cytotoxicity and associated neuropathology. Human microglia, which are the immune cells of the CNS, are activated and involved in a robust neuro-inflammatory response to the SARS-CoV-2 spike protein. METH is known to cause significant neurotoxicity and SARS-CoV-2 promotes NLRP3 inflammasome activation to induce hyper-inflammation, therefore our goal was to examine the role of NLRP3 mediated inflammatory response in METH± SARS-CoV-2 mediated neurotoxicity. We examined the gene expression of members of the NLRP3 signaling pathway in human microglia treated with METH ± SARS-CoV-2 spike protein in-vitro using real time-QPCR. Additionally, we examined the effects of METH ± SARS-CoV-2 spike protein on reactive oxygen species (ROS) production quantitated using CM-H2DCFDA reagent from Invitrogen™ and changes in expression of oxidative phosphorylation (OxPhos) complexes that can lead to mitochondrial dysfunction and accelerated ROS generation. Our results showed that METH± SARS-CoV-2 spike protein resulted in a significant increase in NLRP3 gene expression and ROS production as compared to the untreated control. METH± SARS-CoV-2 induced changes in OxPhos expression by western blot analysis are ongoing. We anticipate that these studies, will help highlight mechanisms that underlie the METH± SARS COV2 induced neuropathology. Supported by SR01DA047410-02.

### A triple humanized blood and brain mouse to evaluate astrocyte and microglial HIV reservoirs

Mathews, S, PhD^1^, Makarov, E, MS^1^, Guo, L, PhD^1^, Wu, L, MD^1^, Poluektova, L, MD, PhD^1^, Gorantla, S, PhD^1^.

*^1^Department of Pharmacology and Experimental Neuroscience, University of Nebraska Medical Center, Omaha, NE 68198*.

Understanding microglia and astrocytes, the main targets of HIV infection and initiators of HAND, remains elusive due to the lack of rodent models. A novel triple humanized mouse with a human hematolymphoid and brain glial system is developed by transplanting human interleukin-34 transgenic NOG pups with autologous human neural progenitors and hematopoietic stem cells to facilitate the development of human immune system and glia. After 8 weeks of HIV-1 infection, human cells were isolated using human HLA-ABC magnetic beads from infected and control brains for single-cell RNA sequencing using 10X genomics. Reads were aligned to human and HIV reference genomes and analyzed by Partek Genomics Suite. The new mouse model has all HIV target cells: the human immune system, microglia, and astrocytes confirmed by flow cytometry and immunohistology. Analysis of 11837 human cells from HIV-1 infected and control mouse brains identified 13022 genes. Cell clustering identified 15 distinct clusters with unique genetic profiles. Microglia harboring complete HIV or partial HIV-RNA were identified. Astrocyte clusters were mainly positive for HIV nef or pol RNA. Cellular pathway analysis of differentially expressed genes (p<0.05) from HIV positive, HIV exposed, and naive cells showed altered interferon signaling, natural killer cell signaling, and increased cytokines indicating immune activation. These mice allow assessing molecular and functional signatures of glial HIV reservoirs and generate single-cell atlas to reveal associations with HIV-neuropathology and therapeutics. Supported by NIH/NIDA 1R33DA041018-01.

### Collaborative oral history and the COVID-19 pandemic’s impact on faith communities

Meigs, DD^1,2^, Alexander, J^3^, Loven-Crum, D^4^, Daoudi, MJ^5^, Khan, AS, MD^6^, Goldberg, W^3^, Gendelman, HE, MD^1^.

*^1^Department of Pharmacology and Experimental Neuroscience, University of Nebraska Medical Center, Omaha, NE 68198*.

*^2^University of Nebraska at Omaha, Department of Sociology and Anthropology, Omaha, NE 68182*.

*^3^Temple Israel, Tri-Faith Initiative, Omaha, NE 68144*.

*^4^Countryside Community Church, Tri-Faith Initiative, Omaha, NE 68144*.

*^5^American Muslim Institute, Tri-Faith Initiative, Omaha, NE 68144*.

*^6^College of Public Health, University of Nebraska Medical Center, Omaha, NE 68198*.

Communities of faith provide a lens for examining the societal impact of COVID-19’s lingering global pandemic. Authors of a perspective-type manuscript explore the pandemic’s societal impact through oral history interviews conducted at two key points of the pandemic: 1.) before the availability of COVID-19 vaccines, and 2.) two years into the pandemic. The manuscript uses a methodology of collaborative oral history with authorship shared between interviewer and interview subjects. Participants in the collaborative oral history include faith leaders of Jewish, Christian, and Muslim communities within the Tri-Faith Initiative of Omaha, Nebraska (Cantor Joanna Alexander of Temple Israel; Administrator Dan Loven-Crum of Countryside Community Church; Imam Mohamad Jamal Daoudi of the American Muslim Institute; Dr. Ali Khan, board chair of the Tri-Faith Initiative and Dean of the University of Nebraska Medical Center’s College of Public Health; and Wendy Goldberg, founding board member and Executive Director of the Tri-Faith Initiative), scientist Dr. Howard E. Gendelman (professor and chair of the UNMC Department of Pharmacology and Experimental Neuroscience), and interviewer/editor Doug Meigs (employed on the administrative staff of the UNMC Department of Pharmacology and Experimental Neuroscience).

### Potential role of ceramide in mild traumatic brain injury (MTBI)-mediated Neuroinflammation

Mondal, K^1^, Cole II, J^1^, Del Mer, N^2^, Joni, M^2^, Daniel, S^3^, Chalfant, C^3^, Reiner, A^2^, Mandal, N^1^.

*^1^Dept. of Ophthalmology, Hamilton Eye Institute, UTHSC, Memphis, TN 38163*.

*^2^Dept. of Anatomy & Neurobiology, UTHSC, Memphis, TN 38163*.

*^3^Dept. of Cell Biology, Microbiology, and Molecular Biology, University of South Florida, Tampa, FL 33620*.

Among the diverse category of lipids, Ceramides (Cer), in the group of bioactive sphingolipids, can act as a pro-inflammatory agent, whereas n-3 polyunsaturated fatty acids (n-3 PUFA) can inhibit and resolve inflammation. In the present study, we tested whether the feeding of PUFA can restrict the elevation of Cer in brain tissue and prevents mild traumatic brain injury (mTBl)-mediated sensory-motor deficits. We used C57/BL6 mice with normal-chow (NC) as control versus mice gavage pre-feed with PUFA (EPA, eicosapentaenoic acid: DHA, docosahexaenoic acid = 2:1 @ 500mg/kg body weight/week) for two weeks, before exposing to left side focal cranial air-blast (50 psi) or sham-blast (0-psi). PUFA feeding was continued for another four weeks after the blast, and at the end of 4 weeks, visual-retinal functional tests were conducted, and the brain tissues were collected for histological and biochemical assays. Lipidomics analysis by LC-MS/MS confirmed a significant elevation of EPA in the plasma and brain tissue of PUFA-fed mice. mTBI was found to elevate Cer in the brain tissues in mice fed with NC, whereas PUFA-fed mice resisted the increase of Cer level as well as the biochemical conversion of Cer from its precursor, sphingomyelin (SM), by inhibiting the enzymatic activity of neutral sphingomyelinase. Immunohistochemical labeling of brain tissues for choline acetyltransferase showed higher levels of atrophy of the oculomotor nucleus in NC-fed blast mice and a concomitant increase in the number of activated microglia (IBA1 immunostaining) in their optic tract compared to PUFA-fed blast mice. This was accompanied by decreased visual and retinal functions in the NC-fed blast mice compared to PUFA-fed blast mice. Our result demonstrates that n-3 PUFA (especially EPA) has a promising therapeutic role in suppressing ceramide biosynthesis and preventing neurodegeneration. Supported by US Department of Defense office of the Congressionally Directed Medical Research Programs (CDMRP) (NM), UTHSC NI Award (KM).

### Brain pericytes and the presence of CNS latent HIV reservoirs

Naranjo, O, BS^1^, Torices, S, PhD^1^, Clifford, P^1^, Tiburcio, D^1^, Toborek, M, MD, PhD^1^.

*^1^Biochemistry and Molecular Biology, University of Miami Miller School of Medicine, Miami, FL 33136*.

The CNS was thought to be protected from HIV infection. However, experiments on microglia and astrocytes indicated that these cells are all capable of active and latent viral infection. Even on antiretroviral therapy (ART), HIV infected individuals are at a higher risk for non-AIDS related co-morbidities, including neurological disease and stroke. BBB pericytes have been shown to regulate brain paracellular and transendothelial fluid transport at the BBB, maintain homeostasis of the CNS microenvironment, and maintain BBB integrity. Additionally, these cells possess the receptor profile enabling latent and active HIV-1 infection. We hypothesize that BBB pericytes are a key cell type for CNS viral reservoirs of HIV. We use a novel HIV reporter, named HIVGKO, that allows for purification of latently infected cells in absence of reactivation. Fluorescence-activated cell sorting was performed to isolate active, latent, and uninfected populations of BBB pericytes. RNAscope analysis we confirm that latent populations have little to no RNA production while active cells do. Transcriptome analysis on these cell populations indicates key differences in angiogenesis, glycolysis, and several inflammatory, and cell cycle pathways. Our transcriptome data in concert with functional assays will help to understand mechanisms that might be triggering pericytes to become latent reservoirs and provide key targets in developing novel protective therapeutics for those suffering from HIV. Supported by NIH R01-MH128022-01, R01 DA050528-02S1.

### Development of long acting bictegravir prodrug library

Nayan, MU, BS^1^, Das, S, MS^2^, Sillman, B, PhD^1^, Hanson, B, BS^1^, Gendelman, HE, MD^1^, Edagwa, B, PhD^1^.

*^1^Department of Pharmacology and Experimental Neuroscience, University of Nebraska Medical Center, Omaha, NE 681982*.

*^2^Department of Pathology and Microbiology, University of Nebraska Medical Center, Omaha, NE 68198*.

Introduction: Bictegravir (BIC) is an essential part of a life-long daily combination antiretroviral therapy (ART) to treat human immunodeficiency virus type one (HIV-1) infection. Despite ART’s effective suppression of HIV-1 replication, regimen adherence, social stigma, viral mutation, and pharmacokinetic (PK) limitations limit long-term efficacy. The generation of long-acting BIC formulation could overcome such treatment challenges. Methods: We synthesized two BIC prodrugs by dimerization and mono-esterification, coined as DiBIC and M2BIC, respectively, then formulated each into aqueous nanocrystals by high-pressure homogenization. The nanocrystals uptake and retention by human monocyte-derived macrophage (MDM) were determined by quantitation of intracellular drug content following a 25 µM drug exposure. PK profiles were evaluated in male Balb/cJ mice after a single intramuscular (IM) injection of the formulations at 45 mg. BIC-eq./kg. dose. Results: The nanocrystals exhibited drug loading, size, and poly-dispersity indices of> 80%, 230-350 nm, and 0.2-0.3, respectively. Single 8hour exposure of MDM to nanoformulated DiBIC (NDiBIC) or M2BIC (NM2BIC) produced sustained intracellular drug content for up to 30 days. Importantly, PK testing of NDiBIC and NM2BIC in Balb/cJ mice produced plasma BIClevels at or above the protein adjusted (PA) 95% inhibitory concentration (PA-IC95) of 162 ng/ml for up to 210 and 300 days, respectively. Conclusion: PK tests support the development of NDiBIC and NM2BIC as once every six-months parenteral antiretroviral therapy. Supported by National Institutes of Health (R01 Al145342; R0l Al158160; P01DA028555; R01NS36126; P01 NS31492; R01NS034239; P01 MH6457.

### The impact of frontline antiretrovirals in an iPSC derived neuroglial model

Nickoloff-Bybel, E, PhD^1^, Angelucci, A, BS^2^, Christian, K, PhD^2^, Su, Y, PhD^2^, Espinoza, C, PhD^1^, Jordan-Sciutto, K, PhD^3^.

*^1^Department of Basic and Translational Medicine, Penn Dental Medicine, University of Pennsylvania, Philadelphia, PA 19104*.

*^2^Department of Neuroscience, Perelman School of Medicine, University of Pennsylvania, Philadelphia, PA 19104*.

*^3^Department of Oral Medicine, Penn Dental Medicine, University of Pennsylvania, Philadelphia, PA 19104*.

Despite the use of antiretroviral therapy, HIV+ individuals still experience neuropathological alterations leading to cognitive decline. While glial activation leading to persistent neuroinflammation is thought to drive many of these changes, studies also suggest that antiretroviraIs (ARVs) can contribute to neuronal injury. Studies in rodents suggest ARVs mediate these effects via oxidative stress and activation of the integrated stress response (ISR); however, whether these mechanisms drive neuronal damage in humans is unclear. Moreover, rodents are not infectible with HIV and cannot be utilized to address interactions between ARVs and HIV. Thus, there is a need for models that can assess how ARVs impact CNS health. We developed an iPSC model consisting of glutamatergic neurons (iN) and astrocytes (iAst) to assess how ARVs impact neuronal and glial health. Cocultures are treated with ARVs and assessed for changes in neuronal and astrocytic health, function, and inflammation. Cells are also processed for RNA sequencing analysis to assess changes in the ISR. Preliminary data indicate that ARVs may increase apoptosis in iNs, but this effect is abrogated in the presence of iAst. Further, MEA analysis shows no changes in iN firing in a pilot study of cocultures exposed to ARVs, suggesting a protective role for astrocytes. Future studies will incorporate microglia to create a tri-culture system, enabling us to assess how the interactions between ARVs and HIV-infected microglia contribute to neuronal dysfunction and inflammation, and develop strategies to mitigate these effects. Supported by NIH.

### The role of vesicle-mediated amyloid-beta on neurogenesis and mitochondrial metabolism in the brain

Osborne, O, MS^1^, Kowalczyk, J^1^, Pierre-Louis, K, BS^1^, Daftari, M^1^, Andras, IE, MD^1^, Toborek, M, MD, PhD^1^.

*^1^Biochemistry and Molecular Biology, University of Miami Miller School of Medicine, Miami, FL 33136*.

Alzheimer’s disease (AD) is the most common form of neurodegenerative disease, affecting one in every eight persons over the age of 65, and around 50 percent of individuals over the age of 85. It is now accepted that elevated brain deposits of beta-amyloid (Aβ) contribute to this neuropathology and cognitive dysfunction in AD. The levels of Aβ40 in the central nervous system (CNS) result from an equilibrium between production in the brain, influx from plasma, and efflux via blood-brain barrier (BBB) efflux transporters. While these processes contribute to the formation of Aβ40 deposits and plaques in AD, emerging evidence indicates that the BBB is a critical interface in the transfer of Aβ40 from the periphery into the brain via vesicular transport. Recent evidence indicates that the role of the BBB in the production and transfer of Aβ40 is important to consider. However, an underlying question about the effect of transfer of A β40 into the brain parenchyma remain unclear. We have previously demonstrated that exposure to EVs carrying A β40 (EV-Aβ40) negatively affects differentiation of NPCs to mature neurons; however, the mechanisms of these effects are unknown. We hypothesize that an underlying cause of altered neurogenesis may be mitochondrial dysfunction that is a prominent feature of AD. The mutual effect of exposing NPCs to Aβ40 that may be trafficked across the BBB, may synergistically induce mitochondrial dysfunction of these cells, which, in turn, leads to the induction of inflammatory responses and aberrant neurogenesis. Supported by NIH R01MH072567.

### Chronic morphine exposure distorts gut-brain interrelationships in SHIV infected rhesus macaques

Pandey, K, MS^1^.

*^1^Pharmacology and Experimental Neuroscience, University of Nebraska Medical Center, Omaha, NE 68198*.

Background: Morphine, a commonly used opioid, is responsible for enhanced SIV/HIV persistence in Central Nervous System (CNS). However, the extent of myeloid cell polarization and viral persistence in different brain regions as well as the additive effects of morphine and SIV on dysregulation of gut-brain systems remains unknown.

Methods: A single daily injection of either morphine (n=4) or saline (n=4) was administered to Rhesus macaques for 9 weeks followed by infection with SHIVAD8EO variants. At necropsy, mononuclear cells were isolated from diverse brain and gut regions. Multiparametric flow cytometry was used to profile myeloid cell polarity/ activation. The results were further validated with indirect immunofluorescence assays. SHIV DNA levels were measured with the digital droplet PCR assay and Luminex assays were used to evaluate soluble plasma/ CSF biomarker levels. Finally, changes in the fecal microbiome were evaluated using the lllumina NovaSeq platform. Results: Based on the Flow Cytometry analysis, morphine exposure led to exacerbated Ml(CD14/ CD16)/ M2 (CD163/ CD206) polarization in activated microglia that spanned across diverse brain regions. This was accompanied by elevated SHIV DNA within the sites of neurogenesis - hippocampus (HIP) and subventricular zone (SVZ). HIP/SVZ CD16+ activated microglia positively correlated with SHIV DNA levels in the brain (r = 0.548, p = 0.042). Simultaneously, morphine dependence depleted butyrate-producing bacteria, including Ruminococcus (p = 0.05}, Lachnospira (p = 0.068) genera and Roseburia_sp._831b (p = 0.068). Supported by NIH grant R01DA041751.

### Biological Optimization of Polymer-based Anti-HIV Drug Delivery towards the Gut Associated Lymphoid Tissue (GALT)

Arkajyoti, P^1^, Garcia, C, Garces, A^1^, Roy, D^1^, Haney, MJ^2^, Batrakova, EV^2^, Chew, SA^1^, Zarei, MM^1^, U Roy^1^.

*^1^Department of Health and Biomedical Sciences, University of Texas Rio Grande Valley, Brownsville, Texas, USA*.

*^2^Department of Molecular Pharmaceutics, Center for Nanotechnology in Drug Delivery, Eshelman School of Pharmacy, The University of North Carolina at Chapel Hill, Chapel Hill, NC, USA*.

According to the Global HIV statistics in 2020, about 37.7 million people were living with HIV and 680,000 people died from HIV infection-related illnesses. Currently, combination antiretroviral therapy (cART) has significantly decreased the disease mortality associated with HIV infection. However, current cART therapy does not efficiently reach the HIV reservoir organs such as gut-associated lymphoid tissue (GALT) due to the lack of drug penetration in these tissues and cells. In this regard, the present study has established polymer-based nanoformulations using F127 and L61 with three clinically available cART drugs such as emtricitabine (FTC), and tenofovir (TNF), and dolutegravir (DLG). The selected formulations were studied for their cytotoxicity toward human cell lines such as Caco-2, HMC-3, and mouse macrophage cell. Through our preliminary characterization, an optimized concentration of polymers F127 and L61 were used for formulation manufacturing purposes. F127 alone and in combination with L61 were used to encapsulate FTC, TNF, and DLG. The safety study of all these formulations indicated that the combination of F127 and L61 formulations showed no significant toxicity, and the majority of all three formulations were observed to be within the size range of nanoparticles. The overall study indicated that all three F127 and L61-based nanoformulations were safe for further *in vitro* characterization. The future study will be to focus on the amount of drug taken into the cell through cell uptake study in different cell lines.​

### Dapansutrile, an NLRP3 inflammasome inhibitor attenuates cyclophosphamide-induced interstitial cystitis

Rakib, A, MS^1^, Kiran, S, PhD^1^, Singh, UP, PhD^1^.

*^1^Department of Pharmaceutical Sciences, College of Pharmacy, University of Tennessee Health Science Center, Memphis, TN 38163*.

Interstitial cystitis (IC)/bladder pain syndrome (BPS), hereafter referred to together as IC, is a clinical syndrome characterized by sterile inflammation of the bladder. Activation of the NLRP3 inflammasome plays a significant role in the development of IC. Here, we investigated the effect that dapansutrile (Dap), an NLRP3 inhibitor, had on the development and severity of IC. we used cyclophosphamide (CYP)-induced experimental IC, which develops a phenotype comparable to clinical IC. We noticed that Dap-treated mice had reduced inflammation scores, frequency of mast cells, neutrophils and UB pathology as compared to mice that received CYP alone. We also noticed an infiltration of T cells in the spleen and iliac lymph nodes (ILNs) and a significant decrease in T cells in the urinary bladder (UB) of Dap-treated mice as compared to CYP alone. In contrast, Dap induces dendritic cells (DCs) as compared to the CYP. Similarly, a reduced level of proinflammatory cytokines was seen in the Dap treated as compared to CYP alone. The alterations in several signaling pathways included decreases in NF-kB, and STAT5 expression in the UB of Dap-treated mice as compared to CYP alone. Together, these results indicate that Dap suppresses IC through reduction of T cells, mast cells, and neutrophils in the UB, but induces DCs might be in part responsible to protection from IC. This study identifies the mechanisms underlying the amelioration of IC by an NLRP3 inhibitor and may provide potential therapeutic targets for the treatment of IC. Supported in part by NIH grant R01 Al140405.

### Traumatic brain injury (TBI) induced biomolecular changes in mitochondrial function

Schmitt, R, MS^1^, Pliss, Artem, PhD^1^, Kusmin, AN, PhD^1^, Qayum, S, BS^1^, Muthaiah, VPK, PhD^2^, Kali1yappan, K, PhD^2^, Abbasi, S, MD^3^, Aalinkeel, R, PhD^3^, Prasad, PN, PhD^1^, Mahajan, SD, PhD^3^.

*^1^Institute for Lasers, Photonics and Biophotonics and Department of Chemistry, University at Buffalo, Buffalo, NY 14226*.

*^2^Department of Rehabilitation Science, School of Public Health and Health Professions, University at Buffalo, Buffalo, NY 14225*.

*^3^Department of Medicine, Division of Allergy, Immunology, and Rheumatology, Jacobs School of Medicine & Biomedical Sciences, University at Buffalo, Buffalo, NY 14203*.

Traumatic brain injury (TBI) is a major cause of death and disability in the United States. The mitochondria, which is the primary site of ATP production, plays a central role in the degree of severity of the TBI. The major cause of TBI-associated brain damage is secondary injury attributed to mitochondrial dysfunction in CNS cells. We recently showed that mild to moderate TBI can result in altered signaling pathways within human brain microvascular endothelial cells (HBMVEC) that constitute the BBB, indicating a greater injury response by the BBB. TBI causes mitochondrial damage leading to impaired brain function, therefore our current investigation examines the changes in the mitochondrial lipidome, cell apoptosis markers, and oxidative stress post TBI. We used an in-vitro blast TBI model that employs a custom acoustic shock tube to deliver blast injury of 23 psi equivalent to a moderate TBI blast to HBMVEC, followed by quantitating mitochondrial dysfunction using Raman spectroscopy, gene expression analysis of key TBI biomarkers, cytokines (IL-1 , IL-6, TNF-a) and caspases-1, -3, -7 using QPCR, and quantitating oxidative stress using reactive oxygen species (ROS) measurement. Our data showed that a moderate TBI blast resulted in significant changes in the mitochondrial lipidome, an increase in Caspase-1, -3, -7 expression and increased oxidative stress as reflected by an increase in ROS. These findings suggest that mitochondrial dysfunction in HBMVEC results in increased oxidative stress and an enhanced inflammatory response that contributes to TBI neuropathology. Supported by Grant# SR01DA047410-02 to SM.

### Role of NLRP6 inflammasome signaling in ethanol-mediated astrocyte activation and neuroinflammation

Singh, S, PhD^1^, Kannan, M, PhD^1^, Periyasamy, P, PhD^1^, Buch, S, PhD^1^.

*^1^Pharmacology and Experimental Neuroscience, University of Nebraska Medical Center, Omaha, NE 68198*.

Alcohol, the most abused drug by adolescents, is known to cause substantial social and economic burdens worldwide. Alcohol intoxication in the brain can lead to motor dysfunction, neuronal injury, cognitive deficits, and inflammation. Several studies have demonstrated that ethanol exposure activates the CNS cells, including astrocytes, leading to the release of proinflammatory cytokines, ultimately resulting in neuroinflammation and cognitive dysfunction. Though microglia-specific NLRP3 inflammasome signaling is well-studied, the involvement of other inflammasomes such as astrocyte-specific NLRP6, remains unexplored. Herein, we sought to investigate the involvement of NLRP6 inflammasome in inducing cellular activation and neuroinflammation in ethanol-exposed human primary astrocytes. Our findings suggest that ethanol upregulated the expression of NLRP6 signaling mediators such as NLRP6, caspase1, and IL11β in human primary astrocytes. Gene silencing studies using NLRP6 siRNA further validated ethanol-mediated activation of NLRP6, cleavage of caspase1, and release of IL11β, in human primary astrocytes. Using microRNA microarray, we also identified an upstream regulatory target, miR-339-5p, of NLRP6 3’ UTR. The binding of NLRP6 3’-UTR with miR-339-5p was determined using miR-target validation assay and miR-339-5p overexpression experiments in ethanol exposed human primary astrocytes. Overall, our findings underscore the role of NLRP6 inflammasome signaling involving miR-339-5p in mediating cellular activation and neuroinflammation using ethanol-exposed human primary astrocytes. Supported by Nebraska Center for Substance Abuse Research (NCSAR).

### Development of mPGES-1 inhibitors for new anti-inflammatory therapies

Sluter, M, BS^1^, Bhuniya, R, PhD^1^, Chen, Y, PhD^1^, Ying, Y, PhD^1^, Yang, CY, PhD^1^, Jiang, J, PhD^1^.

*^1^Pharmaceutical Sciences, University of Tennessee Health Science Center, Memphis, TN 38163*.

Anti-inflammatory drugs have been widely proposed as adjunctive treatment for many neuroinflammatory conditions including epilepsy and stroke. As such, numerous pre-clinical studies support the cyclooxygenase (COX) pathway as a promising anti-inflammatory target. However, COX-2 selective drugs and nonsteroidal anti-inflammatory drugs targeting the COX enzymes are known to produce unwanted cardiovascular and gastrointestinal side effects, thus largely limiting their clinical use. Targeting mPGES-1, the inducible terminal enzyme in the biosynthesis of prostaglandin E2 (PGE2), is thought to provide a safer alternative to COX inhibition because it wouldn’t affect other types of prostanoids that might play beneficial roles in the GI and vascular systems. Using a currently well-known mPGES-1 inhibitor (C3) to create a series of bioisosteres, we identified two novel scaffolds that significantly inhibited PGE2 production under inflammatory conditions with much higher potency than C3 in microglia and neuronal cells. These lead compounds were then evaluated for their in vitro anti-inflammatory properties. Results from these experiments showed a reduction in the expression of COX-2, mPGES-1 and IL-1β at the mRNA level. Compound RB827 was then evaluated in vivo for its efficacy in suppressing neuroinflammation associated with exposure to LPS. Results from this study showed that RB827 was able to significantly suppress several inflammatory genes at the mRNA level in the hippocampus. Further evaluation of these compounds as adjunctive treatment in animal models are ongoing. Supported by NIH/NINDS grants: R01HL141432 (CYY), R01NS100947 (JJ), R21NS109687 (JJ), and R61NS124923 (JJ).

### Alteration in microRNA expression mediates the severity of experimental cystitis

Kiran, S, PhD^1^, Rakib, A, MS^1^, Singh, UP, PhD^1^.

*^1^College of Pharmacy, University of Tennessee Health Science Center, Memphis, TN 38105*.

Interstitial cystitis (IC)/bladder pain syndrome (BPS) is a clinical syndrome characterized by a sterile inflammation of the bladder. The symptoms include suprapubic pain related to bladder filling, accompanied by urgency, frequency, discomfort in the bladder. Autoimmunity has been suspected to play a role in the etiology and pathophysiology of IC. The differential expression of microRNAs (miRNAs) in naïve, activated and effector immune cells suggest that miRNAs also serve as crucial immunoregulators in IC. The cyclophosphamide (CYP) induced experimental IC has provided an excellent model that develops a phenotype comparable to clinical IC with functional and histological alterations confined to the urinary bladder (UB) to study the role of miRNAs in the pathogenesis of IC. In the present study, we noticed differential changes in the UB miRNAs expression in IC mice as compared to control mice. Microarray analysis showed that 107 miRNAs from the UB had a 1.5-fold greater difference in expression of the IC than control. Among them, 3 miRNAs from the bladder were determined by reverse-transcription polymerase chain reaction (RT-PCR) analysis, which has anti- and pro-inflammation properties. We also noticed significant infiltration of macrophages, neutrophils, and mast cells in IC as compared to control. The histological inflammatory scores are also elevated in IC as compared to control. The present study discovers an altered set of new UB miRNAs to identify mechanisms underlying the pathogenesis of IC and may provide a potential biomarker and/or therapy for its treatment. Supported by R0l Al140405.

### Loss of forebrain cholinergic neurons in aged HIV-1 gp120 transgenic rodents is mediated through the p75NTR neurotrophin receptor

Speidell, A, MS ^1^, Mocchetti, M, PhD^1^.

*^1^Department of Neuroscience, Georgetown University Medical Center, Washington, DC 20057*.

Human immunodeficiency virus type 1 (HIV) positive individuals exhibit a constellation of neurological symptoms, termed HIV-associated neurocognitive disorders (HAND). These symptoms affect nearly one in two people living with HIV (PLH), even in the post-combined antiretroviral therapy (cART) era. The continued production and release of viral proteins in the CNS is hypothesized to contribute to HAND neuropathogenesis. Our lab has shown that the HIV envelope protein gp120 alters neuronal proconvertase expression, increasing proneurotrophin abundance in an age-dependent manner and activating the pro-apoptotic proneurotrophin receptor p75NTR. We hypothesized that basal forebrain cholinergic neurons (BFCNs), which highly express p75NTR, are uniquely susceptible to gp120-driven neuronal apoptosis through this receptor. To this end, we crossbred gp120 transgenic (tg) and p75NTR-/- mouse colonies to obtain 3- and 12-month -old (mo) cohorts of wild type (wt), gp120tg, p75NTR+/- and p75NTR+/-gp120tg mice. Our data suggest that only aged gp120tg mice are impaired on a task of extinction of conditioned fear, a complex cognitive behavior subserved by BFCN innervation of the hippocampus and prefrontal cortex. Imaging endpoints obtained from BFCNs in our experimental groups also indicate a decrease in neurite complexity and suggest a reduction in the population of choline acetyltransferase-positive cells in the forebrain of 12mo+ gp120tg animals. Our results show that loss of BFCNs in HAND individuals may underpin the observed collection of neurological impairments in PLH. Supported by NINDS R01 079172, T32 041218, and F31 124490.

### BMAL1 modulates the expression tight junction proteins in brain endothelial cells in response to environmental stress

Teglas, T, PhD^1^, Coker, D, BS^1^, Toborek, M, MD, PhD^1^.

*^1^Department of Biochemistry and Molecular Biology, University of Miami Miller School of Medicine, Miami, FL 33136*.

Polychlorinated biphenyls (PCBs) are lipophilic and highly toxic environmental materials that were useful for industrial products in the past. The chemical stability of PCBs ensures their accumulation in the environment and in human and animal tissues. Chronic exposure effects of coplanar and non-coplanar PCBs are complex and result in the disruption of main organ systems and molecular signaling pathways. In most species, including humans, the circadian clock is ubiquitously present in every cell and is critical to coordinate internal physiological rhythms and behaviors in daily rhythms. At the molecular level, circadian oscillators are based on transcriptional-translational feed-back loops that support organ homeostasis. A pair of transcription factors, namely, brain and muscle ARNT-like 1 (BMAL1) and circadian locomotor output cycles kaput (CLOCK), are central to the molecular clock. Disruption of circadian rhythm may cause several diseases, such as type 2 diabetes, vascular and other metabolic disorders. In this work, we used Bmal1siRNA gene silencing for the modeling of circadian rhythm disruption, followed by exposure of endothelial cells to coplanar and non-coplanar PCBs in different time points. Our results indicate that Bmal1 silencing can potentiate PCB-induced alterations in protein expressions, especially Occludin, ZO-1 and ZO-2 protein. Overall, these results suggest that endothelial cell metabolism can be modulated by environmental factors that can influence that can influence the circadian rhythms. Supported by NIH grants MH128022, MH122235, MH072567, HL126559, DA044579, DA039576, DA04053, DA040537,DA050528,DA04715.

### The role of tunneling nanotubes in HIV spread

Valdebenito S^1^, Ono A^2^, Eugenin, E^1^.

*^1^University of Texas Medical Branch (UTMB), Department of Neuroscience, Cell Biology and Anatomy, University of Texas Medical Branch, Galveston, TX, USA.*

*^2^University of Michigan Medical School, Microbiology & Immunology, Ann Arbor, Michigan, USA.*

TNTs are long-distance intercellular communication systems, that are not present in healthy individuals. TNTs are form by several pathogens including HIV to enable cell targeted viral spread. TNTs allow the transfer of cytoplasmic cargo, organelles, and signaling molecules between the connected cells. Here we showed how TNT, plays key roles in HIV cell-to-cell spread. Our hypothesis is that HIV induces TNT, and as a consequence facilitate the spread of the virus from HIV infected cells to uninfected cells, at early stages of infection. We developed a co-cultured model, to characterize TNTs formation in human primary macrophages and microglia, in response to HIV infection. To quantify the cells HIV infected cells connected by TNTs with the uninfected cell we used live cell and confocal imaging (TNT marker, P24 and DAPI) up to seven days post- infection. We used microinjection of the virus to study to study If only one cell can transfer the virus to surrounding cells. Our results showed that HIV infection induced the formation of TNTs with uninfected cell during the first 7 days post infection. TNTs transfer the virus from HIV infected cells to uninfected cells. Induction of TNT formation with H2O2 treatment and subsequent microinjection of a mature virus, and viral proteins, Nef, gp120, and Vif, did not result in TNT mediated transmission, except gag, suggesting that TNT are highly selective to the transported cargo. In conclusion, TNTs are induced by HIV, also mediates transfer of viral proteins at early stages of infection and are an effective mechanism of viral spread. Funding: This work was funded by The National Institute of Mental Health grant, MH128082, the National Institute of Neurological Disorders and Stroke, NS105584, and UTMB internal Texas funding to E.A.E.

### Methamphetamine enhances HIV infection of human iPSC- derived microglia

Wang, XL, MD^1^, Ho, WZ, MD, MPh^1^, Hu, WH, PhD^2^, Wang, X^1^, Khan, S^1^, Majid, S^1^, Wang, P, PhD^1^.

*^1^Pathology & Laboratory Medicine, Temple University Medical School, Philadelphia, PA 19140*.

*^2^Department of Neuroscience, Temple University Medical School, Philadelphia, PA 19140*.

Because of the overlapping impact of METH, a potent addictive psychostimulant, and HIV on the CNS, it becomes increasingly important to understand the role of interplays between METH and HIV in the pathogenesis of HIV associated neurocognitive disorders (HAND). However, studies of HAND have been hampered by difficulties in collecting primary microglial cells from autopsy or biopsy of HIV patients. Recent success in generating human cells from induced pluripotent stem cell lines (iPSCs) now offers a great opportunity to investigate the impact of METH on HIV infection of microglial cells. In this study, we demonstrated that METH at a concentration as low as 10µM could significantly enhance HIV infection of human iPSC-derived microglia (iMg) and primary human macrophages, which was evidenced by significantly increased expression of the HIV gaggene, p24 protein, and reverse transcriptase activity. Mechanistically, METH suppressed the expression of the intracellular expression of the IFNs and the anti-HIV IFN-stimulated genes (ISGs), particularly, HIV egress inhibitors (viperin and tetherin) in iMg and macrophages. In addition, METH treatment of iMg and macrophages facilitated the expression of the inflammatory cytokines. These data provide the experimental evidence to support the notion that METH use inhibits the specific intracellular immunity against HIV and induces the inflammatory cellular, which facilitates HIV replication in microglia and macrophage. Supported by NIH (DA 51893 and DA 51396 for WZ Ho).

### Transcriptomic profiling of mouse amygdala during opioid dependence and withdrawal by single-cell RNA sequencing

Yan, Y, PhD^1^, Herlihy, B^1^, Boyles, SM^2^, Tao, J, PhD^1^, Hulme, W^2^, Roy, S, PhD^1^.

*^1^Department of Surgery, University of Miami, Miami, FL 33125*.

*^2^John P. Hussman Institute for Human Genomics, University of Miami, Miami, FL 33125*.

Substance use disorder (SUD) is a complex process in which several neurocircuits are disturbed and various brain regions are involved. The amygdala is a region that mediates the withdrawal effect as well as anxiety and depression-like behaviors. However, the specific transcriptional changes in each cell type during SUD is largely unknown. Here we performed single-cell RNA sequencing and classified all cell types in mouse amygdala under opioid dependence and withdrawal conditions. Our data revealed key biological processes, such as immune response, inflammation, synaptic transmission, and mitochondrial respiration, changed in a distinct manner in different cell populations. Dramatic differences were unraveled in the transcriptional profiles between dependence and withdrawal states. Overall, our work has provided a comprehensive dataset of genes, biological pathways and cell-cell interactions for all the identified cell types in the amygdala, thus expanding our limited understanding of brain alterations during SUD, especially at the molecular and cellular levels.

### Post-status epilepticus inhibition of mPGES-1 is neuroprotective

Yasmen, N^1^, Li, L, PhD^1^, Yu, V, PhD^1^, Jiang, J, PhD^1^.

*^1^Pharmaceutical Sciences, University of Tennessee Health Science Center, Memphis, TN 38163*.

Status epilepticus (SE) is the second most common neurological emergency manifested by prolonged

seizures that can cause profound inflammatory responses and cell death within the brain. The current antiseizure drugs aim to stop seizures quickly but cannot prevent SE-induced brain inflammation and injury; nor can they stop the subsequent long-term consequences, such as spontaneous recurrent seizures and behavioral deficits. These consequences are thought to be associated with elevated levels of pro inflammatory mediators including the inducible microsomal prostaglandin E synthase-1 (mPGES-1), which is the terminal synthase of prostaglandin E2 (PGE2). Depletion of mPGES-1 in mice impairs the PTZ-induced seizures and reduces gliosis in the hippocampus. Thus, mPGES-1 might be a target for the treatment of SE. MPO-0063 is a novel mPGES-1 inhibitor that can block both human and murine orthologous enzymes. We investigated the neuroprotective effect of MPO-0063 and its role in diminishing neuroinflammation and gliosis after SE. SE was induced by pilocarpine (280 mg/kg, i.p) in adult mice, allowed to proceed for 1 h, and terminated by diazepam (10 mg/kg, i.p.). Treatment by MPO-0063 (10 mg/kg, i.p) with three doses after SE decreased pro-inflammatory cytokines and chemokines (IL-1β, IL-6, TNF-α, CCL2, CCL3, CCL4, etc.) and lessened gliosis. Neurodegeneration in the hippocampus was also attenuated by treatment with MPO-0063. Our study suggests that mPGES-1 inhibition might provide therapeutic benefits as an adjunctive treatment to reduce the detrimental consequences of SE. Supported by NIH/NINDS grants: R01NS100947, R21NS109687, and R61/R33NS124923.

### Therapeutic potential of suvorexant on intergenerational maternal oxycodone exposure

Yi, J^1^, Koul, S^1^, Pendyala, G^1^, Yelamanchili, SV^1^.

*^1^Department of Anesthesiology, University of Nebraska Medical Center, Omaha, NE 68198*.

The opioid epidemic is a rising public health concern, and pregnant women are a particularly vulnerable population. Our lab has previously shown that maternal opioid exposure has detrimental impacts that persist to the F2 generation, including abnormal genetic expression, increased anxiety, and a difference in phenotypic measurements. Suvorexant (suvo) is a dual hypocretin receptor antagonist that is FDA-approved for the treatment of insomnia. The hypocretin system is involved in the regulation of the sleep/wake cycle, feeding behavior, and notably, addiction. Our previous findings showed that Hcrtr1 is upregulated in both Fl and F2 IUO (in-utero oxycodone exposed) offspring. This project will test the therapeutic potential of suvo to mitigate the lasting impacts of maternal oxycodone exposure. Our preliminary results showed that F2 IUOsuvo animals exhibited significant differences in body weight, body length, and head size circumference at P7 and P14 compared to the control. Furthermore, suvo potentially contributed to a benefit in social behaviors. The IUO-suvo animals were socially active as they had significantly more entries into both the toy and naive chambers at P45, as well as significantly more contacts with both the toy and naïve animal at P28. The next phase of this study will consist of molecular assays, imaging, and further behavior testing including an oxycodone self-administration study. By studying the impact of suvo in IUO-induced developmental deficits, we will get one step closer towards preventing the lasting effects of opioid misuse. Supported by NU Collaboration Initiative Grant.

### Efficacy evaluation of LNPs carrying CRISPR-Cas9 targeting HIV-tat in presence of art in humanized mice

Zhang, C, BS^1^, Hasan, M, BS^1^, Waight, E, MS^1^, Mathews, S, PhD^1^, Kevadiya, B, PhD^1^, Gorantla, S, PhD^1^, Gendelman, H, PhD^1^, Dash, P, PhD^1^.

*^1^Department of Pharmacology and Experimental Neuroscience, UNMC, Omaha, NE68128*.

Elimination of integrated HIV-1 proviral DNA from HIV-infected cells and tissues is required to achieve a virological cure. Viral elimination was achieved by sequential long-acting ART and adeno-associated virus (AAV)-mediated CRISPR-Cas9 in a subset of humanized mice. AAV has limited capabilities to carry short mRNA nucleic acids, given as a single dose, immune site reactions, and co-morbid toxicities. To these ends, CRISPR-HIV-1-specific guide RNAs (gRNAs) TatD and TatE targeting viral Tat, gp41, and rev were created in cargos of lipid nanoparticles (LNP). CD34+ NSG-humanized mice were infected with HIV-1NL4-3 at six months of age. Viral and human immune profiles were tested at 2 weeks post-infection (WPI). All infected mice were placed in four groups: (1) Untreated; (2) ART [dolutegravir, emtricitabine and tenofovir (DTG, FTC and TAF)] treated; (3) CRISPR-Cas9 LNP treated; and (4) ART and CRISPR-Cas9-treatment groups. ART was administered as oral food-pellets starting at 2WPI and maintained until sacrifice. CRISPR-Cas9 LNPs were injected at eight and nine WPI. Animals were sacrificed at ten weeks after viral infection to examine viral and immune profiles, drug levels, tissue viral DNA and RNA levels and excision. Plasma viral load was found at or slightly above the limits of detection and CD4+ T cells were restored at baseline levels for ART and dual-treated groups. Detectable levels of HIV-1 RNA and DNA were found in spleen, lung, liver, and brain tissues and evidence of viral excision. LNPs can be a suitable delivery method for CRISPR-Cas9 targeting for HIV-elimination.

### Nanoparticle-based drug delivery approach with ART drugs to suppress HIV in the CNS reservoirs

Zhou, L, MS^1^, Godse, S, MS^1^, Kumar, A, PhD^1^, Namita, S, MS^1^, Kodidela, S, PhD^1^, Kumar, S, PhD^1^.

*^1^Department of Pharmaceutical Sciences, University of Tennessee Health Science Centre, Memphis, TN 38163*.

The life expectancy of people with HIV has been increased after the introduction of antiretroviral therapy (ART). Although the viral load of HIV patients is not detectable with ART therapy in the peripheral system, a total cure is still not available. With the AIDS progression while on ART, HIV+ patients experience HIV-associated neurocognitive disorders (HAND). The tight junctions and drug efflux transporters in the blood-brain barrier (BBB) as well as metabolizing cytochrome P450 (CYP), especially CYP3A4 enzyme in brain macrophages and microglia restrict the permeability and bioavailability of ART drugs. Hence, this study focuses on developing a more safe and biocompatible drug delivery system that can overcome the limitation of BBB permeability and drug metabolism in CNS cells. In this study, we established a poly Lactic-co-Glycolic Acid (PLGA) nanoparticle-based ART drug delivery system to increase the concentrations of ART drugs in CNS. The ART drug (ART02) was loaded in PLGA by nanoprecipitation method. The physicochemical properties of our nanoparticle product were determined. Our results showed that the PLGA- ART drug formulation increases the permeability and efficacy of the ART drug in in-vitro BBB model. We are in the process of developing pharmacokinetics, especially in the brain, of PLGA-ART02 formulation in wild type mice using both intravenous and intranasal delivery. We will eventually study the viral suppression of HIV in brain macrophages and microglia. Supported by NIH 1R21MH125670-01.

